# Metabolomic similarities between bronchoalveolar lavage fluid and plasma in humans and mice

**DOI:** 10.1038/s41598-017-05374-1

**Published:** 2017-07-11

**Authors:** Charmion Cruickshank-Quinn, Roger Powell, Sean Jacobson, Katerina Kechris, Russell P. Bowler, Irina Petrache, Nichole Reisdorph

**Affiliations:** 1 Department of Pharmaceutical Sciences, University of Colorado Anschutz Medical Campus, Aurora, CO 80045 USA; 2 Department of Biostatistics and Informatics, University of Colorado Anschutz Medical Campus, Aurora, CO 80045 USA; 3 Department of Medicine, National Jewish Health, Denver, CO 80206 USA

## Abstract

This observational study catalogues the overlap in metabolites between matched bronchoalveolar lavage fluid (BALF) and plasma, identifies the degree of congruence between these metabolomes in human and mouse, and determines how molecules may change in response to cigarette smoke (CS) exposure. Matched BALF and plasma was collected from mice (ambient air or CS-exposed) and humans (current or former smokers), and analyzed using mass spectrometry. There were 1155 compounds in common in all 4 sample types; fatty acyls and glycerophospholipids strongly overlapped between groups. In humans and mice, more than half of the metabolites present in BALF were also present in plasma. Mouse BALF and human BALF had a strong positive correlation with 2040 metabolites in common, suggesting that mouse models can be used to interrogate human lung metabolome changes. While power was affected by small sample size in the mouse study, the BALF metabolome appeared to be more affected by CS than plasma. CS-exposed mice showed increased plasma and BALF glycerolipids and glycerophospholipids. This is the first report cataloguing the metabolites present across mouse and human, BALF and plasma. Findings are relevant to translational studies where mouse models are used to examine human disease, and where plasma may be interrogated in lieu of BALF or lung tissue.

## Introduction

Plasma and serum are often utilized in biomarker discovery studies; collection is relatively non-invasive and sufficient volume can be obtained. While peripheral to the actual affected sites of many conditions, such as lung or heart disease, these biofluids provide a rich source of information that can potentially be used to diagnose and treat disease. Conversely, the collection of samples from organs or fluids that are directly affected by disease often requires an invasive procedure, e.g. bronchoalveolar lavage fluid (BALF). Other challenges with biomarker discovery that can be exacerbated when not using plasma include insufficient sample volume, difficulty obtaining appropriate control samples, and low sample numbers. Ideally, the molecular composition of plasma would reflect the state of both healthy and diseased tissue; this would enable researchers to use plasma as a proxy for less accessible samples. However, to date, there are few studies that have compared the overlap in markers between disease/non-disease tissue and plasma. Therefore, the utility of plasma as a proxy or surrogate for disease markers remains largely unexplored.

This situation is of particular interest in biomarker studies focusing on lung disease, where BALF, sputum, exhaled breath condensate, and saliva have all been used as proxies for lung epithelial lining fluid and tissue^1–4^. For example, a recent study used immunoassays to compare the profiles of BALF, bronchial biopsies, serum, and sputum to investigate over 100 markers in 23 healthy smokers and 24 chronic obstructive pulmonary disease (COPD) patients who smoke^5^. While few correlations were found between lung and serum inflammatory cytokines in the context of disease presence or severity, this study did successfully illustrate a substantial overlap between serum, lung, and lung-biofluid protein markers. To our knowledge, no discovery-based proteomics study has shown a significant correlation between markers found in both plasma and lung in the context of COPD severity^6^. This is partially due to the challenges inherent in plasma proteomics, which include poor sensitivity and wide dynamic range. Conversely, several groups, including our own, have used plasma metabolomics to investigate the effects of cigarette smoke (CS) exposure on mice^7^ and humans^8, 9^. A recent study using untargeted metabolomics to analyze serum or plasma from 892 current, former, or never smokers detected dysregulated metabolites belonging to xanthine metabolism, benzoate metabolism, vitamins, and amino acid metabolism^9^. Another study^10^ examined the proteomic and metabolomic profiles of mouse hippocampus tissue from offspring of mice exposed to smoke; phospholipids were found to be dysregulated, among other changes. While significant in terms of plasma and tissue biomarkers that relate to COPD, none of these studies examined metabolites from both plasma and BALF and examined the overlap between mice and humans.

The complexity of COPD and other diseases also necessitates the use of animal models to more thoroughly understand disease mechanisms; this has been accomplished in several studies^11, 12^. Recently, the serum and BALF metabolome were examined during the progression of emphysema in a murine porcine pancreatic elastase model^13^. The authors found a significant relationship between lung-specific L-carnitine and lung function; lung function was restored upon supplementation with L-carnitine. These studies illustrate the utility of animal models; however, no comparison between animal and human samples was performed.

The current study aims to fill this gap by using metabolomics to investigate the overlap between BALF and plasma in both humans and mice; further, it explores how metabolites can change upon exposure to cigarette smoke. Our long-term goal is to determine if specific compounds or classes of compounds in plasma reflect lung health status.

## Results

### Catalogue and overlap of the plasma and BALF metabolomes in mice and humans

**Figure 1:**
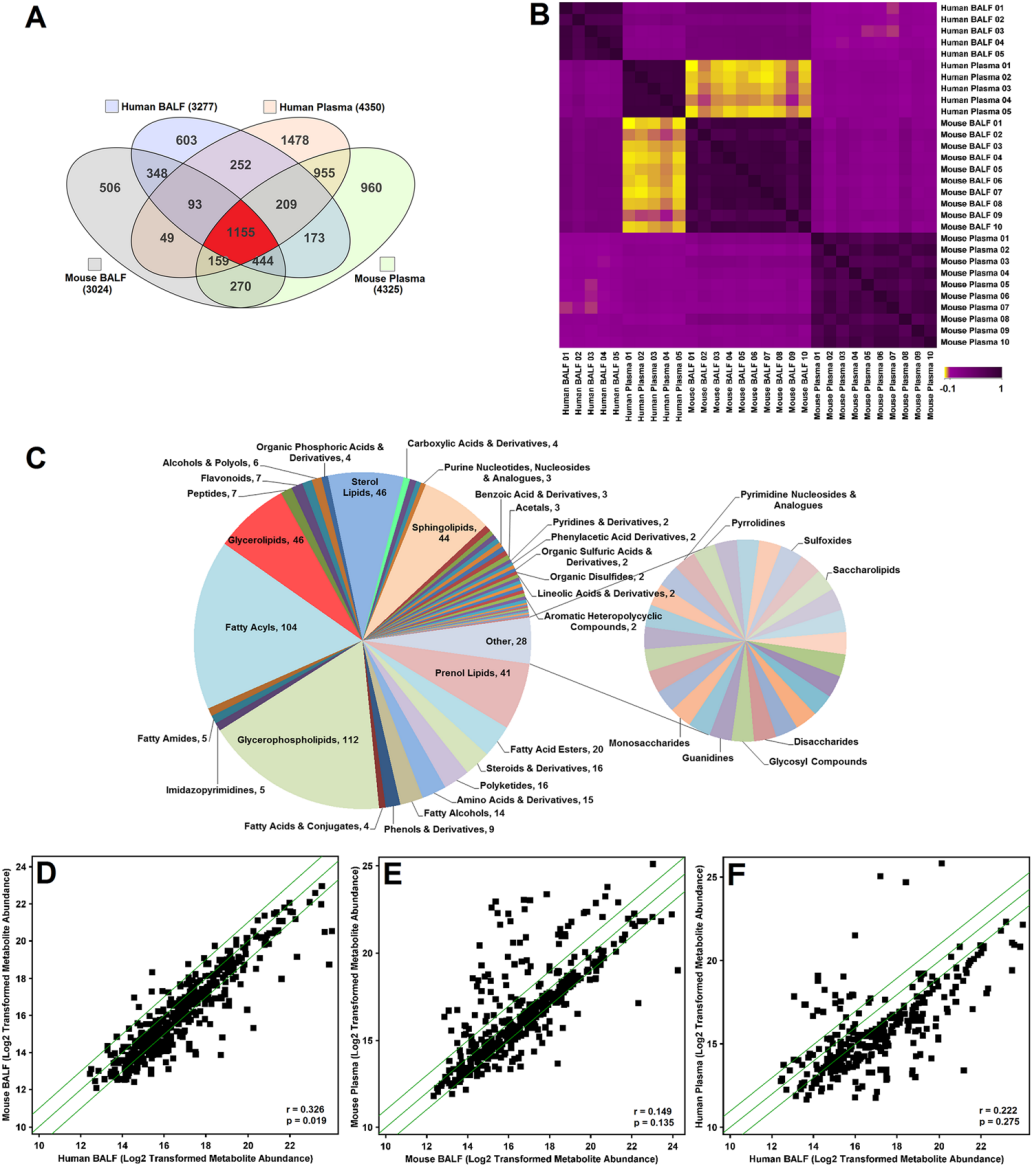
Metabolite relationships across species and biofluids. (**A**) Overlap of metabolites represented with a venn diagram of metabolites identified in both aqueous and lipid fractions filtered for presence in at least 2 samples within each group. (**B**) Spearman correlation matrix of species and biofluid based on detected lipids in the samples, with yellow (r = −0.1) representing a negative correlation, and purple (r = 1) representing a positive correlation. (**C**) Distribution of biochemical classes represented by metabolites that were detected in human and mouse, BALF and plasma (1155) from Fig. 1A. Classes were based on Lipid Maps and HMDB classifications. The number of metabolites corresponding to the overlap is indicated next to the name of the class. (**D**) Scatter plot showing a positive correlation in metabolites between mouse BALF and human BALF. (**E**) Scatter plot showing a positive correlation in metabolites between mouse plasma and mouse BALF. (**F**) Scatter plot showing a positive correlation in metabolites between human plasma and human BALF. In the scatterplots, each square represents an individual metabolite. The metabolites along the diagonal green lines show the strongest positive correlations. The average abundance of each metabolite is scaled between 10 and 24.

We used metabolomics to develop a catalogue of plasma and BALF metabolites from both mice and humans and detected a total of 7,654 unique metabolites in all samples (Fig. 1A); these included all smoking and non-smoking samples. We found more than 4,000 metabolites in human and mouse plasma and approximately 3,000 metabolites in the human and mouse BALF (Fig. 1A). The majority of metabolites in both fluids were lipids with only a minority (~10%) being aqueous metabolites. This is consistent with our previous results^14^ and is likely because lipids are a major constituent of biological membranes^15^.

Overall, there was at least 50% overlap in metabolites between pairs of the four groups (Fig. 1A) as follows: 62.3% of human BALF metabolites were also present in mouse BALF, 67.1% of mouse BALF metabolites were also present in mouse plasma, 52.2% of human BALF metabolites were also present in human plasma, and 57.3% of mouse plasma metabolites were also present in human plasma. There were 2,040 compounds common to mouse and human BALF (Fig. 1A); 1,846 were lipids, of which 1,075 were annotated by database identification; 194 were aqueous molecules, of which 87 were annotated by database identification. Based on these annotations, carnitines, purines, amino acids, peptides, sphingolipids, and glycerophospholipids were common to both the human and mouse BALF samples. Table 1 includes a representative list of these metabolites. A comprehensive list is available in Supplemental Table S4. A total of 2,478 compounds were common to mouse and human plasma (Fig. 1A). Of the plasma metabolites, 2,208 were lipids of which 1,383 were annotated using a database; 270 were aqueous molecules of which 213 were annotated using a database. Many of these are signaling molecules including LysoPCs, ceramides, and diglycerides (Table 1 and Supplemental Table S5). Other common groups of metabolites in mouse and human plasma included carnitines, amino acids, carbohydrates, sphingolipids, steroids, and vitamin D2.

**Table 1:** Representative overlapping metabolites in human and mouse BALF and/or human and mouse plasma.

Compound	Formula	Accession ID	Mouse BALF	Human BALF	Mouse Plasma	Human Plasma	Group & Derivatives
β-D-Galactose	C6 H12 O6	KEGG: C00962	X	X			Carbohydrates
2′-Deoxyguanosine 5′-monophosphate	C10 H14 N5 O7 P	KEGG: C00362	X	X			Purines
Deoxyguanosine	C10 H13 N5 O4	KEGG: C00330	X	X			Purines
Homocysteic acid	C4 H9 N O5 S	KEGG: C16511	X	X			Amino acids
Isopropyl β-D-glucoside	C9 H1 8O6	HMDB32705	X	X			Carbohydrates
Propionylcarnitine	C10 H19 N O4	KEGG: C03017	X	X			Carnitines
Uric acid	C5 H4 N4 O3	KEGG: C00366	X	X			Purines
Δ9-Tetrahydrocannabinol	C21 H30 O2	CAS: 1972-08-3		X		X	Benzopyrans
β-Tocopherol	C28 H48 O2	KEGG: C14152			X	X	Prenol lipids
16,17-Didehydroprogesterone	C21 H28 O2	KEGG: C03207			X	X	Sterol Lipids
Betaine*	C5 H11 N O2	HMDB00043			X	X	Amino acids
CL(76:7)	C86 H154 O17 P2	HMDB57006			X	X	Glycerophospholipids
Creatinine*	C4 H7 N3 O	HMDB00562			X	X	Amino acids
Dihydrothymine	C5 H8 N2 O2	HMDB00079			X	X	Pyrimidines
Glucose*	C6 H12 O6	HMDB03345			X	X	Carbohydrates
Histidine	C6 H9 N3 O2	HMDB00177			X	X	Amino acids
Inosine 2′,3′-cyclic phosphate	C10 H11 N4 O7 P	HMDB11680			X	X	Purines
PA(34:6)	C37 H61 O8 P	LMGP10010130			X	X	Glycerophospholipids
Palmitoylcarnitine	C23 H45 N O4	HMDB00222			X	X	Carnitines
PS(38:0)	C44 H86 N O10 P	LMGP03010705			X	X	Glycerophospholipids
Sorbitol	C6 H14 O6	KEGG: C00794			X	X	Carbohydrates
Taurine*	C2 H7 N O3 S	KEGG: C00245			X	X	Amino acids
(24R)-24-fluoro-1α,25-dihydroxyvitamin D2	C28 H43 F O3	LMST03010011	X	X	X	X	Sterol lipids
21-Deoxycortisol	C21 H30 O4	KEGG: C05497	X	X	X	X	Steroids
Acetylcarnitine*	C9 H18 N O4	HMDB00201	X	X	X	X	Carnitines
C16 Sphinganine	C16 H35 N O2	LMSP01040001	X	X	X	X	Sphingolipids
Carnitine*	C7 H16 N O3	HMDB00062	X	X	X	X	Carnitines
Ceramide (d18:1/18:0)*	C36 H71 N O3	KEGG: C00195	X	X	X	X	Sphingolipids
Cholesterol*	C27 H46 O	KEGG: C00187	X	X	X	X	Steroids
Choline*	C5 H14 N O	KEGG: C00114	X	X	X	X	Cholines
Creatine*	C4 H9 N3 O2	KEGG: C00300	X	X	X	X	Amino acids
DG(34:1)*	C37 H70 O5	LMGL02010307	X	X	X	X	Glycerolipids
Estradiol-17α	C18 H24 O2	LMST02010029	X	X	X	X	Steroids
Glycerol 1-stearate*	C21 H42 O4	CAS: 123-94-4	X	X	X	X	Monoacylglycerol
Hypoxanthine*	C5 H4 N4 O	KEGG: C00262	X	X	X	X	Purines
Leucine*	C6 H13 N O2	KEGG: C00123	X	X	X	X	Amino acids
Linoleyl alcohol*	C18 H34 O	CAS: 1577-52-2	X	X	X	X	Fatty alcohol
LysoPC(16:0)*	C24 H50 N O7 P	HMDB10382	X	X	X	X	Glycerophospholipids
LysoPE(18:0)*	C23 H48 N O7 P	LMGP02050001	X	X	X	X	Glycerophospholipids
MG(18:0)	C21 H42 O4	HMDB11131	X	X	X	X	Glycerolipids
PC(32:0)	C40 H80 N O8 P	HMDB08031	X	X	X	X	Glycerophospholipids
PE(36:4)	C41 H74 N O8 P	HMDB09418	X	X	X	X	Glycerophospholipids
PG(32:0)	C38 H75 O10 P	LMGP04010929	X	X	X	X	Glycerophospholipids
Phenylalanine*	C9 H11 N O2	KEGG: C00079	X	X	X	X	Amino acids
Tyrosine*	C9 H11 N O3	KEGG: C00082	X	X	X	X	Amino acids

This list contains metabolites that were annotated using an in-house database comprised of METLIN, HMDB, Lipid Maps and KEGG. Metabolites were selected randomly from each of the various compound classes. Scores ≥70 out of a possible 100 and mass errors ≤10 ppm were used for annotation thresholds. *Confirmed annotations using tandem MS and matching fragments to reference standards using the NIST14 MSMS spectral database. CL: cardiolipin, DG: diglyceride, MG: monoglyceride, PA: phosphatidic acid, PC: phosphatidylcholine, PE: phosphatidylethanolamine, PG: phosphatidylglycerol, PS: phosphatidylserine.

There were 1,155 metabolites common to all four groups (Fig. 1A), representing 84 biochemical classes (Fig. 1C). In metabolomics, important biological changes may be found in biochemical groups of molecules in addition to individual species. For example, a total of 112 glycerophospholipids and 104 fatty acyls were found in all 4 sample types. Similarly, there were over 120 glycerolipid, sterol lipid, sphingolipid, and prenol lipid molecules that were common between all 4 samples types (Fig. 1C). Conversely, there was relatively little overlap between purines, pyridines, and pyrimidines. This could be a reflection of biology or of platform limitations.

### Metabolite correlations across species and biological fluids

In order to establish levels of congruency between sample types, Spearman’s rank correlation coefficient was used (Fig. 1B). For filtering purposes, this comparison only included molecules that were detected in at least 20% of all samples. This filter level was chosen due to the small sample size, to reduce/eliminate false positives, and to avoid over-filtering the data and potentially missing important metabolites. There was no significant correlation between human BALF and mouse plasma (r = 0.0374, p = 0.794) or between human plasma and mouse BALF (r = −0.0703, p = 0.624). This indicates that these samples have dissimilar metabolomes, in spite of having over 50% metabolites in common (Fig. 1A). Mouse plasma and human plasma were not correlated (r = 0.0975, p = 0.496). Mouse BALF and mouse plasma (r = 0.149, p = 0.135) and human BALF and human plasma (r = 0.222, p = 0.275) were positively correlated but did not reach statistical significance. However, mouse BALF and human BALF were positively correlated (r = 0.326, p = 0.0195). The positive and significant correlation indicates that the samples have similar metabolomes.

We examined the distribution of compounds in the closely correlated biofluids. The metabolites along the green diagonal lines in the scatter plots of mouse BALF and human BALF (Fig. 1D), showed the strongest positive correlations. Similar positive correlation was observed for the mouse plasma and mouse BALF (Fig. 1E). There was weak correlation in the human plasma and human BALF (Fig. 1F). The majority of these compounds are listed in Supplemental Tables S4 and S5. Examples of highly correlating molecules from Fig. 1D–F include phosphatidylinositols (PI), phosphatidylserines (PS), diglycerides (DG), sterol lipids such as cholesterol and Δ8,14-sterol, and fatty acids such as eicosanedioic acid and pentadecyclic acid.

**Figure 2:**
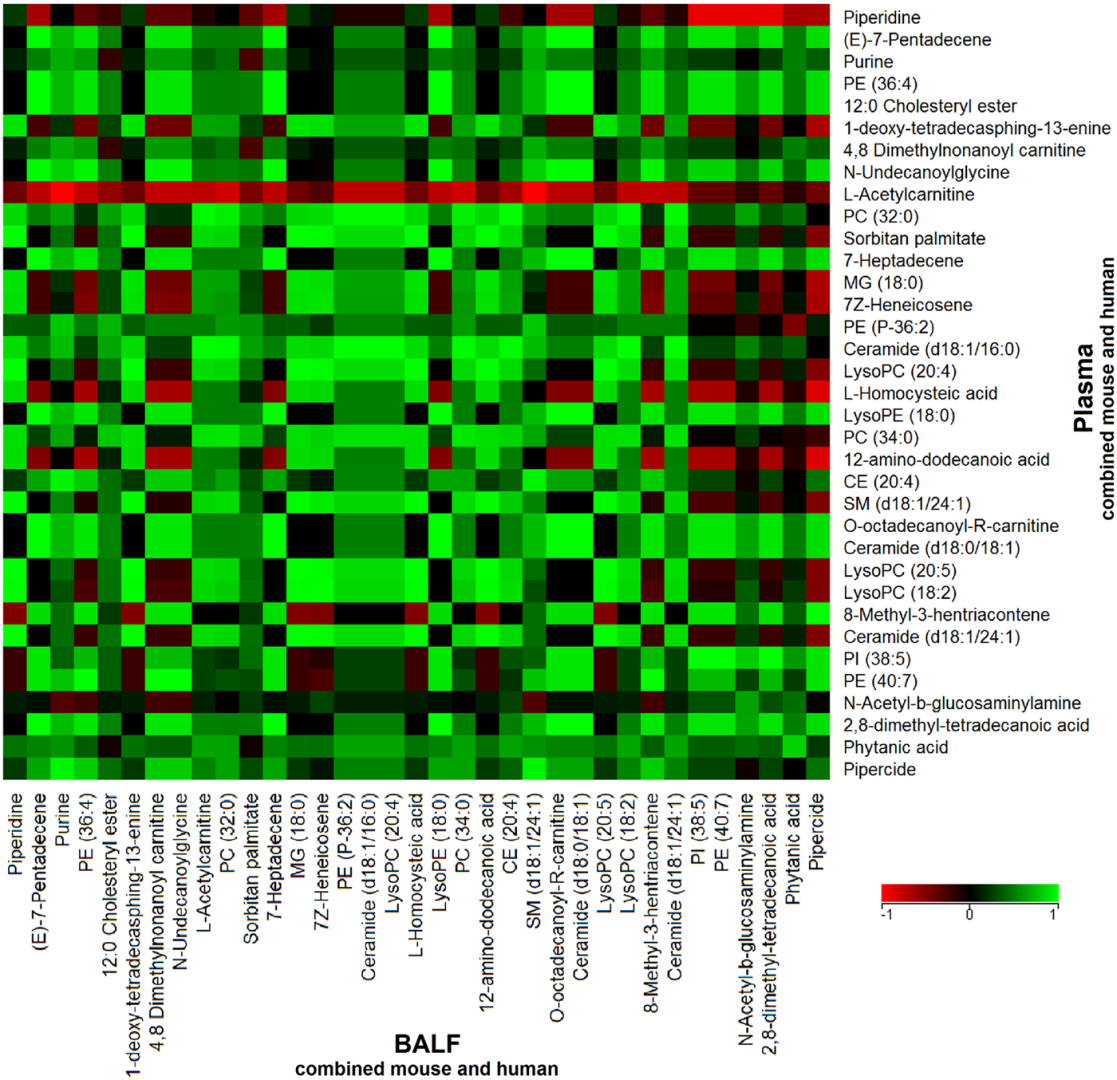
Metabolite correlations across BALF and plasma. Data from the mouse and human BALF and plasma samples were combined to identify metabolites that correlated across both biofluids using Spearman rank correlation. Out of 298 annotated metabolites, a subset of 35 metabolites was selected based on their detected high abundances in BALF and plasma. Red indicates a negative correlation (r = −1) across BALF and plasma, green indicates a positive correlation (r =+1), and black indicates no correlation (r = 0) between BALF and plasma.

We next focused on individual metabolites that may correlate in BALF and plasma, irrespective of species. Out of 298 annotated metabolites, about half were positively correlated. Figure 2 shows the correlation plot of a subset of these metabolites that had high abundance across all the sample types and were diverse across metabolite class. L-acetylcarnitine was the only negatively correlated metabolite across BALF and plasma. In addition, BALF acetylcarntine positively correlated with nine plasma metabolites, while plasma acetylcarnitine negatively correlated with 18 BALF metabolites. Twenty-four other metabolites in this subset were positively correlated in BALF and plasma. These included L-homocysteic acid, octadecanoyl-carnitine, N-undecanoylglycine, LysoPE(18:0), LysoPC(20:4), MG(18:0), PC(32:0), PC(34:0), PE(40:7), and PI(38:5).

We then compared the correlation of individual molecules across the biofluids. In plasma, MG(18:0), ceramide(d18:1/16:0), PC(32:0), PC(34:0), and LysoPC(20:4) correlated positively with LysoPC(20:5) and LysoPC(18:2) in BALF. There was a positive correlation between BALF 12-amino-dodecanoic acid and plasma ceramide(d18:1/16:0). There was no correlation between BALF SM(d18:1/24:1) and plasma homocysteic acid.

### Unique metabolites across BALF and plasma in mice and humans

While plasma metabolites can conceivably be used as proxies for lung metabolites, it is also important to determine what compounds are unique to each biofluid and species. Therefore, we determined the compounds that were only present in a single sample type. Unique metabolites were detected in each of the four sample groups; 506 in mouse BALF, 603 in human BALF, 960 in mouse plasma, and 1,478 in human plasma (Fig. 1A). Therefore, 33.9% of the human plasma metabolites were only found in human plasma. Similarly, 22.2% of mouse plasma metabolites were only found in mouse plasma, 18.4% in only human BALF, and 16.7% in only mouse BALF. Table 2 shows a list of unique annotated metabolites detected in each group.

**Table 2:** Representative unique metabolites to mouse and human BALF and plasma.

Compound	Biofluid	Type	Formula	Accession ID
3-keto Fusidic acid	Human BALF	Drug (fusidic acid) metabolite	C31 H46 O7	HMDB60745
Epi-coprostanol	Human BALF	Endogenous	C27 H48 O	HMDB01569
N-Formyl-L-methionine	Human BALF	Endogenous	C6 H11 N O3 S	HMDB01015
N-hexadecanoyl-leucine	Human BALF	Endogenous	C22 H43 N O3	LMFA08020115
P1,P4-Bis(5′-xanthosyl) tetraphosphate	Human BALF	Endogenous	C20 H26 N8 O23 P4	HMDB03834
PE(35:0)	Human BALF	Odd chain lipid	C40 H80 N O8 P	HMDB08899
Sphingosine-1-phosphate (d19:1-P)	Human BALF	Odd chain lipid	C19 H42 N O5 P	HMDB60062
Uridine diphosphate acetylgalactosamine 4-sulfate	Human BALF	Endogenous	C17 H27 N3 O20 P2 S	HMDB00934
8-Hydroxycarteolol	Human Plasma	Drug (carteolol) metabolite	C16 H24 N2 O4	HMDB60990
DG(29:1)	Human Plasma	Odd chain lipid	C32 H60 O5	HMDB55987
DG(33:4)	Human Plasma	Odd chain lipid	C36 H62 O5	HMDB07329
Hydroxybupropion	Human Plasma	Endogenous	C13 H18 Cl N O2	HMDB12235
N-hexadecanoyl-glutamic acid	Human Plasma	Endogenous	C21 H39 N O5	LMFA08020087
Piperine	Human Plasma	Food	C17 H19 N O3	HMDB29377
Stearoylethanolamide	Human Plasma	Endogenous	C20 H41 N O2	HMDB13078
Valeracetate	Human Plasma	Food	C17 H28 O3	HMDB41388
N-hexadecanoyl-valine	Mouse BALF	Endogenous	C21 H41 N O3	LMFA08020120
Urocanic acid	Mouse BALF	Endogenous	C6 H6 N2 O2	HMDB00301
Endomorphin-1	Mouse Plasma	Endogenous	C34 H38 N6 O5	HMDB05773
N-Nonanoylglycine	Mouse Plasma	Endogenous	C11 H21 N O3	HMDB13279
PIP(38:3)	Mouse Plasma	Endogenous	C47 H86 O16 P2	HMDB09989
Pristanal	Mouse Plasma	Endogenous	C19 H38 O	HMDB01958
PE(39:1)	Mouse Plasma	Odd chain lipid	C44 H86 N O8 P	HMDB09747
PS(37:5)	Mouse Plasma	Odd chain lipid	C43 H74 N O10 P	LMGP03010599

This list contains randomly selected metabolites that were annotated using an in-house database comprised of METLIN, HMDB, Lipid Maps and KEGG. Metabolites were selected randomly. Scores ≥70 out of a possible 100 and mass errors ≤10 ppm were used for annotation thresholds. Annotations were based on exact mass and isotope ratios. DG: diglyceride, PE: phosphatidylethanolamine, PIP: phosphatidylinositol phosphate, PS: phosphatidylserine.

### Distribution of compound classes across sample types

**Figure 3:**
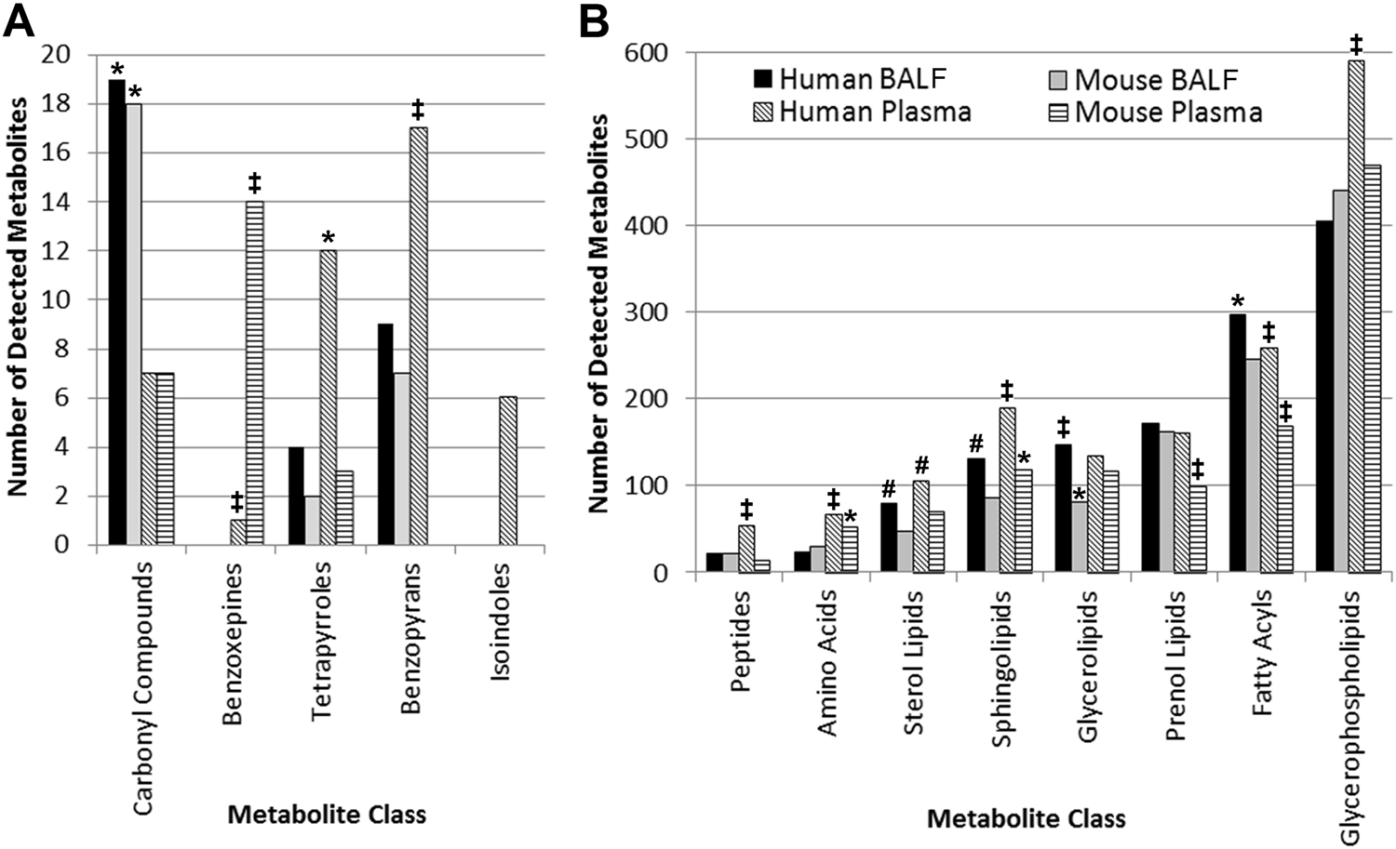
Metabolite coverage based on compound class for BALF and plasma in mouse and human samples. Indicated classes passed a proportional test used to analyze the metabolites in each of the four indicated groups; *p < 0.05; ^#^p < 0.01; ^‡^p < 0.001. Metabolite class categories were determined using the Human Metabolome Database (HMDB) and Lipid Maps classifications. (**A**) Metabolite classes with less than 20 detected metabolites. (**B**) Metabolite classes with >20–600 detected metabolites.

Next, we determined whether distinct classes of compounds were found predominantly in any biofluid. Sixty compound classes were tested using a proportional test (described in methods); thirteen had significant differences for the proportion of compounds detected in the class across the groups (Fig. 3). The most represented metabolite classes common to all of the four groups were prenol lipids, fatty acyls, and glycerophospholipids (Fig. 3B). The prenol lipids range from quinones, hydroxyquinones, C20 isoprenoids, and retinoids, to triterpenoids and terpene glycosides. A few examples from these prenol lipid classes include coenzymes, vitamins such as A, E and K, retinoic acid as well as plant-related metabolites in plasma such as acetylursolic acid. The fatty acyls include octadecanoids and fatty acyl glycosides. The glycerophospholipids include phosphatidylethanolamines (PE), phosphatidylcholines (PC), phosphatidylserines (PS), phosphatidylglycerols (PG), and phosphatidylinositols (PI). Due to the enrichment of lipids from the BALF and plasma during sample preparation, and the optimization of the LC-MS method to detect and separate lipids, a large number of lipid species were identified. Figure 3A shows that benzopyrans, peptides, amino acids, sterol lipids, sphingolipids, and glycerophospholipids were highly represented (p < 0.001) in human plasma compared to the other sample groups. Isoindoles were only detected in human plasma. Carbonyl compounds, glycerolipids, and fatty acyls were highly represented (p < 0.05) in human BALF. Benzopyrans were present in human BALF, mouse BALF, and human plasma; however, these were absent in mouse plasma (Fig. 3A). Benzoxepines were highly represented in mouse plasma (p < 0.001); however they were absent in human BALF and mouse BALF.

### Cigarette smoke induced metabolome changes in BALF and plasma

**Figure 4:**
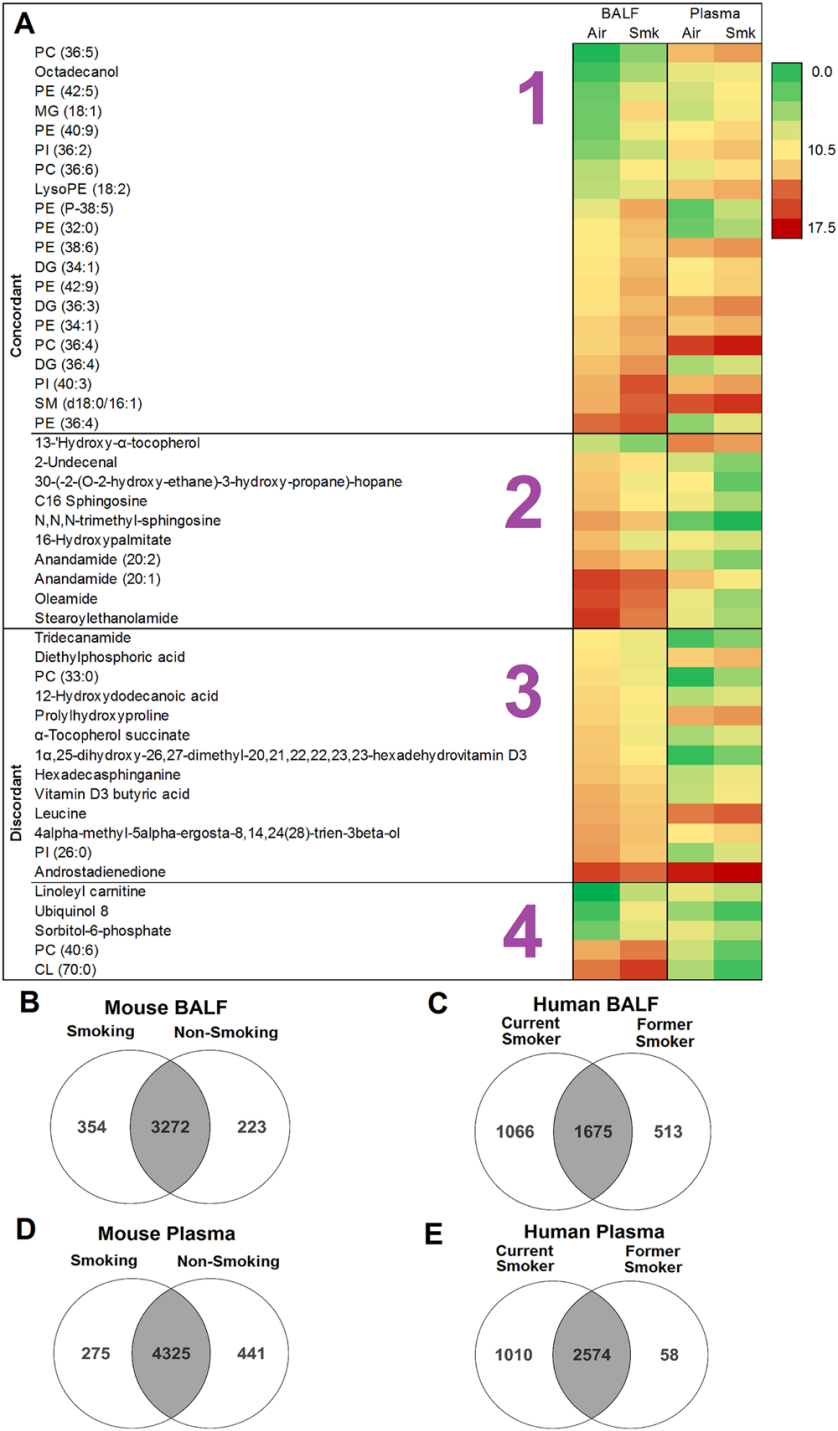
Metabolome changes in response to cigarette smoke. (**A**) Heat map of statistically significant and differentially regulated metabolites in mouse BALF and plasma in response to cigarette smoke compared to air control groups (n = 7/group). Metabolite abundances range from 0 (green) to 17.5 (red). Statistical analysis was performed in Mass Profiler Professional 13.1 (Agilent) using Storey with Bootstrapping q ≤ 0.1 and fold change ≥1.5. CL: cardiolipin, PE: phosphatidylethanolamine, PC: phosphatidylcholine, PI: phosphatidylinositol, SM: sphingomyelin, MG: monoglyceride, DG: diglyceride. The four sections of the heat map are as follows: 1 – metabolites are up-regulated in both BALF and plasma, 2 – metabolites are down-regulated in both BALF and plasma, 3 – metabolites are down-regulated in BALF and up-regulated in plasma, 4 – metabolites are up-regulated in BALF and down-regulated in plasma in response to CS-exposure. (**B**) Overlap of metabolites in BALF from smoking and non-smoking mice. **(C)** Overlap of metabolites in human BALF from current and former smokers. (**D**) Overlap of metabolites in plasma from smoking and non-smoking mice. (**E**) Overlap of metabolites in human plasma from current and former smokers.

The BALF and plasma metabolomes were compared in a group of mice exposed to ambient air or cigarette smoke for 1 day (n = 7 mice per group), to determine the congruence of metabolite changes due to acute CS exposure. There were 124 plasma metabolites and 380 BALF metabolites that were differentially regulated and database annotated in smoking versus non-smoking mice (Storey with Bootstrapping multiple testing correction, q < 0.1); 48 of these differentially regulated metabolites were common to both groups. Their degree of congruence is presented as a heat map in Fig. 4A. There were 30 compounds with the same direction of regulation (concordant) in both BALF and plasma and 18 metabolites with opposite directions (discordant) in BALF and plasma. Overall, the following changes were observed in response to smoking: glycerophospholipids and glycerolipids were up-regulated in BALF and plasma. Two anandamides and two sphingolipids were down-regulated in both BALF and plasma. Leucine, two steroids and two vitamin D3 metabolites were down-regulated in BALF but up-regulated in plasma. Ubiquinol-8 and linoleyl carnitine were up-regulated in BALF and down-regulated in plasma.

We then explored the effect of smoking on the global BALF and plasma human metabolome (Fig. 4B–E). Due to the small sample size, only qualitative analysis could be conducted. In summary, it appears that current cigarette smoking results in additional metabolites compared to the former or non-smoking groups. For example, there were 3626 compounds in the current smokers versus 3495 in the non-smokers in mouse BALF; there were 2741 compounds in the current smokers versus 2188 in the former smokers in human BALF; there were 3584 compounds in the current smokers versus 2632 in the former smokers in human plasma.

## Discussion

This study used LC/MS-based metabolomics to catalogue compounds in mouse BALF, mouse plasma, human BALF, and human plasma. These compounds were compared to determine overlap amongst the groups and to identify concordant and discordant changes in BALF and plasma in a mouse model of CS exposure. Overall, we found that over 50% of metabolites were common to all four sample groups. Lipids were more prevalent compared to aqueous small molecules such as amino acids and purines; this could be due to sensitivity limitations. A recent study by Peng *et al*. detected 250 aqueous compounds in rat BALF^16^. This is consistent with the 275 and 331 aqueous molecules detected in our aqueous fraction of the human BALF and mouse BALF, respectively. Their study, like ours, identified metabolites belonging to amino acid and purine metabolite classes; these findings are consistent with other studies that analyzed human BALF^17^.

Some of the compounds detected in both BALF and plasma in our study include acetylcarnitine, carnitine, creatine, MG(18:0), leucine, and hypoxanthine. Dysregulations of these metabolites have previously been reported in the BALF of mice^12^, rats^16^, and humans^17^ in association with asthma, COPD^13^ and/or acute respiratory distress syndrome (ARDS). The presence of these molecules in both BALF and plasma suggests that plasma could be used as a surrogate for BALF, thereby providing a non-invasive fluid to study these lung diseases. Their dysregulation in both mice and humans also suggests that mice may be useful models in studying human lung disease including emphysema, as demonstrated in this cigarette smoking model.

Signaling molecules such as LysoPCs, ceramides, and diglycerides were common to mouse and human plasma and are associated with dysregulated plasma levels in airway diseases such as asthma^18–20^, in human COPD plasma^8^, and upon exposure to CS in an animal model^7^. In addition, amino acids, sphingolipids, and vitamin D are associated with lung diseases. Some examples include: serum and plasma vitamin D deficiency in asthma^21, 22^, serum amino acids perturbation in COPD^23^, plasma and CSF amino acids perturbation in smokers^24^, and an increase in lung tissue sphingolipids in cystic fibrosis^25^. Collectively, results suggest that metabolites are conserved across species and biological fluids. Additional studies in larger, disease-specific cohorts are necessary to understand the roles of these common compounds in disease and to determine if plasma metabolites can act as non-invasive surrogates for lung tissue or BALF metabolites.

Next, we determined the correlation in metabolites between species and biofluid. We observed that mouse BALF and human BALF were positively correlated. BALF metabolites reflect the lining of the airways; therefore, it was not surprising that BALF in mice and humans were the most similar. Mouse plasma and mouse BALF were the next positively correlated. This may be due to the controlled environment of the mice, including identical feeding and cage conditions. The presence of large numbers of exogenous metabolites in human plasma may also explain why human plasma did not correlate strongly with any of the other tested fluids. In spite of this, several metabolites were correlated between BALF and plasma, although these findings require validation in larger cohorts.

We then explored unique metabolites from each sample group. Many of the unique metabolites (MS level 2 putative identifications) in the human plasma may be attributed to the contribution of exogenous metabolites from diet, xenobiotics, medications, and environmental exposures^26^ compared to controlled mouse studies. For example, 8-hydroxycarteolol was only detected in the human plasma; 8-hydroxycarteolol is a metabolite of the drug carteolol. 3-keto fusidic acid is a metabolite of the antibiotic fusidic acid and was only detected in human BALF. Food metabolites such as valeracetate and piperine were unique to the human plasma. Another example, Δ9-tetrahydrocannabinol (a cannabis metabolite) was detected in the human BALF and human plasma but not in the mouse samples. Odd chain lipids were uniquely detected in either human BALF [*PE(35:0), sphingosine-1-phosphate (d19:1-P)*], human plasma [*DG(29:1), DG(33:4)*], or mouse plasma [*PE(39:1), PS(37:5)*]. Odd chain lipids have historically been suggested to be bacterial in origin; however, recent studies have noted their presence in plant and mammalian species^27^. These odd chain lipids are associated with disease including cardiovascular and peroxisomal disorders^28^. Exposure to CS may potentially influence levels of certain endogenous metabolites. Alternatively, cigarette smoke is known to cause adduction/modification; although, to our knowledge, this has not been widely reported for small molecules. Overall, the large number of unique metabolites could be explained by both endogenous and exogenous components.

In many disease conditions, changes are seen in a compound class rather than a single molecule. Therefore, the distribution of metabolite classes was examined across the four sample groups. The lipid classes such as glycerophospholipids were most highly represented in all sample types. This is expected as glycerophospholipids are a major component of cellular membranes^29^. The abundance of lipids in the human plasma is consistent with previous studies of human plasma^30^. Many compounds in these lipid classes play crucial roles in disease and inflammation^31–36^. Sphingolipids, for example, are associated with CS-induced injury and COPD^8, 37, 38^. Benzopyrans and isoindoles were predominant in human plasma. Benzopyrans exhibit anti-inflammatory properties through inhibition of prostaglandin E_2_ production. Isoindoles are natural products with diverse biological activities including anticancer or antimicrobial properties^39^. Based on their presence in both plasma and BALF and their relationship to disease, these molecules are potentially good proxy candidates.

We then compared the global metabolite profile of human and mouse, plasma and BALF following CS exposure. Qualitatively, results showed that the BALF and plasma of current cigarette smokers contained more metabolites than the former or non-smoker. In addition, the presence of unique metabolites in the smoking groups of mouse BALF, human BALF, and human plasma also points to the introduction of exogenous metabolites to these metabolomes, potentially due to cigarette additives. Results also suggest that smoking may deplete certain metabolites while enhancing others. When considering cigarette smoke, it is possible that many of the 599 additives in cigarettes and 4,000 chemical compounds in tobacco smoke^40, 41^ may have contributed to the BALF and plasma metabolomes.

Lastly, we investigated changes in the metabolome due to CS exposure in matched mouse biofluids to determine whether plasma reflects changes occurring in the lung. Three times as many changes were observed in BALF than in plasma; this is expected since BALF is closer to the point of injury (i.e. the lung). Many of these metabolite changes were common to both BALF and plasma; 30 compounds were up-regulated in both biofluids, suggesting that these compounds may be of interest to investigators analyzing plasma as a less-invasive means to study the lung. This would be particularly important in emphysema and/or CS-exposure studies, where BALF is difficult to obtain. We observed that sphingolipids were dysregulated in both biofluids: SM(d18:0/16:1) was up-regulated in both BALF and plasma, while C16 sphingosine and N,N,N-trimethyl-sphingosine were down-regulated in both BALF and plasma. Sphingolipids are messenger molecules involved in cellular homeostasis, oxidative stress, and apoptosis. We have previously shown a role for sphingolipids in association with CS exposure, COPD, and emphysema^8, 38, 42^. These compounds as well as the strongly positively correlated metabolites, have a dual purpose: (1) those present in both BALF and plasma offer a non-invasive clinical alternative to collecting plasma instead of BALF in humans, (2) those present in mice and humans are important in translational studies, such as in drug trials, or for preliminary studies in mice with the goal of subsequent studies in humans.

There were 18 compounds with an opposing direction of regulation in BALF vs. plasma. Linoleyl carnitine was up-regulated in plasma and down-regulated in BALF. Carnitines have not been widely reported in association with lung and airways disease or cigarette smoke exposure. However, L-carnitine has been shown to improve symptoms in children with moderate persistent asthma when administered orally^43^. Dietary supplementation of L-carnitine has also been shown to reverse renal oxidative stress and mitochondrial dysfunction in female BALB/c mice who were exposed to cigarette smoke^44^. In a recent study, L-carnitine decreased with emphysema progression in mice, and L-carnitine supplementation improved lung function and reduced apoptosis^13^. Two vitamin D3 metabolites were down-regulated in BALF but up-regulated in plasma. Vitamin D deficiency has been reported in response to CS exposure, and in asthmatic and COPD patients^45, 46^. Our results suggest active transport across the lung/blood barrier, potentially explaining their decrease in BALF and increase in plasma.

We acknowledge that limitations exist in our study. Metabolite annotations were based on exact mass and isotope ratios; only a selected number of metabolite annotations were confirmed using MS/MS since obtaining authentic standards and MS/MS was not possible for thousands of metabolites. However, since identical conditions were used, including sample preparation and chromatography, annotations are consistent and comparable across samples. Also, the sample size for the human cohort was small and as such, statistical comparisons could not be performed. Future work will focus on addressing these limitations.

## Conclusion

Over 50% of metabolites overlap between plasma and BALF of mice and humans. Metabolites in common between species are good candidates for molecular intervention studies in mouse models. CS exposure studies revealed that although certain metabolites were concordant between BALF and plasma, others exhibited opposing directions. This emphasizes the biological complexity in studying whole organisms and potential of a system to compensate for changes due to external or internal stimuli. Results from the mice suggest that CS-induced changes in the lung may not be fully recapitulated in plasma; further, interrogation of one biofluid may not be sufficient to inform on health status. Since sample size was limited, further experiments are required to arrive at specific conclusions regarding biological perturbations. However, overall, our findings support the use of mouse models and plasma as proxies for human samples when studying lung disease.

## Methods

### Ethics statement

All methods were performed in accordance with the relevant guidelines and regulations. Animal studies were approved by the Animal Care and Use Committee of Indiana University. Human subjects were from the Genetic Epidemiology of COPD (COPDGene) cohort, which is a National Institutes of Health–sponsored multicenter study of the genetic epidemiology of COPD^47^. COPDGene was approved by the institutional review board at each participating center; all subjects were enrolled from January 2008 to April 2011 and provided written informed consent. The current analysis was approved by the National Jewish Health Institutional Review Board.

### Animal studies

For the metabolite catalogue analysis, matched plasma and BALF was collected from C57BL/6 mice (Jackson Laboratory, Bay Harbor, ME). Three-month old female mice were exposed to ambient air for one day (n = 5 air control) and mice were exposed to CS for up to nine months (n = 5 smoking). For the statistical comparisons used to determine congruence between BALF and plasma upon acute CS exposure, mice were exposed to ambient air for one day (n = 7 air control) or exposed to cigarette smoke for one day (n = 7 smoking).

The acute exposure mice were exposed for 5 hours per day, while the chronic exposed mice were exposed for 5 hours per day, 5 days a week to 11% mainstream and 89% side stream smoke from reference cigarettes (3R4F; Tobacco Research Institute, Kentucky) using a Teague 10E whole body exposure apparatus (Teague Enterprise, CA) with monitored suspended particulates (average 90 mg/m^3^) and carbon monoxide (average 350 ppm). At the end of experiments, the mice were euthanized. The pathophysiologic features between the air control and smoking mice in this CS model have been previously published^7, 48–50^.

Blood was collected via venipuncture of the right ventricle and collected in tubes with 1X Complete EDTA-free protease inhibitors (Roche). Plasma was isolated, snap frozen and stored at −80 °C until analysis. BALF collection was performed using a total of 1.0 mL PBS divided into three washes. The first wash was spun down and the supernatant (acellular BALF) was used for analysis.

### Human studies

Human subjects were from the Genetic Epidemiology of COPD (COPDGene) cohort^47^. Matched human plasma and BALF was collected from a small subset of subjects of which BALF was also available (n = 5). COPD diagnosis was based on ratio of forced expiratory volume in 1 second to forced vital capacity (FEV_1_/FVC). Subjects were 45–70 years old, BMI 27–45, weight 76–125 kg, and were categorized as follows: 1 male former smoker without COPD (FEV_1_/FVC = 0.82), 2 male current smokers without COPD (FEV_1_/FVC = 0.91 and 0.79), 1 female current smoker with moderate COPD (FEV_1_/FVC = 0.51), and 1 male current smoker with moderate COPD (FEV_1_/FVC = 0.64).

Plasma was collected using a P100 tube (BD) as described previously^51^. BALF was obtained as described previously^52^. Briefly, BALF was collected in the right middle lobe and lingual by instilling two aliquots of 40 mL and one aliquot of 50 mL of sterile saline per lobe (i.e., 130 mL per lobe, total volume = 260 mL per subject), which is withdrawn by gentle manual suction and immediately placed on ice. Samples were sub-aliquoted into vials for a variety of studies. Aliquots for metabolomics analysis were frozen at −80 °C and stored until sample preparation and MS analysis.

### Chemicals, standards and reagents

Solvents used for metabolite extraction and LC/MS analysis were of HPLC or LC/MS-grade as follows: water and isopropyl alcohol from Honeywell Burdick & Jackson (Muskegon, Michigan); methyl tert-butyl ether from J.T. Baker (Central City, Pennsylvania); acetonitrile, methanol, chloroform, formic acid, and acetic acid from Fisher Scientific (Fair Lawn, New Jersey); standards from Avanti Polar Lipids Inc. (Alabaster, AL) and Sigma Aldrich (St. Louis, MO); glass pipette tips, plastic pipette tips, and microcentrifuge tubes from Fisher Scientific (Fair Lawn, New Jersey); Pyrex glass culture tubes from Corning Incorporated (Corning, New York).

### Sample preparation for BALF and plasma

BALF and plasma samples were stored at −80 °C prior to sample preparation. Protein precipitation using methanol, and liquid-liquid extraction using methyl-tert butyl ether (MTBE) was performed on 100 µL of BALF and plasma as previously described^14, 53^. An aqueous fraction and a lipid fraction were obtained. Plasma and BALF lipids were reconstituted in 200 µL of methanol; plasma aqueous metabolites were reconstituted in 100 µL of 95:5 water:acetonitrile. Due to low concentrations of aqueous metabolites, 200 µL of BALF was used and the aqueous fraction was dried down in a speedvac at 45 °C and reconstituted in 50 µL of 95:5 water:acetonitrile.

### Liquid chromatography

Lipid fractions of extracted BALF and plasma samples were resolved using reverse phase chromatography using an Agilent Zorbax Rapid Resolution HD (RRHD) SB-C18, 1.8 micron (2.1 × 100 mm) analytical column and an Agilent Zorbax SB-C18, 1.8 micron (2.1 × 5 mm) guard column. An Agilent 1290 series high performance liquid chromatography (HPLC) pump was used. Injection volumes were adjusted because of sample dilution effects in BALF (our preliminary sample extraction studies showed human BALF was at least four times more diluted than mouse BALF). These dilution differences in sample types were adjusted as follows: 4 µL of mouse or human plasma were injected, 4 µL mouse BALF was injected, and 15 µL human BALF was injected. HPLC flow rate was 0.7 mL/min with the following mobile phases: mobile phase A was water with 0.1% formic acid, and mobile phase B was 60:36:4 isopropyl alcohol:acetonitrile:water with 0.1% formic acid. The gradient was as follows for positive mode: 0–0.5 minutes 30–70% B, 0.5–7.42 minutes 70–100% B, 7.42–9.9 minutes 100% B, 9.9–10.0 minutes 100–30% B, 10–14.6 minutes 30% B. Autosampler tray temperature was set to 4 °C and column temperature was set to 60 °C. The gradient was as follows for negative mode: 0–1 minutes 30–70% B, 1–7.92 minutes 70–100% B, 7.92–10.4 minutes 100% B, 10.4–10.5 minutes 100–30% B, 10.5–15.1 minutes 30% B. Autosampler tray temperature was set to 4 °C and column temperature was set to 60 °C.

Normal-phase chromatography was used to analyze the aqueous fraction of the mouse and human plasma samples on an Agilent 1200 series pump using a Phenomenex Kinetex HILIC, 2.6 µm, 100 Å (2.1 × 50mm) analytical column and an Agilent Zorbax Eclipse Plus-C8 5 µm (2.1 × 12.5 mm) narrow bore guard column. For all sample types, 1 µL was injected with a flow rate of 0.6 mL/min. Mobile phase A was 50% ACN with pH 5.8 ammonium acetate, and mobile phase B was 90% ACN with pH 5.8 ammonium acetate. Gradient elution was as follows: 0–2 minutes 100% B, 2–2.1 minutes 100–90% B, 2.1–8.6 minutes 90–50% B, 8.6–8.7 minutes 50–0% B, 8.7–14.7 minutes 0% B, 14.7–14.8 minutes 0–100% B, 14.8–24.8 minutes 100% B. Autosampler tray temperature was set to 4 °C and column temperature was set to 20 °C.

Reversed-phase chromatography was used to analyze the aqueous fraction of the mouse and human BALF samples on an Agilent 1200 series pump using an Agilent Zorbax Narrow Bore RRHT SB-AQ (1.8 micron, 2.1 × 100 mm, 80 Å) analytical column and an Agilent Zorbax SB-AQ (5 micron, 2.1 × 12.5 mm) guard column with a 10 µL sample injection volume. The flow rate was 0.3 ml/min using the following mobile phases: mobile phase A was water with 0.1% formic acid, and mobile phase B was 90:10 acetonitrile:water with 0.1% formic acid. Gradient elution was as follows: 0–3 minutes 2% B, 3–5 minutes 2–40% B, 5–20 minutes 40–100% B, 20–30 minutes 100% B, 30–30.01 100–2% B, 30.01–40 minutes 2% B. Autosampler tray temperature was set to 4 °C and column temperature was set to 30 °C.

### Mass spectrometry (MS)

The lipid fraction positive mode MS conditions for the BALF and plasma samples were as follows: Agilent 6210 Time-of-Flight (TOF-MS) with dual ESI source, scan rate 2.03 spectra/second, mass range 60–1600 m/z, gas temperature 300 °C, gas flow 12.0 L/min, nebulizer 30 psi, skimmer 60 V, capillary voltage 4000 V, fragmentor 120 V, reference masses 121.050873 and 922.009798 (Agilent reference mix). The negative mode conditions were as follows: Agilent 6210 Time-of-Flight (TOF-MS) with dual ESI source, scan rate 2.02 spectra/second, mass range 60–1600 m/z, gas temperature 300 °C, gas flow 12.0 L/min, nebulizer 30 psi, skimmer 60 V, capillary voltage 4000 V, fragmentor 140 V, reference masses 112.985628 and 966.000725 (Agilent reference mix).

The aqueous fraction MS conditions for the BALF and plasma samples were as follows: Agilent 6520 Quadrupole Time-of-Flight (Q-TOF-MS) in positive ionization mode with ESI source, mass range 50–1700 m/z, scan rate 2.22 spectra/second, gas temperature 300 °C, gas flow 10.0 L/min, nebulizer 30 psi, skimmer 60 V, capillary voltage 4000 V, fragmentor 120 V, reference masses 121.050873 and 922.009798 (Agilent reference mix).

### Quality control (QC)

To limit variations in metabolite abundances, sensitivity, and batch effects, all samples were prepared on the same day. Also, samples were analyzed in a single LC/MS run to avoid batch effects to avoid day-to-day variation, HPLC column changes, or instrument drift. Total ion chromatograms (TIC) were evaluated for retention time reproducibility using spiked internal standards and endogenous compounds. The largest retention time variation was 0.58% and 1.54% for the spiked standards and endogenous compounds respectively and represents a variation <0.25 minutes, which is well within acceptable limits. Signal intensity of the TICs was also evaluated. The largest variation was less than 10% CV in the largest range of the TIC, and HPLC pressure curves were less than 5% CV. Instrument QC samples, injected after every five samples, were analyzed to ensure that peak areas of 9 spiked internal standards were reproducible (<10% CV) throughout the analysis. The % CVs for the internal standards in the aqueous plasma analysis and aqueous BALF analysis was less than 10%, and for the BALF and plasma samples in the lipid analysis was less than 5%. The % CVs, retention times, and peak areas for the internal standards and selected endogenous compounds are presented in Supplemental Table S1. These standards were used for quality control purposes rather than for normalization.

### Data processing

Spectral data was extracted using the following parameters in MassHunter software (Agilent Technologies): Find by Molecular Feature algorithm, single charge, proton, sodium, potassium, ammonium adducts in positive ionization mode. Data were imported into Mass Profiler Professional software (MPP, Agilent Technologies) for mass (15 ppm) and retention time alignment (0.2 minutes), and data filtered by selecting features that were present in at least 50% of each sample group. Data from sample preparation blanks and instrument blanks were background subtracted to eliminate noise from contaminants. Because LCMS data can result in missing values^54^, data was further processed using the ‘Find by Formula’ algorithm parameters (+H, +Na, +K, +NH_4_ adducts for positive ionization mode, charge states limited to 2, and absolute height >3000 counts). The ‘Find by Formula’ algorithm merged multiple features such as ions, adducts and dimers into a single compound which resulted in 7654 total compounds in all sample types and in both species (BALF lipid+, BALF lipid−, BALF aqueous, plasma lipid+, plasma lipid−, plasma aqueous). The final data set was then re-imported into MPP for differential and statistical analysis. Compounds were compared using several strategies across the samples (human BALF, human plasma, mouse BALF, mouse plasma), fractions (lipid versus aqueous), and ionization mode (positive and negative). The metabolites and their associated signal values were exported to GraphPad Prism v6.04 and Excel Professional Plus 2010 (Microsoft Corporation, Redmond, WA) for visualization purposes.

The total volume of compounds (*number of compounds and peak area of each*) in the individual samples was calculated using MassHunter Profinder (Agilent). BALF data was normalized to total volume using external scalar. This external scalar normalization technique used total volume to reduce the variance in the biological measurements due to dilution effects in BALF from sample collection^55^. Variability was evaluated using coefficient of variation^56^. Metabolites with <10% CV increased from 209 without normalization to 1192 post-normalization in the control mice, and increased from 219 to 1191 metabolites in the smoking mice post-normalization.

### Metabolite annotation

ID Browser within the Mass Profiler Professional (MPP) software v13.1 (Agilent) was used to tentatively annotate metabolites. This software utilizes an in-house database comprising data from METabolite LINk (METLIN), Human Metabolome Database (HMDB), Kyoto Encyclopedia of Genes and Genomes (KEGG), and Lipid Maps; MPP uses isotope ratios, accurate mass, chemical formulas, and database scores (scale of 0 to 100) to annotate compounds by database ID, molecular formula, or compound number. A database score >70 out of a possible 100 was considered acceptable for annotation confidence; results were manually confirmed. Molecular formula generation included the following elements: C, H, N, O, S, and P. An error window of <10 ppm was used with a neutral mass range up to 2000 Da. The database identifications were limited to the top 10 best matches based on score, and charge state was limited to a maximum of 2. Tandem MS was used to improve confidence in identifications based on fragmentation information. Fragments were matched to reference standards from METLIN and NIST14 MSMS spectral libraries^57^. All identifications are Metabolomics Standards Initiative (MSI) level 2 based on the proposed minimum reporting by Sumner^58^.

Annotated metabolites were grouped into classes using the Human Metabolome Database (HMDB) and Lipid Maps classification system. For the compound classes with four or less detected metabolites in at least one of the four groups (human BALF, human plasma, mouse BALF, mouse plasma), that class was excluded for at least 2 of the following reasons: (1) most likely a false annotation, (2) below the detection level of the instrumentation, or (3) too many classes to display due to space limitations.

### MS/MS analysis

The HILIC, C18 and SB-AQ chromatographic methods were replicated for LC-MS/MS analysis using 10, 20, and 40 eV collision energies on a 6520 Q-TOF (Agilent) with a 500 ms/spectra acquisition time, 4 m/z isolation width, and 1 minute delta retention time.

Fragmentation data was exported to the freely available NIST MS Search v.2.2 g GUI program^59^ (NIST, Gaithersburg, MD, USA) and were matched to spectra in the NIST 14 Mass Spectral Library. This library contains 193,119 spectra representing 43,912 precursor ions and 8,351 compounds; a detailed description of the library is available^60^. Automated library searching was performed using spectrum search type ‘Identity’, search with “MS/MS”, and default program settings. The search *m/z* tolerance was ±0.4 for precursor ions and ±0.4 for product ions without ignoring the precursor ion. The MS search program outputted a list of matched chemical compounds including several measures of spectral similarity^61^. The Match Factor (MF) is the normalized dot product with square-root scaling of the experimental mass spectrum and a library mass spectrum, using all the elements in the experimental mass spectrum. The Reverse Match Factor (RMF) is the normalized dot product with square-root scaling of the experimental mass spectrum and the library mass spectrum, but the elements that are not present in the library mass spectrum are not included.

Fragments were matched to reference standards from METLIN and NIST14 MSMS spectral libraries^57^. Selected matches are presented in Supplemental Tables S2 and S3.

### Statistical analysis

#### Metabolite class testing

Analysis of sixty metabolite classes was performed in R using a proportional test^62^ to test whether the proportion of metabolites detected (out of all metabolites defined for that class, categorized by Lipid Maps and HMDB) was different among the groups (p < 0.05). Subsequent analysis was performed to determine which of the groups was significant within each of the significant classes.

#### Correlation analysis

Spearman’s rank correlation coefficient was used for correlation calculations, and coefficients were tested if they were significantly different from 0 in R. Significance was considered at p < 0.05.

#### Differential analysis across mouse BALF and plasma

Statistical analysis of the matched mouse BALF and plasma samples was performed using MPP v13.1 (Agilent). An unpaired t-test was used to compare matching BALF and plasma for day 1 air controls (n = 7) and day 1 cigarette smoking mice (n = 7). Metabolites that were present in at least 50% of each group, passed fold change ≥±1.5, and Storey with Bootstrapping multiple testing correction q ≤ 0.1 are reported. Because the sample size for human BALF and plasma was small (n = 5), statistical comparison between smoking and non-smoking humans was not possible for this dataset. Excel Professional Plus 2010 (Microsoft Corporation, Redmond, WA) was used to create graphics.

## Electronic supplementary material

Dataset 1
