# Supplementary material for: Metabolomic similarities between bronchoalveolar lavage fluid and plasma in humans and mice
Source: Sci Rep. 2017 Jul 11;7:5108. doi: 10.1038/s41598-017-05374-1 (PMC5505974; doi:10.1038/s41598-017-05374-1)
Supplement: Supplementary file 1 — Dataset 1 [file 41598_2017_5374_MOESM1_ESM.doc]

**Metabolomic similarities between bronchoalveolar lavage fluid and plasma in humans and mice**

**Charmion Cruickshank-Quinn^1^, Roger Powell^1^, Sean Jacobson^3^, Katerina Kechris^2^, Russell P. Bowler^3^, Irina Petrache^3^, Nichole Reisdorph^1^***

^1^Department of Pharmaceutical Sciences, University of Colorado Denver|Anschutz Medical Campus, Aurora, CO 80045

^2^Department of Biostatistics and Informatics, University of Colorado Denver|Anschutz Medical Campus, Aurora, CO 80045

^3^Department of Medicine, National Jewish Health, Denver, CO 80206

Table of Contents

[**Supplemental Table S1**: Quality control results from each analysis 2](#__RefHeading___Toc464913837)

[**Supplemental Table S2**: Fragmentation patterns of selected detected metabolites in the mouse and human samples 3](#__RefHeading___Toc464913838)

[**Supplemental Table S3**: Annotated and matching fragments 20](#__RefHeading___Toc464913839)

[**Supplemental Table S4**: List of selected overlapping annotated metabolites in human and mouse BALF 28](#__RefHeading___Toc464913840)

[**Supplemental Table S5**: List of selected overlapping annotated metabolites in human and mouse plasma 41](#__RefHeading___Toc464913841)

*The Table of Contents is interactive. Click on a Supplemental Table to navigate to the indicated document.*

# Supplemental Table S1: Quality control results from each analysis

| **Compound** | **Type** | **Retention Time & %CV** | | **Abundance & %CV** | |
| --- | --- | --- | --- | --- | --- |
| Creatinine-d3 | Internal Standard | 2.103 min | 0.32% | 802381 | 2.10% |
| PC (17:0/17:0) | Internal Standard | 5.517 min | 0.58% | 102573 | 5.52% |
| Valine-d8 | Internal Standard | 8.502 min | 0.38% | 308845 | 8.50% |
| 3-Dehydroxycarnitine | Endogenous | 8.872 min | 0.15% | 155325 | 3.60% |
| Acetaminophen glucuronide | Endogenous | 1.282 min | 1.11% | 51181 | 5.05% |
| Acetylcarnitine | Endogenous | 6.394 min | 0.15% | 1401887 | 4.65% |
| Apocholic acid | Endogenous | 16.123 min | 0.06% | 272704 | 3.94% |
| Betaine | Endogenous | 3.278 min | 0.42% | 525597 | 6.45% |
| Carnitine | Endogenous | 7.048 min | 0.15% | 4127647 | 3.88% |
| Creatine | Endogenous | 4.615 min | 0.34% | 382746 | 7.98% |
| Isoleucyl-proline | Endogenous | 8.555 min | 0.31% | 122556 | 3.89% |
| Neurine | Endogenous | 5.926 min | 0.50% | 993151 | 2.82% |
| Pyrolidine | Endogenous | 3.277 min | 0.44% | 134321 | 7.46% |
| Taurine | Endogenous | 1.656 min | 1.54% | 72231 | 7.11% |
| Ubiquinol-8 | Endogenous | 17.195 min | 0.25% | 55224 | 6.72% |

**Table S1a: Instrument QC’s for the mouse and human plasma aqueous fraction.** A pooled QC sample was run after every 5 sample injections to monitor instrument conditions. Both lower abundant as well as higher abundant endogenous metabolites have %CV’s lower than 10%. (n= 16 QC injections, 8 per day for 2 days)

| **Compound** | **Type** | **Retention Time & %CV** | | **Abundance & %CV** | |
| --- | --- | --- | --- | --- | --- |
| D-Glucose-13C6 | Internal Standard | 1.029 min | 0.42% | 48843 | 5.80% |
| Testosterone-d2 | Internal Standard | 12.318 min | 0.18% | 65020 | 3.90% |
| 2-(1-Ethoxyethoxy)propanoic acid | Endogenous | 1.245 min | 0.86% | 71991 | 2.22% |
| Adipoyl-CoA | Endogenous | 10.791 min | 0.52% | 37354 | 4.17% |
| Chondroitin sulfate | Endogenous | 9.102 min | 0.20% | 14847 | 4.58% |
| Deoxyguanosine | Endogenous | 4.009 min | 0.69% | 203394 | 4.08% |
| Hexanoyl-CoA | Endogenous | 10.887 min | 0.45% | 33408 | 2.52% |
| Homocysteic acid | Endogenous | 1.823 min | 1.26% | 24017 | 3.73% |
| Isoleucine | Endogenous | 5.917 min | 0.59% | 341768 | 7.50% |
| Nonanoylglycine | Endogenous | 8.834 min | 0.10% | 14638 | 6.91% |
| Sorbitol | Endogenous | 1.028 min | 0.26% | 19069 | 5.86% |
| Uric acid | Endogenous | 2.281 min | 1.39% | 77220 | 7.77% |

**Table S1b: Instrument QC’s for the mouse and human BAL aqueous fraction.** A pooled QC sample was run after every 5 sample injections to monitor instrument conditions. (n= 5 QC injections)

| **Compound** | **Type** | **Retention Time & %CV** | | **Abundance & %CV** | |
| --- | --- | --- | --- | --- | --- |
| *cis*-10-Nonadecenoic acid | Internal Standard | 3.247 min | 0.09% | 527947 | 3.17% |
| Ceramide(d18:1/17:0) | Internal Standard | 5.944 min | 0.07% | 521097 | 4.73% |
| PC (15:0/15:0) | Internal Standard | 4.940 min | 0.05% | 2906901 | 3.29% |
| PC (17:0/17:0) | Internal Standard | 5.812 min | 0.04% | 875523 | 2.32% |
| Testosterone-d2 | Internal Standard | 1.028 min | 0.30% | 4355052 | 3.00% |
| Triglyceride-d5(14:0/16:1/14:0) | Internal Standard | 8.159 min | 0.04% | 39568 | 3.72% |
| Bilirubin | Endogenous | 2.137 min | 0.12% | 50367 | 3.67% |
| CE(18:3) | Endogenous | 8.734 min | 0.05% | 37808 | 4.17% |
| Ceramide(d18:0/18:1) | Endogenous | 6.215 min | 0.07% | 649252 | 3.29% |
| CL(64:0) | Endogenous | 3.990 min | 0.06% | 26423 | 3.47% |
| Coenzyme Q10 | Endogenous | 8.149 min | 0.05% | 10132 | 3.43% |
| DG(32:1) | Endogenous | 6.202 min | 0.05% | 38720 | 3.03% |
| LysoPC(20:4) | Endogenous | 1.396 min | 0.18% | 2154332 | 2.74% |
| LysoPE(18:0) | Endogenous | 2.041 min | 0.11% | 256457 | 3.13% |
| Mesobilirubinogen | Endogenous | 1.564 min | 0.16% | 59413 | 3.93% |
| PC (36:3) | Endogenous | 5.375 min | 0.06% | 39507702 | 2.36% |
| SM(d18:0/24:1) | Endogenous | 7.288 min | 0.09% | 2690834 | 4.30% |
| TG(52:3) | Endogenous | 8.886 min | 0.04% | 520884 | 1.86% |
| TG(52:5) | Endogenous | 8.293 min | 0.03% | 145154 | 2.78% |

**Table S1c: Instrument QC’s for the mouse and human BAL and plasma lipid fraction.** A pooled QC sample was run after every 5 sample injections to monitor instrument conditions. Both lower abundant as well as higher abundant endogenous metabolites have %CV’s lower than 5%. (n = 9 QC injections)

# Supplemental Table S2: Fragmentation patterns of selected detected metabolites in the mouse and human samples

Tandem MS metabolite fragments were confirmed using NIST14 MSMS mass spectral library. NIST scores range from 0-999 where 999 is the best score. Fragmentation patterns are shown below the summary table. For each compound, the fragmentation spectra in red (top) represent the experimental spectra. This is denoted by a green question mark to indicate that it is unknown/experimental. The spectra in blue (bottom) shows the fragmentation pattern of the compound with the closest match to the NIST14 MSMS spectral database, and is based on a reference standard. The Match Factor (MF) is the normalized dot product with square-root scaling of the experimental mass spectrum and a library mass spectrum, using all the elements in the experimental mass spectrum. The Reverse Match Factor (RMF) is the normalized dot product with square-root scaling of the experimental mass spectrum and the library mass spectrum, but the elements that are not present in the library mass spectrum are not included.. The compound name is indicated in the bottom right in blue font of each box.

| **Metabolite** | **Formula** | **Precursor m/z** | **Precursor Adduct** | **Collision Energy** | **Frag 1** | **Frag 2** | **Frag 3** | **Frag 4** | **Frag 5** | **Frag 6** | **Frag 7** | **NIST Score** | **NIST Dot Score** | **NIST Rev-Dot Score** | **RT (min)** |
| --- | --- | --- | --- | --- | --- | --- | --- | --- | --- | --- | --- | --- | --- | --- | --- |
| **HILIC** |  |  |  |  |  |  |  |  |  |  |  |  |  |  |  |
| γ-Butyrobetaine, methyl ester | C8H18NO2 | 160.13 | [Cation]+ | 20 eV | 101.06 |  |  |  |  |  |  | 559 | 999 | 999 | 1.583 |
| 1,6-Anhydro-β-D-glucopyranose | C6H10O5 | 145.05 | [M+H-H2O]+ | 20 eV | 97.02 | 85.03 | 81.03 | 69.03 | 61.03 | 55.03 | 41.04 | 597 | 829 | 874 | 0.913 |
| 1-Methylnicotinamide | C7H9N2O | 137.07 | [Cation]+ | 40 eV | 93.05 | 79.04 | 66.04 | 65.04 | 52.03 | 51.02 | 39.02 | 517 | 779 | 796 | 6.455 |
| 3-Acetoxypyridine | C7H7NO2 | 138.05 | [M+H]+ | 20 eV | 96.07 |  |  |  |  |  |  | 405 | 811 | 999 | 4.265 |
| Acetylcarnitine | C9H17NO4 | 204.12 | [M+H]+ | 10 eV | 145.05 | 144.10 | 85.03 | 60.08 |  |  |  | 998 | 999 | 999 | 5.883 |
| Acetylcholine | C7H16NO2 | 146.12 | [Cation]+ | 10 eV | 87.05 | 60.08 | 43.02 |  |  |  |  | 766 | 948 | 973 | 8.300 |
| Adenosine | C10H13N5O4 | 268.10 | [M+H]+ | 40 eV | 136.06 | 119.03 |  |  |  |  |  | 654 | 993 | 994 | 4.017 |
| Arginine | C6H14N4O2 | 175.12 | [M+H]+ | 10 eV | 158.09 | 130.09 | 116.07 | 112.08 | 70.06 | 60.05 |  | 922 | 963 | 977 | 10.706 |
| Bestatin | C16H24N2O4 | 309.18 | [M+H]+ | 40 eV | 133.06 | 120.08 | 115.05 | 103.05 | 91.05 | 86.10 | 77.04 | 865 | 961 | 978 | 0.980 |
| Betaine | C5H11NO2 | 118.08 | [M+H]+ | 20 eV | 59.07 | 58.07 |  |  |  |  |  | 537 | 726 | 993 | 3.662 |
| Carnitine | C7H15NO3 | 162.11 | [M+H]+ | 20 eV | 103.04 | 102.09 | 85.03 | 60.08 | 59.07 | 57.03 | 43.02 | 944 | 981 | 982 | 6.584 |
| Choline | C5H14NO | 104.11 | [Cation]+ | 20 eV | 60.08 | 59.06 | 58.07 | 45.03 | 44.05 |  |  | 915 | 962 | 969 | 5.051 |
| Creatine | C4H9N3O2 | 132.07 | [M+H]+ | 10 eV | 90.05 | 44.05 |  |  |  |  |  | 801 | 978 | 978 | 4.175 |
| Creatinine | C4H7N3O | 114.07 | [M+H]+ | 20 eV | 114.07 | 86.07 | 72.04 | 44.05 | 43.03 |  |  | 502 | 886 | 997 | 0.765 |
| DL-2-Aminocaprylic acid | C8H17NO2 | 160.13 | [M+H}+ | 40 eV | 55.05 | 43.02 |  |  |  |  |  | 369 | 889 | 889 | 8.959 |
| Glucose | C6H12O6 | 198.09 | [M+NH4]+ | 10 eV | 180.07 | 163.06 | 145.05 | 127.04 | 91.00 | 85.03 | 61.00 | 826 | 921 | 921 | 0.971 |
| Glutamine | C5H10N2O3 | 147.07 | [M+H]+ | 20 eV | 130.05 | 84.04 | 56.05 | 41.04 |  |  |  | 701 | 969 | 981 | 4.518 |
| Glutathione, oxidized | C20H32N6O12S2 | 307.08 | [M+2H]2+ | 10 eV | 484.11 | 409.08 | 355.07 | 307.08 | 231.04 | 177.03 | 130.04 | 694 | 789 | 902 | 1.743 |
| Hypoxanthine | C5H4N4O | 137.05 | [M+H]+ | 20 eV | 119.03 | 110.03 | 94.04 | 82.04 | 67.03 | 65.01 | 55.03 | 724 | 835 | 955 | 0.583 |
| Leucine | C6H13NO2 | 132.10 | [M+H]+ | 20 eV | 86.09 | 69.07 | 44.05 | 43.05 | 41.04 | 30.03 |  | 859 | 926 | 955 | 1.921 |
| Leupeptin | C20H38N6O4 | 427.30 | [M+H]+ | 20 eV | 409.29 | 367.27 | 212.17 | 156.1 | 141.10 | 99.09 | 86.09 | 973 | 978 | 991 | 1.516 |
| Lysine | C6H14N2O2 | 147.11 | [M+H]+ | 10 eV | 130.08 | 84.08 | 56.05 |  |  |  |  | 574 | 949 | 979 | 11.081 |
| LysoPC(16:0) | C24H50NO7P | 496.34 | [M+H]+ | 40 eV | 184.07 | 166.06 | 124.99 | 104.11 | 86.09 | 71.07 | 60.08 | 944 | 963 | 977 | 3.437 |
| LysoPC(18:0) | C26H54NO7P | 546.35 | [M+Na]+ | 20 eV | 487.28 | 341.29 | 146.98 | 104.11 | 86.10 |  |  | 762 | 887 | 956 | 3.814 |
| Mannitol | C6H14O6 | 183.08 | [M+H]+ | 10 eV | 147.06 | 129.05 | 111.04 | 99.05 | 85.03 | 69.03 | 57.03 | 872 | 917 | 918 | 0.983 |
| N-Acetylalanine | C5H9NO3 | 132.06 | [M+H]+ | 20 eV | 90.05 | 44.05 |  |  |  |  |  | 537 | 973 | 994 | 4.617 |
| PC(16:0/16:0) | C40H80NO8P | 734.56 | [M+H]+ | 20 eV | 184.07 | 86.1 |  |  |  |  |  | 648 | 830 | 843 | 2.303 |
| PC(16:0/18:2) | C42H80NO8P | 758.57 | [M+H]+ | 40 eV | 184.07 | 124.99 | 98.98 | 86.10 | 60.08 |  |  | 622 | 948 | 968 | 2.002 |
| PC(18:1/18:1) | C44H84NO8P | 808.58 | [M+Na]+ | 40 eV | 749.51 | 625.52 | 603.53 | 184.07 | 416.98 | 86.10 |  | 613 | 826 | 855 | 2.399 |
| Phenylalanine | C9H11NO2 | 166.08 | [M+H]+ | 20 eV | 120.08 | 107.05 | 103.05 | 93.07 | 79.05 | 77.04 |  | 910 | 989 | 989 | 1.690 |
| Phenyllactic acid | C9H10O3 | 166.08 | [M+H]+ | 40 eV | 103.05 | 91.05 | 79.05 | 77.04 | 51.02 |  |  | 715 | 928 | 967 | 1.718 |
| Pyroglutamic acid | C5H7NO3 | 130.04 | [M+H]+ | 10 eV | 84.04 | 56.05 |  |  |  |  |  | 618 | 943 | 994 | 4.522 |
| SM(d18:1/24:1) | C47H93N2O6P | 813.61 | [M+H]+ | 40 eV | 184.07 | 125 | 104.11 | 86.10 | 60.10 |  |  | 407 | 936 | 985 | 3.025 |
| Tagatose | C6H12O6 | 198.09 | [M+NH4]+ | 10 eV | 180.20 | 163.06 | 145.05 | 127.04 | 85.03 | 61.03 |  | 593 | 874 | 874 | 0.966 |
| Taurine | C2H7NO3S | 126.02 | [M+H]+ | 10 eV | 108.00 | 44.05 |  |  |  |  |  | 817 | 987 | 988 | 1.393 |
| Tetraethylene glycol | C8H18O5 | 195.12 | [M+H]+ | 10 eV | 133.08 | 89.05 | 45.03 |  |  |  |  | 649 | 937 | 964 | 0.473 |
| Tropic acid | C9H10O3 | 166.08 | [M+H]+ | 40 eV | 103.05 | 91.05 | 79.05 | 77.04 |  |  |  | 745 | 886 | 937 | 1.692 |
| Tryptophan | C11H12N2O2 | 205.09 | [M+H]+ | 10 eV | 143.07 | 142.06 | 118.06 | 117.05 | 116.05 | 115.05 | 91.05 | 756 | 903 | 904 | 1.412 |
| Tyrosine | C9H11NO3 | 182.08 | [M+H]+ | 10 eV | 165.05 | 147.04 | 136.07 | 123.04 | 119.04 |  |  | 986 | 992 | 993 | 1.904 |
| Xanthine | C5H4N4O2 | 153.00 | [M+H]+ | 20 eV | 136.03 | 110.04 |  |  |  |  |  | 414 | 933 | 934 | 0.467 |
| **C18** |  |  |  |  |  |  |  |  |  |  |  |  |  |  |  |
| (Z)-13-Docosenamide | C22H43NO | 338.34 | [M+H]+ | 40 eV | 121.10 | 95.08 | 93.06 | 83.08 | 69.07 | 57.07 | 55.05 | 688 | 860 | 861 | 3.621 |
| 4-(2-Aminoethyl)benzenesulfonyl fluoride | C8H10FNO2S | 204.05 | [M+H]+ | 40 eV | 104.06 | 103.05 | 78.05 | 77.04 |  |  |  | 930 | 961 | 981 | 0.644 |
| 5α-Cholestan-3-one | C27H46O | 369.35 | [M+H-H2O]+ | 40 eV | 159.12 | 147.12 | 135.12 | 119.08 | 81.07 | 67.05 | 55.05 | 508 | 794 | 804 | 4.866 |
| Arachidonoylthio-PC | C44H82NO6PS | 784.57 | [M+H]+ | 40 eV | 184.07 | 124.99 | 104.10 | 86.09 | 60.08 |  |  | 560 | 978 | 987 | 5.087 |
| Ceramide (d18:1/16:0) | C34H67NO3 | 538.52 | [M+H]+ | 20 eV | 282.27 | 264.27 | 252.27 |  |  |  |  | 541 | 844 | 934 | 5.617 |
| Ceramide (d18:1/18:0) | C36H71NO3 | 566.55 | [M+H]+ | 10 eV | 548.54 | 530.52 | 264.27 |  |  |  |  | 625 | 976 | 990 | 6.200 |
| Cholesterol | C27H46O | 369.35 | [M+H-H2O]+ | 40 eV | 175.15 | 161.13 | 147.12 | 133.00 | 119.08 | 105.07 | 95.08 | 865 | 922 | 922 | 4.912 |
| DG(16:0/18:1) | C37H70O5 | 595.50 | [M+H]+ | 40 eV | 149.13 | 123.11 | 107.08 | 95.08 | 81.06 | 69.06 | 57.07 | 305 | 581 | 685 | 3.101 |
| Diisooctyl phthalate | C24H38O4 | 413.26 | [M+Na]+ | 20 eV | 301.14 | 189.01 | 171.00 |  |  |  |  | 676 | 968 | 996 | 3.133 |
| Eicosapentaenoyl PAF C-16 | C44H80NO7P | 766.53 | [M+H]+ | 10 eV | 184.07 |  |  |  |  |  |  | 408 | 606 | 647 | 5.734 |
| Glycerol 1-stearate | C21H42O4 | 359.31 | [M+H]+ | 40 eV | 95.08 | 81.07 | 69.07 | 57.07 | 55.05 |  |  | 677 | 848 | 875 | 2.872 |
| Linoleyl alcohol | C18H34O | 267.27 | [M+H]+ | 20 eV | 97.10 | 95.08 | 85.06 | 81.08 | 71.08 | 69.07 | 67.05 | 328 | 697 | 769 | 2.884 |
| LysoPC(14:0) | C22H46NO7P | 468.31 | [M+H]+ | 40 eV | 184.07 | 166.06 | 124.99 | 104.10 | 86.09 | 71.08 | 57.07 | 743 | 851 | 910 | 1.408 |
| LysoPC(16:0) | C24H50NO7P | 496.33 | [M+H]+ | 20 eV | 478.33 | 258.11 | 184.07 | 124.99 | 104.11 | 86.09 |  | 801 | 935 | 952 | 1.679 |
| LysoPC(17:0) | C25H52NO7P | 510.35 | [M+H]+ | 20 eV | 492.34 | 258.11 | 184.07 | 104.10 | 86.09 |  |  | 912 | 949 | 992 | 1.849 |
| LysoPC(18:0) | C26H54NO7P | 546.35 | [M+Na]+ | 40 eV | 487.27 | 341.30 | 146.98 | 104.11 | 86.10 | 71.09 | 57.07 | 789 | 901 | 910 | 2.083 |
| LysoPC(18:1) | C26H52NO7P | 522.35 | [M+H]+ | 40 eV | 339.29 | 184.07 | 166.06 | 125.00 | 104.11 | 86.10 | 60.08 | 919 | 946 | 955 | 1.541 |
| LysoPE(18:0) | C23H48NO7P | 482.32 | [M+H]+ | 10 eV | 464.31 | 341.30 | 216.06 | 62.06 | 44.05 |  |  | 580 | 723 | 974 | 2.111 |
| Oleamide | C18H35NO | 282.27 | [M+H]+ | 20 eV | 247.24 | 149.13 | 135.11 | 109.10 | 97.10 | 83.08 | 69.07 | 834 | 895 | 905 | 1.937 |
| Oleoyl ethylamide | C20H39NO | 310.31 | [M+H]+ | 20 eV | 135.12 | 128.11 | 121.10 | 97.10 | 83.08 | 69.07 | 57.07 | 443 | 658 | 688 | 2.936 |
| Palmitamide | C16H33NO | 256.26 | [M+H]+ | 20 eV | 131.12 | 102.09 | 97.09 | 88.07 | 71.08 | 69.07 | 57.07 | 639 | 857 | 883 | 1.800 |
| PC(14:0/16:0) | C38H76NO8P | 706.54 | [M+H]+ | 40 eV | 184.07 | 166.06 | 124.99 | 104.10 | 98.98 | 86.09 | 60.08 | 539 | 955 | 961 | 5.002 |
| PC(16:0/16:0) | C40H80NO8P | 756.55 | [M+Na]+ | 40 eV | 697.47 | 537.48 | 551.50 | 441.24 | 146.98 | 95.08 | 86.09 | 535 | 720 | 770 | 5.830 |
| PC(16:0/18:1) | C42H82NO8P | 782.56 | [M+Na]+ | 20 eV | 723.49 | 184.07 | 146.98 |  |  |  |  | 445 | 902 | 998 | 5.964 |
| PC(18:1/18:1) | C44H84NO8P | 786.60 | [M+H]+ | 20 eV | 184.07 | 86.10 |  |  |  |  |  | 650 | 988 | 999 | 5.770 |
| Phthalic acid | C8H6O4 | 149.02 | [M+H-H2O]+ | 20 eV | 121.03 | 93.03 | 65.04 |  |  |  |  | 956 | 979 | 979 | 1.480 |
| SM(d18:1/18:0) | C41H83N2O6P | 694.51 | [M+Na]+ | 20 eV | 694.51 | 570.52 |  |  |  |  |  | 521 | 895 | 970 | 5.382 |
| SM(d18:1/18:1) | C41H81N2O6P | 751.57 | [M+Na]+ | 20 eV | 692.49 | 568.50 | 184.07 | 86.09 |  |  |  | 734 | 917 | 965 | 4.824 |
| SM(d18:1/24:1) | C47H93N2O6P | 835.66 | [M+Na]+ | 20 eV | 776.59 | 652.59 |  |  |  |  |  | 623 | 934 | 942 | 6.489 |
| trans-Epoxysuccinyl-L-leucylamido(4-guanidino)butane | C15H27N5O5 | 358.21 | [M+H]+ | 20 eV | 299.16 | 218.10 | 200.09 | 114.10 | 72.08 |  |  | 699 | 878 | 901 | 0.601 |
| Tributyl phosphate | C12H27O4P | 289.15 | [M+Na]+ | 20 eV | 233.09 | 177.02 |  |  |  |  |  | 348 | 982 | 999 | 1.462 |
| Tris(2-butoxyethyl) phosphate | C18H39O7P | 421.23 | [M+Na]+ | 20 eV | 321.14 | 203.04 | 146.98 |  |  |  |  | 871 | 981 | 982 | 1.271 |
| **SB-AQ** |  |  |  |  |  |  |  |  |  |  |  |  |  |  |  |
| L-Methionine | C5H11NO2S | 150.05 | [M+H]+ | 10 eV | 133.03 | 104.05 | 102.05 | 74.06 | 61.01 | 56.05 |  | 923 | 959 | 985 | 1.327 |
| D-Pipecolinic acid | C6H11NO2 | 130.08 | [M+H]+ | 40 eV | 84.08 | 82.06 | 69.05 | 67.05 | 56.05 | 55.05 |  | 650 | 806 | 874 | 1.309 |
| N-Methylnicotinic acid | C7H8NO2 | 138.05 | [Cation]+ | 40 eV | 93.05 | 92.05 | 79.04 | 78.03 | 65.03 | 53.04 | 52.03 | 850 | 930 | 931 | 1.494 |
| Citric acid | C6H8O7 | 193.03 | [M+H]+ | 20 eV | 139.00 | 111.00 | 87.00 | 68.99 |  |  |  | 555 | 757 | 924 | 1.582 |
| O-Acetyl-L-serine | C5H9NO4 | 130.05 | [M+H-H2O]+ | 20 eV | 84.04 | 60.04 | 56.05 |  |  |  |  | 699 | 935 | 945 | 1.942 |
| DL-Octopamine | C8H11NO2 | 136.07 | [M+H-H2O]+ | 40 eV | 107.05 | 91.05 | 77.04 | 65.04 | 63.02 | 55.02 | 51.02 | 809 | 924 | 937 | 2.043 |
| Uridine | C9H12N2O6 | 245.07 | [M+H]+ | 20 eV | 113.03 | 96.01 | 73.03 | 70.03 | 69.03 | 57.03 | 55.02 | 497 | 920 | 964 | 2.521 |
| L-Kynurenine | C10H12N2O3 | 209.09 | [M+H]+ | 10 eV | 192.06 | 174.05 | 163.08 | 146.06 | 136.07 | 120.04 | 94.06 | 830 | 869 | 968 | 2.951 |
| Hypoxanthine | C5H4N4O | 137.04 | [M+H]+ | 20eV | 137.04 | 119.03 | 110.03 | 94.04 | 82.04 | 67.03 | 55.03 | 928 | 965 | 987 | 3.769 |
| Xanthine | C5H4N4O2 | 153.04 | [M+H]+ | 20eV | 136.01 | 110.03 | 82.04 | 81.01 | 55.03 |  |  | 846 | 921 | 939 | 4.474 |
| L-Tryptophan | C11H12N2O2 | 205.09 | [M+H]+ | 20eV | 188.07 | 170.06 | 159.09 | 146.06 |  | 132.08 | 118.06 | 971 | 973 | 988 | 5.383 |

----------------------------------------------------------------------------------------------------------------------------------------------------------------------------------------------------------------------

------------------------------------------------------------------------------------------------------------------------------------------------------------------------------------------------------------------------------------------------

# Supplemental Table S3: Annotated and matching fragments

Selected Annotated metabolites showing the isotopic ratios and distribution from Full Scan MS data annotated in ID Brower (Agilent), followed by their Tandem MS fragmentation patterns matched to the NIST14MSMS library. The top box shows the full scan data; the peaks in black are the experimental peaks and the boxes in red represent their theoretical isotopic ratios and distribution. The bottom box shows the fragmentation patterns of that compound. NIST scores range from 0-999 where 999 is the best score. Fragmentation patterns are shown below the summary table. For each compound, the fragmentation spectra in red (top) represent the experimental spectra. This is denoted by a green question mark to indicate that it is unknown/experimental. The spectra in blue (bottom) shows the fragmentation pattern of the compound with the closest match to the NIST14 MSMS spectral database, and is based on a reference standard. The Match Factor (MF) is the normalized dot product with square-root scaling of the experimental mass spectrum and a library mass spectrum, using all the elements in the experimental mass spectrum. The Reverse Match Factor (RMF) is the normalized dot product with square-root scaling of the experimental mass spectrum and the library mass spectrum, but the elements that are not present in the library mass spectrum are not included.. The compound name is indicated in the bottom right in blue font of each box.

# Supplemental Table S4: List of selected overlapping annotated metabolites in human and mouse BALF

Metabolites were annotated using exact mass, isotope ratios, and isotopic distribution. The listed metabolites have an error < 10ppm with database scores > 70 out of a possible 100.

| **Compound** | **Fraction** | **Score** | **Formula** | **RT (min)** | **ID** |
| --- | --- | --- | --- | --- | --- |
| α-CEHC | Lipid Pos | 72.56 | C16 H22 O4 | 1.352 | HMDB01518 |
| α-Hydroxy myristic acid | Lipid Pos | 94.75 | C14 H28 O3 | 1.142 | KEGG: C13790 |
| α-Tocopherol succinate | Lipid Pos | 84.08 | C33 H54 O5 | 1.498 | HMDB33685 |
| α-Tocopheronic acid | Lipid Pos | 87.90 | C16 H24 O5 | 3.171 | LMPR02020062 |
| β-D-Galactose | Aqueous | 90.17 | C6 H12 O6 | 1.083 | KEGG: C00962 |
| (±)-2-(1-Methylpropyl)-4,6-dinitrophenol | Lipid Neg | 85.09 | C10 H12 N2 O5 | 1.595 | KEGG: C14302 |
| (±)-2,4,6-Triphenyl-1-hexene | Lipid Pos | 71.31 | C24 H24 | 2.519 | KEGG: C14561 |
| (17Z)-1α,25-dihydroxy-26,27-dimethyl-17,20,22,22,23,23-hexadehydrovitamin D3 | Lipid Neg | 70.13 | C29 H42 O3 | 2.886 | LMST03020391 |
| (20R)-1α,25-dihydroxy-20-phenyl-16,17-didehydro-21-norvitamin D3 | Lipid Pos | 76.80 | C32 H44 O3 | 1.002 | LMST03020585 |
| (20S)-17,20-dihydroxypregn-4-en-3-one | Lipid Pos | 75.90 | C21 H32 O3 | 3.194 | KEGG: C04518 |
| (24R)-24-fluoro-1α,25-dihydroxyvitamin D2 | Lipid Pos | 84.64 | C28 H43 F O3 | 1.709 | LMST03010011 |
| (25S)-3α,7α,12α,24R-tetrahydroxy-5β-cholestan-26-oic acid | Lipid Pos | 83.76 | C27 H46 O6 | 3.177 | LMST04030194 |
| (25S)-3β-(2-O-methyl-β-D-xylopyranosyloxy)-cholestan-4β,5α,,8,15α,16β,26-hexol | Lipid Neg | 83.36 | C33 H58 O11 | 5.492 | LMST05050015 |
| (2S)-2-hydroxyphytanic acid | Lipid Pos | 83.50 | C20 H40 O3 | 3.175 | KEGG: C02982 |
| (3R)-oct-1-en-3-ol | Lipid Neg | 99.94 | C8 H16 O | 1.216 | LMFA05000093 |
| (3R,7R)-1,3,7-Octanetriol | Lipid Pos | 81.68 | C8 H18 O3 | 0.727 | HMDB33625 |
| (3R,7R)-1,3,7-Octanetriol | Lipid Pos | 72.59 | C8 H18 O3 | 1.082 | HMDB33625 |
| (3R,7R)-1,3,7-Octanetriol | Lipid Pos | 78.12 | C8 H18 O3 | 2.043 | HMDB33625 |
| (3β,17α,23S)-17,23-Epoxy-3,29-dihydroxy-27-norlanosta-7,9(11)-diene-15,24-dione | Lipid Pos | 83.21 | C29 H42 O5 | 3.172 | HMDB35970 |
| (E)-7-Pentadecene | Lipid Neg | 97.92 | C15 H30 | 3.225 | HMDB31083 |
| (R)-10-hydroxystearic acid | Lipid Pos | 72.25 | C18 H36 O3 | 2.927 | KEGG: C03195 |
| (R*,S*)-4-[1-Ethyl-2-(4-fluorophenyl)butyl]phenol | Lipid Pos | 76.16 | C18 H21 F O | 0.611 | KEGG: C15463 |
| (S)-10,16-Dihydroxyhexadecanoic acid | Lipid Pos | 92.68 | C16 H32 O4 | 1.155 | KEGG: C08285 |
| (S)-Neolyratyl acetate | Lipid Neg | 86.55 | C12 H18 O2 | 1.323 | HMDB41497 |
| (S,E)-Zearalenone | Lipid Pos | 75.27 | C18 H22 O5 | 2.470 | KEGG: C09981 |
| (Z)-3-Hexenyl hexadecanoate | Lipid Pos | 89.22 | C22 H42 O2 | 3.724 | LMFA07010591 |
| 1,2,3-trihexadecanoyl-sn-glycerol | Lipid Pos | 78.34 | C51 H98 O6 | 9.591 | HMDB05356 |
| 1,20-Eicosanediol | Lipid Pos | 81.49 | C20 H42 O2 | 5.497 | HMDB40982 |
| 1,26-Dicaffeoylhexacosanediol | Lipid Pos | 83.70 | C44 H66 O8 | 1.408 | HMDB30750 |
| 1,26-Hexacosanediol | Lipid Pos | 92.10 | C26 H54 O2 | 7.500 | HMDB36581 |
| 1,2-Cyclohexanediol | Lipid Pos | 73.86 | C6 H12 O2 | 10.453 | KEGG: C12313 |
| 1,2-tetracosanediol | Lipid Pos | 94.41 | C24 H50 O2 | 6.963 | LMFA05000083 |
| 1,3-Propanediol | Lipid Pos | 81.19 | C3 H8 O2 | 10.459 | KEGG: C02457 |
| 1-[1,4-Dihydro-4-nonyl-5-(1-oxodecyl)-3-pyridinyl]-1-dodecanone | Lipid Neg | 83.72 | C36 H65 N O2 | 6.800 | HMDB35518 |
| 10,12-Tetradecadienal | Lipid Pos | 91.81 | C14 H24 O | 1.143 | LMFA06000187 |
| 11-methyl-hexadecanoic acid | Lipid Pos | 93.60 | C17 H34 O2 | 3.178 | LMFA01020194 |
| 11R-HETE | Lipid Pos | 83.32 | C20 H32 O3 | 2.447 | KEGG: C14780 |
| 12'-Apo-b-carotene-3,12'-diol | Lipid Neg | 83.51 | C25 H36 O2 | 2.945 | HMDB36054 |
| 12-hydroxy-8E,10E-heptadecadienoic acid | Lipid Pos | 70.07 | C17 H30 O3 | 1.258 | LMFA01050194 |
| 12-Methyl myristic acid | Lipid Pos | 86.13 | C15 H30 O2 | 2.462 | KEGG: C16665 |
| 13,14-dihydroxy-docosanoic acid | Lipid Pos | 82.42 | C22 H44 O4 | 3.286 | LMFA01050211 |
| 13-Docosenamide* | Lipid Pos | 99.18 | C22 H43 N O | 3.708 | CAS: 112-84-5 |
| 13Z,16Z-docosadienoic acid | Lipid Pos | 93.85 | C22 H40 O2 | 3.150 | KEGG: C16533 |
| 14-methyl-1-hexadecanol | Lipid Pos | 78.53 | C17 H36 O | 3.292 | LMFA05000009 |
| 15-methylheneicosane-1,2-diol | Lipid Pos | 87.09 | C22 H46 O2 | 6.341 | LMFA05000080 |
| 15-methyl-hexadecasphingosine | Lipid Pos | 77.16 | C17 H35 N O2 | 3.716 | LMSP01080005 |
| 15-oxo-octadecanoic acid | Lipid Pos | 95.38 | C18 H34 O3 | 2.443 | LMFA02000259 |
| 16:0 Campesteryl ester | Lipid Neg | 90.33 | C44 H78 O2 | 7.553 | LMST01020043 |
| 16-methyl-9Z,12Z-heptadecadienoic acid | Lipid Neg | 99.08 | C18 H32 O2 | 2.864 | LMFA01020208 |
| 17-Hydroxymethylethisterone | Lipid Pos | 76.01 | C22 H30 O3 | 1.312 | HMDB60710 |
| 17-Methylandrosta-2,4-dieno[2,3-d]isoxazol-17β-ol | Lipid Pos | 80.41 | C21 H29 N O2 | 1.314 | KEGG: C15177 |
| 17-phenyl trinor PGF2α isopropyl ester | Lipid Pos | 93.62 | C26 H38 O5 | 1.990 | CAS: 130209-76-6 |
| 17β-Nitro-5α-androstane | Lipid Pos | 74.71 | C19 H31 N O2 | 2.465 | KEGG: C15281 |
| 18-Nor-4(19),8,11,13-abietatetraene | Lipid Neg | 85.32 | C19 H26 | 2.236 | HMDB41371 |
| 18-Oxocortisol | Lipid Pos | 92.13 | C21 H28 O6 | 0.759 | LMST02030194 |
| 19-(3-methyl-butanoyloxy)-villanovane-13α,17-diol | Lipid Pos | 90.93 | C25 H42 O5 | 3.177 | LMPR0104150003 |
| 19-hydroxy-nonadecanoic acid | Lipid Pos | 99.44 | C19 H38 O3 | 3.283 | LMFA01050072 |
| 19-hydroxy-nonadecanoic acid | Lipid Pos | 99.40 | C19 H38 O3 | 3.283 | LMFA01050072 |
| 1-Hexadecyl-2-O-methyl-glycerol | Lipid Pos | 87.80 | C20 H42 O3 | 3.180 | CAS: 111188-59-1 |
| 1-Methoxy-1-pentyloxyethane | Lipid Pos | 87.20 | C8 H18 O2 | 0.791 | HMDB38679 |
| 1-Methyluric acid | Lipid Pos | 83.22 | C6 H6 N4 O3 | 0.451 | KEGG: C16359 |
| 1-Nonadecene | Lipid Pos | 71.92 | C19 H38 | 7.012 | LMFA11000322 |
| 1-Octacosene | Lipid Neg | 99.03 | C28 H56 | 2.874 | LMFA11000324 |
| 1-O-β-D-Glucopyranosyl-2,3-di-O-(8-hexadecenoyl)glycerol | Lipid Neg | 89.00 | C41 H74 O10 | 6.365 | HMDB31133 |
| 1α,25-dihydroxy-24a,24b-didihomo-22-thia-20-epivitamin D3 | Lipid Pos | 78.97 | C28 H46 O3 S | 1.003 | LMST03020369 |
| 1α,25-dihydroxy-3-deoxy-3-thiavitamin D3 | Lipid Pos | 79.95 | C26 H42 O2 S | 1.032 | LMST03020041 |
| 2-(4-Allyl-2-methoxyphenoxy)-1-(4-hydroxy-3-methoxyphenyl)-1-propanol | Lipid Pos | 79.06 | C20 H24 O5 | 1.354 | HMDB31753 |
| 2-(8-[3]-ladderane-octanyl)-sn-glycero-3-phosphocholine | Lipid Pos | 83.78 | C28 H52 N O6 P | 1.101 | LMGP01060026 |
| 2-(9R-(tricosanoyloxy)-3-methyl-2Z-decenoyloxy)-ethanesulfonic acid | Lipid Pos | 83.53 | C36 H68 O7 S | 1.563 | LMFA07020004 |
| 2(R)-HPOT | Lipid Pos | 77.00 | C18 H30 O4 | 1.154 | KEGG: C16341 |
| 2,2,9,9-tetramethyl-undecan-1,10-diol | Lipid Pos | 77.70 | C14 H30 O2 | 1.157 | LMFA05000017 |
| 2,4-dimethyl-dodecanoic acid | Lipid Neg | 99.57 | C14 H28 O2 | 2.545 | LMFA01020161 |
| 2,6-dimethyl-pentadecanoic acid | Lipid Pos | 96.44 | C17 H34 O2 | 3.176 | LMFA01020040 |
| 2,8-dimethyl-tetradecanoic acid | Lipid Neg | 98.65 | C16 H32 O2 | 3.229 | LMFA01020039 |
| 2,8-dimethyl-tetradecanoic acid | Lipid Pos | 74.52 | C16 H32 O2 | 2.271 | LMFA01020039 |
| 20, 22-Dihydrodigoxigenin | Lipid Neg | 79.13 | C23 H36 O5 | 3.727 | HMDB60730 |
| 21-Deoxycortisol | Lipid Pos | 98.43 | C21 H30 O4 | 1.525 | KEGG: C05497 |
| 22,23-Methylene-24-methyl-cholest-5-en-3β-ol | Lipid Neg | 76.86 | C29 H48 O | 5.023 | LMST01030138 |
| 22-Acetylpriverogenin B | Lipid Pos | 70.58 | C32 H52 O5 | 1.318 | HMDB34645 |
| 24,24-Difluoro-25-hydroxy-26,27-dimethylvitamin D3 | Lipid Pos | 87.50 | C29 H46 F2 O2 | 1.132 | LMST03020676 |
| 24,24-Difluoro-25-hydroxy-26,27-dimethylvitamin D3 | Lipid Pos | 73.92 | C29 H46 F2 O2 | 3.737 | LMST03020676 |
| 24-ethyl-5α-cholest-25-en-3α,12α,16α-triol | Lipid Pos | 95.95 | C29 H50 O3 | 4.369 | LMST01040188 |
| 25-acetoxy-ergosta-3β,5α,6β-triol | Lipid Neg | 70.85 | C30 H52 O5 | 5.488 | LMST01031059 |
| 25-Azacholesterol | Lipid Pos | 76.67 | C26 H45 N O | 7.941 | HMDB01028 |
| 2-Aminobenzimidazole | Lipid Pos | 75.36 | C7 H7 N3 | 1.792 | KEGG: C10901 |
| 2-Butoxyethanol | Lipid Pos | 86.54 | C6 H14 O2 | 10.537 | KEGG: C19355 |
| 2-Decaprenyl-3-methyl-6-methoxy-1,4-benzoquinone | Lipid Pos | 88.72 | C58 H88 O4 | 8.797 | HMDB06484 |
| 2'-Deoxyguanosine 5'-monophosphate | Aqueous | 92.11 | C10 H14 N5 O7 P | 1.656 | KEGG: C00362 |
| 2-Deoxystreptidine | Lipid Pos | 73.18 | C8 H18 N6 O3 | 0.660 | KEGG: C02628 |
| 2E-Octen-1-ol | Lipid Pos | 78.58 | C8 H16 O | 1.128 | LMFA05000124 |
| 2-ethyl-1,5-dimethyl-3,3-diphenylpyrrolinium (EDDP) | Lipid Neg | 84.09 | C20 H24 N | 2.870 | HMDB60931 |
| 2-Heptenal | Lipid Pos | 71.44 | C7 H12 O | 1.145 | LMFA06000019 |
| 2-Heptyl butyrate | Lipid Pos | 72.76 | C12 H24 O2 | 1.191 | HMDB32310 |
| 2-Hydroxy-3-methylhexadecanoic acid | Lipid Neg | 73.74 | C17 H34 O3 | 6.523 | LMFA01050368 |
| 2-Hydroxy-docosanoic acid | Lipid Pos | 70.07 | C22 H44 O3 | 3.290 | LMFA01050077 |
| 2-Hydroxyenterodiol | Lipid Pos | 83.51 | C18 H22 O5 | 1.352 | HMDB41649 |
| 2-Hydroxy-heneicosanoic acid | Lipid Pos | 74.60 | C21 H42 O3 | 2.880 | LMFA01130002 |
| 2-Mercapto-octadecanoic acid | Lipid Pos | 80.78 | C18 H36 O2 S | 1.351 | LMFA01130002 |
| 2-Methyl-5,8,11,14-all-cis-tricosatetraenoyl-2'-fluoroethylamine | Lipid Pos | 83.96 | C26 H44 F N O | 3.266 | LMFA08020071 |
| 2-Oxo-4-methylthio-butanoic acid | Lipid Pos | 92.89 | C5 H8 O3 S | 10.484 | KEGG: C01180 |
| 2-Oxo-8-methylthiooctanoic acid | Lipid Pos | 77.63 | C9 H16 O3 S | 10.485 | KEGG: C17224 |
| 2S,4S,6S,8R,10R,12R-Hexamethyl-13-hydroxy-triacontanoic acid | Lipid Pos | 94.81 | C36 H72 O3 | 3.290 | LMFA01020323 |
| 2S-Dimethylaminooctadecane-1,3R-diol | Lipid Pos | 70.60 | C20 H43 N O2 | 2.863 | LMSP01080056 |
| 2S-Hydroxylauric acid | Lipid Pos | 95.00 | C12 H24 O3 | 1.115 | LMFA01050362 |
| 2β,3α,12α-Trihydroxy-5β-cholan-24-oic Acid | Lipid Pos | 93.64 | C24 H40 O5 | 1.992 | LMST04010076 |
| 2β,3α,12α-Trihydroxy-5β-cholan-24-oic Acid | Lipid Pos | 95.02 | C24 H40 O5 | 1.992 | LMST04010076 |
| 3-(2,4-Cyclopentadien-1-ylidene)-5α-androstan-17β-ol | Lipid Pos | 77.87 | C24 H34 O | 3.568 | KEGG: C14915 |
| 3,5-Dimethylpentadecanoic acid | Lipid Neg | 99.68 | C17 H34 O2 | 3.614 | LMFA01020390 |
| 3,7,11,15-Tetramethyl-6,10,14-hexadecatrien-1-ol | Lipid Pos | 76.06 | C20 H36 O | 1.311 | LMFA05000210 |
| 31-Hydroxy-32,35-anhydrobacteriohopanetetrol | Lipid Neg | 96.20 | C35 H60 O4 | 2.867 | LMPR04000027 |
| 35-Aminobacteriohopane-30,31,32,33,34-pentol | Lipid Pos | 86.76 | C35 H63 N O5 | 1.584 | LMPR04000009 |
| 3b-Allotetrahydrocortisol | Lipid Neg | 78.55 | C21 H34 O5 | 3.613 | HMDB00314 |
| 3-Hexadecanoyloleanolic acid | Lipid Pos | 84.77 | C46 H78 O4 | 7.517 | HMDB36967 |
| 3-Hexanone | Lipid Neg | 99.38 | C6 H12 O | 0.855 | HMDB00753 |
| 3-Hydroxy-2-methyl-3-phytyl-2,3-dihydro-1,4-naphthoquinone | Lipid Pos | 81.16 | C31 H48 O3 | 1.676 | KEGG: C02785 |
| 3-Hydroxy-2-methylpyridine-4,5-dicarboxylate | Lipid Neg | 85.77 | C8 H7 N O5 | 0.633 | KEGG: C04604 |
| 3-Methyl-2Z-pentenoic acid | Lipid Pos | 76.86 | C6 H10 O2 | 1.145 | LMFA01020114 |
| 3-Methyldioxyindole | Lipid Pos | 72.23 | C9 H9 N O2 | 1.223 | KEGG: C05834 |
| 3-Methylhistamine | Lipid Neg | 95.35 | C6 H11 N3 | 2.878 | HMDB01861 |
| 3-Methyl-tetradecanedioic acid | Lipid Pos | 74.49 | C15 H28 O4 | 1.277 | LMFA01170019 |
| 3-Octaprenyl-4-hydroxybenzoate | Lipid Pos | 95.00 | C47 H70 O3 | 7.150 | KEGG: C05809 |
| 3-Oxocholic acid | Lipid Pos | 92.98 | C24 H38 O5 | 1.664 | HMDB00502 |
| 3-Oxododecanoic acid | Lipid Pos | 83.45 | C12 H22 O3 | 0.969 | KEGG: C02367 |
| 3-Oxosteroid | Lipid Pos | 83.51 | C19 H30 O | 1.992 | KEGG: C01876 |
| 3Z-Octadecenoic acid | Lipid Neg | 99.92 | C18 H34 O2 | 3.360 | LMFA01030294 |
| 3α,4β,12α-Trihydroxy-5β-cholan-24-oic Acid | Lipid Pos | 92.93 | C24 H40 O5 | 1.990 | LMST04010077 |
| 3α,7α,12α-Trihydroxy-27-carboxymethyl-5β-Cholestan-26-oic acid | Lipid Pos | 77.59 | C29 H48 O7 | 1.144 | LMST04080001 |
| 3β,6α-Diacetoxy-5α-pregn-20-ene | Lipid Pos | 89.75 | C26 H40 O3 | 2.265 | LMST02030215 |
| 4,4'-(Diphenylethenylidene)bis[N,N-dimethylbenzenamine] | Lipid Pos | 78.94 | C30 H30 N2 | 0.984 | KEGG: C15021 |
| 4,4-Difluoro-1α-hydroxyvitamin D3 | Lipid Pos | 87.31 | C27 H42 F2 O2 | 3.736 | LMST03020138 |
| 4,6-Nonadecanedione | Lipid Neg | 99.29 | C19 H36 O2 | 3.728 | HMDB35575 |
| 4-Amino-4-deoxy-α-L-arabinopyranosyl undecaprenyl phosphate | Lipid Pos | 90.42 | C60 H100 N O7 P | 3.284 | KEGG: C16157 |
| 4-Dodecylbenzenesulfonic Acid | Lipid Neg | 98.19 | C18 H30 O3 S | 2.337 | HMDB59915 |
| 4-Ethyl-5-pentyloxazole | Lipid Neg | 80.44 | C10 H17 N O | 3.355 | HMDB37864 |
| 4-Keto lauric acid | Lipid Pos | 90.49 | C12 H22 O3 | 0.970 | LMFA01060039 |
| 4-Tetradecanamidobenzylphosphonic acid | Lipid Pos | 74.74 | C21 H36 N O4 P | 1.012 | CAS: 1096770-84-1 |
| 5-(10,13-Nonadecadienyl)-1,3-benzenediol | Lipid Pos | 76.06 | C25 H40 O2 | 3.189 | HMDB39867 |
| 5,3'-Digeranyl-3,4,2',4'-tetrahydroxychalcone | Lipid Pos | 89.93 | C35 H44 O5 | 1.337 | LMPK12120107 |
| 5,7-Dimethoxy-6-C-methylflavone | Lipid Pos | 82.49 | C18 H16 O4 | 1.083 | LMPK12110173 |
| 5,7-Heptadecadiene | Lipid Neg | 99.39 | C17 H32 | 3.360 | LMFA11000443 |
| 5,8,11,14-Docosatetraynoic acid | Lipid Neg | 80.42 | C22 H28 O2 | 2.647 | LMFA01030681 |
| 5E,8Z,11Z,14Z,16Z-Eicosapentaenoic acid | Lipid Neg | 93.29 | C20 H30 O2 | 2.648 | LMFA01030397 |
| 5-Ethyl-3-methyl-2E,4E,6E-nonatriene | Lipid Pos | 80.44 | C12 H20 | 2.520 | LMFA11000051 |
| 5-Hydroperoxy-7-[3,5-epidioxy-2-(2-octenyl)-cyclopentyl]-6-heptenoic acid | Lipid Neg | 78.18 | C19 H30 O6 | 1.709 | LMFA01040028 |
| 5-Hydroxylysine | Lipid Pos | 70.12 | C6 H14 N2 O3 | 1.017 | KEGG: C16741 |
| 5-Methyl-2-pentylthiazole | Lipid Pos | 71.40 | C9 H15 N S | 0.847 | HMDB40099 |
| 5S,12R-Dihydroxy-6Z,8E,10E,14Z-eicosatetraenoic acid-d4 | Lipid Pos | 71.15 | C20 H28 D4 O4 | 2.466 | KEGG: C02165 |
| 5α-Cholesta-8,24-dien-3β-yl (9Z)-octadec-9-enoate | Lipid Pos | 86.34 | C45 H76 O2 | 7.416 | LMST01020034 |
| 6-Hydroxydelphinidin 3-glucoside | Lipid Pos | 80.89 | C21 H21 O13 | 1.353 | LMPK12010431 |
| 6-Hydroxyluteolin 6,3'-dimethyl ether 7,4'-disulfate | Lipid Neg | 75.63 | C17 H14 O13 S2 | 1.674 | LMPK12111259 |
| 6-Keto-decanoylcarnitine | Lipid Pos | 70.27 | C17 H31 N O5 | 3.561 | HMDB13202 |
| 6-Methyltetrahydropterin | Lipid Pos | 86.31 | C7 H11 N5 O | 0.695 | HMDB02249 |
| 6-O-[3-hydroxy-2-tetradecyloctadec-11E-enoyl]-α-D-glucopyranosyl α-D-glucopyranoside | Lipid Pos | 88.19 | C44 H82 O13 | 2.932 | KEGG: C04218 |
| 6α-Fluoropregn-4-ene-3,11,20-trione | Lipid Pos | 78.63 | C21 H27 F O3 | 1.031 | KEGG: C15327 |
| 6α-Hydroxycampestanol | Lipid Pos | 86.79 | C28 H50 O2 | 2.206 | KEGG: C15788 |
| 7',8'-Dihydro-8'-hydroxyreticulataxanthin | Lipid Pos | 74.92 | C33 H46 O3 | 1.674 | HMDB39090 |
| 7-Heptadecene | Lipid Neg | 99.36 | C17 H34 | 4.016 | LMFA11000505 |
| 7-Ketodeoxycholic acid | Lipid Pos | 89.25 | C24 H38 O5 | 1.663 | HMDB00391 |
| 7-O-Acetylaustroinulin | Lipid Neg | 80.71 | C22 H36 O4 | 3.854 | HMDB36804 |
| 7S,8S-DiHOME(9Z) | Lipid Pos | 99.04 | C18 H34 O4 | 2.041 | KEGG: C07355 |
| 8E-Dodecenyl acetate | Lipid Pos | 92.91 | C14 H26 O2 | 1.142 | LMFA05000275 |
| 8-Hydroxypurine | Lipid Pos | 72.89 | C5 H6 N4 O | 3.146 | HMDB12182 |
| 8-Methyl-3-hentriacontene | Lipid Neg | 98.83 | C32 H64 | 3.613 | HMDB35279 |
| 8Z,11Z,14Z-Heptadecatrienoic acid | Lipid Pos | 71.52 | C17 H28 O2 | 2.474 | KEGG: C16344 |
| 9,10,16-Trihydroxy palmitic acid | Lipid Pos | 79.74 | C16 H32 O5 | 1.579 | LMFA01050101 |
| 9,12,14-Octadecatrienoic acid | Lipid Neg | 81.75 | C18 H30 O2 | 3.228 | LMFA01030151 |
| 9,12-Hexadecadienylcarnitine | Lipid Pos | 72.74 | C25 H45 N O4 | 3.721 | LMFA07070009 |
| 9-cis-Retinoic acid | Lipid Neg | 94.12 | C20 H28 O2 | 2.237 | KEGG: C15493 |
| 9Z-octadecenyl 5Z,8Z,11Z,14Z-eicosatetraenoate | Lipid Neg | 91.31 | C38 H66 O2 | 6.215 | LMFA07010161 |
| Acetylcarnitine* | Aqueous | 99.42 | C9 H18 N O4 | 1.493 | KEGG: C02571 |
| Ala Ile Ile | Lipid Pos | 78.69 | C15 H29 N3 O4 | 1.311 |  |
| All-trans-8'-Apo-β-carotenal | Lipid Pos | 74.40 | C30 H40 O | 1.968 | KEGG: C19728 |
| All-trans-Carophyll yellow | Lipid Pos | 75.42 | C32 H44 O2 | 1.874 | HMDB32879 |
| Alosetron | Lipid Neg | 86.35 | C17 H18 N4 O | 1.479 | HMDB15104 |
| Altretamine | Lipid Pos | 84.04 | C9 H18 N6 | 0.945 | HMDB14631 |
| Anandamide (20:2, n-6) | Lipid Pos | 79.99 | C22 H41 N O2 | 2.716 | LMFA08040002 |
| Anandamide (20:l, n-9) | Lipid Pos | 99.09 | C22 H43 N O2 | 3.148 | LMFA08040010 |
| Arginyl-Proline | Lipid Pos | 72.44 | C11 H21 N5 O3 | 1.026 | HMDB28717 |
| Armillaric acid | Lipid Pos | 73.07 | C23 H28 O7 | 0.758 | HMDB37040 |
| Asp Asp Asp | Lipid Pos | 78.19 | C12 H17 N3 O10 | 1.354 |  |
| Aspidospermidine | Lipid Neg | 83.63 | C19 H26 N2 | 3.614 | HMDB30360 |
| Aspidospermine | Lipid Neg | 83.57 | C22 H30 N2 O2 | 3.726 | KEGG: C09042 |
| Avocadene 1-acetate | Lipid Neg | 96.47 | C19 H36 O4 | 5.774 | HMDB31043 |
| Azaspiracid | Lipid Neg | 91.57 | C47 H71 N O12 | 5.617 | HMDB33805 |
| Bacteriohopane-31,32,33,34-tetrol-35-cyclitol | Lipid Neg | 95.88 | C41 H73 N O9 | 5.781 | LMPR04000008 |
| Behenoyl-EA | Lipid Pos | 84.30 | C24 H49 N O2 | 3.716 | LMFA08040052 |
| Behenoyl-EA | Lipid Pos | 91.86 | C24 H49 N O2 | 4.499 | LMFA08040052 |
| Behenoyl-ethanolamine | Lipid Pos | 86.81 | C24 H49 N O2 | 3.722 | LMFA08040052 |
| Betavulgaroside IX | Lipid Pos | 83.37 | C52 H80 O24 | 6.043 | HMDB34869 |
| Bisphenol A | Lipid Pos | 80.93 | C15 H16 O2 | 1.312 | KEGG: C13624 |
| C16 Sphinganine | Lipid Pos | 81.44 | C16 H35 N O2 | 1.310 | KEGG: C00836 |
| C19 Sphingosine-1-phosphate | Lipid Pos | 94.97 | C19 H40 N O5 P | 0.999 | KEGG: C06124 |
| Calicoferol D | Lipid Neg | 81.31 | C28 H42 O2 | 4.725 | LMST03020313 |
| Carisoprodol | Lipid Pos | 70.14 | C12 H24 N2 O4 | 1.303 | KEGG: C07927 |
| Carnitine* | Aqueous | 99.11 | C7 H16 N O3 | 1.140 | KEGG: C00487 |
| Carnocin U I49 | Lipid Pos | 89.11 | C22 H26 N2 O | 1.274 | HMDB38223 |
| CE(12:0) | Lipid Neg | 95.50 | C39 H68 O2 | 3.728 | HMDB02262 |
| CE(12:0) | Lipid Neg | 89.77 | C39 H68 O2 | 6.511 | HMDB02262 |
| CE(15:0) | Lipid Neg | 90.43 | C42 H74 O2 | 7.080 | HMDB60057 |
| CE(20:4(8Z,11Z,14Z,17Z)) | Lipid Neg | 87.44 | C47 H76 O2 | 2.874 | HMDB10371 |
| CE(22:0) | Lipid Pos | 86.54 | C49 H88 O2 | 3.283 | KEGG: C02530 |
| CE(22:4(7Z,10Z,13Z,16Z) | Lipid Neg | 84.62 | C49 H80 O2 | 3.615 | KEGG: C02530 |
| CE(22:4(7Z,10Z,13Z,16Z) | Lipid Neg | 85.46 | C49 H80 O2 | 3.229 | KEGG: C02530 |
| CE(22:5(4Z,7Z,10Z,13Z,16Z)) | Lipid Pos | 73.40 | C49 H78 O2 | 7.174 | HMDB10374 |
| Ceramide (d14:1(4E)/22:0(2OH)) | Lipid Neg | 97.36 | C36 H71 N O4 | 6.515 | LMSP02010070 |
| Ceramide (d14:1(4E)/22:0(2OH)) | Lipid Neg | 96.39 | C36 H71 N O4 | 6.225 | LMSP02010070 |
| Ceramide (d16:1(4E)/22:0(2OH)) | Lipid Neg | 96.85 | C38 H75 N O4 | 6.794 | LMSP02010085 |
| Ceramide (d16:1/17:0) | Lipid Pos | 88.45 | C33 H65 N O3 | 5.445 | KEGG: C00195 |
| Ceramide (d18:0/16:0) | Lipid Neg | 96.89 | C34 H69 N O3 | 6.449 | HMDB11760 |
| Ceramide (d18:0/18:0(2OH)) | Lipid Pos | 92.36 | C36 H73 N O4 | 5.758 | LMSP02020030 |
| Ceramide (d18:0/18:1(11Z)) | Lipid Neg | 99.73 | C36 H71 N O3 | 6.801 | HMDB11762 |
| Ceramide (d18:0/22:0) | Lipid Pos | 84.80 | C40 H81 N O3 | 6.607 | HMDB11765 |
| Ceramide (d18:1/16:0)* | Lipid Pos | 98.39 | C34 H67 N O3 | 5.732 | KEGG: C00195 |
| Ceramide (d18:1/18:0)* | Lipid Pos | 96.67 | C36 H71 N O3 | 6.296 | KEGG: C00195 |
| Ceramide (d18:1/24:0) | Lipid Pos | 90.86 | C42 H83 N O3 | 7.607 | KEGG: C00195 |
| Ceramide (d18:1/24:1(15Z)) | Lipid Pos | 88.21 | C42 H81 N O3 | 7.228 | KEGG: C00195 |
| Ceramide (d20:0/20:0(2OH)) | Lipid Pos | 91.94 | C40 H81 N O4 | 8.120 | LMSP02020037 |
| CerP(d18:1/16:0) | Lipid Neg | 99.17 | C34 H68 N O6 P | 5.519 | HMDB10700 |
| Cetyl alcohol | Lipid Pos | 96.45 | C16 H34 O | 1.597 | KEGG: C00823 |
| Cetyl alcohol | Lipid Pos | 72.03 | C16 H34 O | 3.085 | KEGG: C00823 |
| Chenodeoxyglycocholate | Lipid Pos | 90.89 | C26 H43 N O5 | 3.172 | KEGG: C05466 |
| Cholest-5-en-3β-yl (5Z,8Z,11Z,14Z,17Z-eicosapentaenoate) | Lipid Pos | 82.20 | C47 H74 O2 | 6.769 | HMDB06731 |
| Cholest-5-en-3β-yl dodecanoate | Lipid Neg | 89.99 | C39 H68 O2 | 6.796 | HMDB02262 |
| Cholest-5-en-3β-yl hydrogen sulfate | Lipid Neg | 98.54 | C27 H46 O4 S | 4.385 | LMST05020016 |
| Cholesterol* | Lipid Pos | 98.38 | C27 H46 O | 5.007 | KEGG: C00187 |
| Cis-Jasmone | Lipid Pos | 89.40 | C11 H16 O | 10.484 | KEGG: C08490 |
| CL(16:0/18:0/16:0/18:0) | Lipid Pos | 86.84 | C77 H150 O17 P2 | 3.404 | KEGG: C05980 |
| CL(16:0/18:2/18:0/18:0) | Lipid Pos | 74.73 | C80 H152 O17 P2 | 5.414 | KEGG: C05980 |
| CL(18:0/16:0/18:0/18:0) | Lipid Neg | 95.00 | C79 H154 O17 P2 | 5.671 | HMDB56923 |
| CL(18:0/18:0/22:5/22:5) | Lipid Neg | 70.60 | C89 H154 O17 P2 | 5.580 | HMDB57026 |
| Clemastine | Lipid Pos | 80.44 | C21 H26 Cl N O | 0.922 | KEGG: C06913 |
| Coniferyl alcohol | Lipid Pos | 91.41 | C10 H12 O3 | 1.216 | KEGG: C00590 |
| Creatine | Aqueous | 87.43 | C4 H9 N3 O2 | 1.151 | KEGG: C00300 |
| Cucurbitaxanthin A | Lipid Pos | 81.96 | C40 H56 O3 | 0.690 | HMDB35319 |
| Cyanophos | Lipid Pos | 86.59 | C9 H10 N O3 P S | 1.355 | KEGG: C18397 |
| Cyclopassifloside V | Lipid Pos | 78.33 | C43 H72 O17 | 7.255 | HMDB35947 |
| Darifenacin | Lipid Neg | 78.03 | C28 H30 N2 O2 | 3.725 | HMDB14639 |
| Deacetyl-O-demethyldiltiazem | Lipid Pos | 73.71 | C19 H22 N2 O3 S | 1.354 | CAS: 84903-82-2 |
| Decanamide | Lipid Pos | 87.93 | C10 H21 N O | 1.068 | LMFA08010005 |
| Decanoylcholine | Lipid Neg | 97.91 | C15 H32 N O2 | 3.614 | HMDB13228 |
| Decaprenoxanthin | Lipid Pos | 84.76 | C50 H72 | 2.952 | LMPR01080008 |
| Decylubiquinol | Lipid Pos | 74.33 | C19 H32 O4 | 1.529 | KEGG: C15495 |
| Dehydrocarpaine II | Lipid Pos | 70.26 | C28 H46 N2 O4 | 3.732 | HMDB30273 |
| Dehydroisochalciporone | Lipid Pos | 78.94 | C16 H19 N O | 1.147 | HMDB33527 |
| Dehydrophytosphingosine | Lipid Pos | 84.24 | C18 H37 N O3 | 2.444 | KEGG: C00836 |
| Deoxodeoxydihydrogedunin | Lipid Pos | 93.66 | C28 H38 O5 | 3.172 | PubChem: 6708592 |
| Deoxyguanosine | Aqueous | 99.40 | C10 H13 N5 O4 | 3.998 | KEGG: C00330 |
| Deoxymiroestrol | Lipid Pos | 99.05 | C20 H22 O5 | 1.220 | KEGG: C18164 |
| Deoxyribose 5-phosphate | Lipid Neg | 82.01 | C5 H11 O7 P | 0.787 | KEGG: C00673 |
| Desoxycorticosterone acetate | Lipid Pos | 73.13 | C23 H32 O4 | 1.032 | KEGG: C14554 |
| Deterrol stearate | Lipid Pos | 89.30 | C33 H50 O2 | 2.158 | HMDB34578 |
| DG(14:0/20:0/0:0) | Lipid Pos | 96.13 | C37 H72 O5 | 7.141 | KEGG: C00641 |
| DG(14:1(9Z)/22:1(13Z)/0:0) | Lipid Pos | 82.22 | C39 H72 O5 | 2.467 | HMDB07058 |
| DG(15:0/0:0/20:0) | Lipid Pos | 98.12 | C38 H74 O5 | 3.283 | KEGG: C00641 |
| DG(15:0/15:0/0:0) | Lipid Pos | 94.04 | C33 H64 O5 | 5.094 | HMDB07068 |
| DG(15:0/18:2(9Z,12Z)/0:0) | Lipid Pos | 90.92 | C36 H66 O5 | 1.353 | KEGG: C00641 |
| DG(16:0/16:0/0:0) | Lipid Pos | 96.23 | C35 H68 O5 | 6.711 | KEGG: C00165 |
| DG(16:0/18:0/0:0) | Lipid Pos | 81.03 | C37 H72 O5 | 7.142 | KEGG: C00641 |
| DG(16:0/18:1)* | Lipid | 96.05 | C37 H70 O5 | 6.742 | LMGL02010307 |
| DG(16:0/20:4(8Z,11Z,14Z,17Z)/0:0) | Lipid Pos | 90.15 | C39 H68 O5 | 6.742 | KEGG: C00641 |
| DG(17:0/18:0/0:0) | Lipid Neg | 85.80 | C38 H74 O5 | 7.075 | LMGL02010025 |
| DG(18:0/0:0/18:0) | Lipid Pos | 85.16 | C39 H76 O5 | 7.516 | KEGG: C00641 |
| DG(18:0/15:0/0:0) | Lipid Pos | 78.14 | C36 H70 O5 | 6.951 | KEGG: C00641 |
| DG(18:0/16:0/0:0) | Lipid Pos | 95.23 | C37 H72 O5 | 7.141 | KEGG: C00641 |
| DG(18:3/18:4/0:0) | Lipid Pos | 85.28 | C39 H62 O5 | 2.013 | KEGG: C00641 |
| DG(18:4/18:3/0:0) | Lipid Pos | 72.25 | C39 H62 O5 | 5.756 | HMDB07336 |
| DG(20:0/15:0/0:0) | Lipid Pos | 91.83 | C38 H74 O5 | 7.342 | KEGG: C00641 |
| DG(20:0/16:0/0:0) | Lipid Pos | 99.31 | C39 H76 O5 | 7.516 | KEGG: C00641 |
| DG(20:2(11Z,14Z)/16:0/0:0) | Lipid Pos | 77.88 | C39 H72 O5 | 2.466 | HMDB07417 |
| DG(20:3(5Z,8Z,11Z)/16:1(9Z)/0:0) | Lipid Pos | 85.71 | C39 H68 O5 | 6.316 | HMDB07447 |
| DG(20:3(8Z,11Z,14Z)/18:1(11Z)/0:0) | Lipid Pos | 91.89 | C41 H72 O5 | 6.766 | HMDB07478 |
| DG(24:0/0:0/22:5n3) | Lipid Pos | 91.91 | C49 H86 O5 | 3.722 | KEGG: C00641 |
| DG(24:0/22:5(4Z,7Z,10Z,13Z,16Z)/0:0) | Lipid Pos | 92.59 | C49 H86 O5 | 3.722 | KEGG: C00641 |
| DG(24:1(15Z)/16:1(9Z)/0:0) | Lipid Pos | 86.47 | C43 H80 O5 | 3.182 | KEGG: C00641 |
| DG(24:1(15Z)/24:1(15Z)/0:0) | Lipid Pos | 92.80 | C51 H96 O5 | 8.245 | KEGG: C00641 |
| Dibutyl adipate | Lipid Pos | 91.40 | C14 H26 O4 | 2.041 | KEGG: C14253 |
| Diethylene glycol distearate | Lipid Neg | 84.13 | C40 H78 O5 | 7.556 | HMDB36223 |
| Diethylphosphate | Lipid Pos | 93.85 | C4 H11 O4 P | 1.312 | KEGG: C06608 |
| Dihydro-2,4-dimethyl-6-(1-methylpropyl)-4H-1,3,5-dithiazine | Lipid Pos | 80.59 | C9 H19 N S2 | 1.031 | HMDB40336 |
| Dihydrozeatin | Lipid Pos | 92.40 | C10 H15 N5 O | 1.113 | KEGG: C02029 |
| Diisooctyl phthalate | Lipid Pos | 77.19 | C24 H38 O4 | 3.173 | PubChem: 33934 |
| Dinor-PGD2 | Lipid Pos | 77.46 | C18 H28 O5 | 1.358 | LMFA03010214 |
| D-NMAPPD | Lipid Pos | 85.46 | C23 H38 N2 O5 | 0.598 | CAS: 35922-06-6 |
| Docosanamide | Lipid Pos | 92.30 | C22 H45 N O | 4.467 | HMDB00583 |
| Dodecyl propionate | Lipid Neg | 99.97 | C15 H30 O2 | 2.873 | HMDB32250 |
| Dolichotheline | Lipid Pos | 74.58 | C10 H17 N3 O | 2.520 | KEGG: C16696 |
| Dolichotheline | Lipid Pos | 89.11 | C10 H17 N3 O | 2.519 | KEGG: C16696 |
| dolichyl-4 phosphate | Lipid Pos | 70.35 | C25 H45 O4 P | 1.548 | KEGG: C00110 |
| Dorsteniol | Lipid Pos | 71.75 | C14 H14 O5 | 0.936 | HMDB32838 |
| Dysideapalaunic acid | Lipid Pos | 77.03 | C25 H40 O2 | 3.189 | LMPR0105040001 |
| Ecabet | Lipid Neg | 93.36 | C20 H28 O5 S | 2.079 | HMDB15613 |
| Epiafzelechin Trimethyl Ether | Lipid Pos | 80.19 | C18 H20 O5 | 0.982 | PubChem: 6708630 |
| Epipregnanolone | Lipid Neg | 80.85 | C21 H34 O2 | 3.736 | KEGG: C11825 |
| Ergosta-5,7,22,24(28)-tetraen-3β-ol | Lipid Pos | 79.01 | C28 H42 O | 1.960 | KEGG: C05440 |
| erythro-6,8-Heneicosanediol | Lipid Pos | 91.78 | C21 H44 O2 | 5.835 | HMDB41077 |
| erythro-6,8-Heptacosanediol | Lipid Pos | 93.39 | C27 H56 O2 | 7.623 | HMDB41071 |
| erythro-6,8-Pentacosanediol | Lipid Pos | 90.63 | C25 H52 O2 | 7.147 | HMDB41075 |
| erythro-6,8-Tricosanediol | Lipid Pos | 90.08 | C23 H48 O2 | 6.825 | HMDB41070 |
| erythro-7,9-Octacosanediol | Lipid Pos | 95.91 | C28 H58 O2 | 7.939 | HMDB31286 |
| erythro-7,9-Tetratriacontanediol | Lipid Pos | 85.64 | C34 H70 O2 | 9.606 | HMDB31281 |
| erythro-7,9-Triacontanediol | Lipid Pos | 75.95 | C30 H62 O2 | 8.452 | HMDB31285 |
| erythro-8,10-Heptacosanediol | Lipid Pos | 94.13 | C27 H56 O2 | 7.625 | HMDB31278 |
| Estradiol-17α | Lipid Pos | 90.69 | C18 H24 O2 | 1.407 | KEGG: C02537 |
| Ethohexadiol | Lipid Pos | 84.47 | C8 H18 O2 | 0.790 | KEGG: C14271 |
| Ethyl 3-hydroxydodecanoate | Lipid Pos | 93.93 | C14 H28 O3 | 1.142 | HMDB59850 |
| Ethyl hexadecanoate | Lipid Neg | 98.55 | C18 H36 O2 | 4.019 | HMDB29811 |
| FA unsat C19:1 (cis-10-nonadecenoic acid) | Lipid Pos | 92.70 | C19 H36 O2 | 3.291 | CAS: 73033-97-7 |
| Falcarindione | Lipid Neg | 85.77 | C17 H20 O2 | 1.499 | HMDB33680 |
| Filfiline | Lipid Pos | 81.40 | C26 H47 N O | 4.833 | HMDB30953 |
| Flaccidin B | Lipid Pos | 80.91 | C41 H64 O12 | 6.159 | KEGG: C08943 |
| Floionolic acid | Lipid Neg | 99.98 | C18 H36 O5 | 1.275 | KEGG: C19621 |
| Fragransin B2 | Lipid Pos | 80.90 | C22 H28 O7 | 0.965 | HMDB38953 |
| Ganoderol A | Lipid Pos | 78.80 | C30 H46 O2 | 1.873 | HMDB35728 |
| Gibberellin A8 | Lipid Pos | 77.44 | C19 H24 O7 | 1.356 | KEGG: C03579 |
| Gingerol | Lipid Neg | 99.11 | C17 H26 O4 | 1.350 | KEGG: C10462 |
| Glu Ile Lys | Aqueous | 92.22 | C17 H32 N4 O6 | 7.172 | Metlin: 16755 |
| Glutaminyl-Glutamate | Aqueous | 92.04 | C10 H16 N3 O6 | 6.796 | HMDB28796 |
| Glutethimide | Lipid Pos | 76.85 | C13 H15 N O2 | 1.198 | KEGG: C07489 |
| Glycerol 1,3-dihexadecanoate | Lipid Pos | 96.01 | C35 H68 O5 | 6.719 | HMDB31011 |
| Glycerol 1-stearate* | Lipid Pos | 94.12 | C21 H42 O4 | 2.920 | CAS: 123-94-4 |
| Glyceryl 1-mononitrate | Lipid Pos | 70.72 | C3 H7 N O5 | 10.526 | CAS: 624-43-1 |
| Glycerylphosphorylethanolamine | Lipid Pos | 73.23 | C5 H14 N O6 P | 10.692 | KEGG: C01233 |
| Gossypetin 3-O-sulfate | Lipid Neg | 79.03 | C15 H10 O11 S | 1.675 | LMPK12113267 |
| Guanadrel Sulfate | Lipid Pos | 70.73 | C10 H19 N3 O2 | 2.047 | KEGG: C07035 |
| Guanadrel Sulfate | Lipid Pos | 94.82 | C10 H19 N3 O2 | 2.914 | KEGG: C07035 |
| Heptadecan-2-ol | Lipid Pos | 95.47 | C17 H36 O | 1.580 | LMFA05000531 |
| Heptanoylglycine | Aqueous | 83.09 | C9 H17 N O3 | 7.667 | HMDB13010 |
| Hericene A | Lipid Pos | 78.09 | C35 H56 O5 | 1.613 | HMDB41179 |
| Hericene B | Lipid Pos | 90.98 | C37 H58 O5 | 1.586 | HMDB41180 |
| Hexadecasphinganine | Lipid Pos | 79.96 | C16 H35 N O2 | 1.310 | LMSP01040001 |
| Hexanal hexyl isoamyl acetal | Lipid Pos | 92.61 | C17 H36 O2 | 3.266 | HMDB32317 |
| Homocysteic acid | Aqueous | 74.05 | C4 H9 N O5 S | 1.772 | KEGG: C16511 |
| Homodihydrocapsaicin | Lipid Neg | 80.29 | C19 H31 N O3 | 3.725 | HMDB36330 |
| Hydroxypropionic acid | Lipid Neg | 87.73 | C3 H6 O3 | 0.414 | KEGG: C01013 |
| Hyoscyamine | Lipid Pos | 78.67 | C17 H23 N O3 | 1.307 | KEGG: C02046 |
| Hypoxanthine* | Aqueous | 86.07 | C5 H4 N4 O | 2.972 | KEGG: C00262 |
| Idoxuridine | Lipid Neg | 92.60 | C9 H11 I N2 O5 | 1.675 | HMDB14394 |
| Indoleacrylic acid | Aqueous | 86.40 | C11 H9 N O2 | 4.592 | HMDB00734 |
| Isoamoritin | Lipid Pos | 73.30 | C31 H38 O6 | 1.783 | LMPK12140384 |
| Isobutanol | Lipid Pos | 82.10 | C4 H10 O | 0.362 | KEGG: C14710 |
| Isoderricin A | Lipid Pos | 75.37 | C21 H22 O3 | 0.375 | LMPK12140014 |
| Isoleucine | Lipid Pos | 97.96 | C6 H13 N O2 | 0.514 | KEGG: C16434 |
| Isopentanol | Lipid Pos | 76.04 | C5 H12 O | 1.029 | KEGG: C07328 |
| Isopropyl 2-methylbutanoate | Lipid Neg | 87.50 | C8 H16 O2 | 1.279 | HMDB39217 |
| Isopropyl β-D-glucoside | Aqueous | 78.67 | C9 H18 O6 | 6.920 | HMDB32705 |
| Kalkitoxin thioamide alcohol | Lipid Pos | 82.73 | C21 H40 N2 O2 S | 2.535 |  |
| k-Strophanthoside | Lipid Pos | 77.90 | C42 H64 O19 | 1.354 | KEGG: C08881 |
| LacCeramide (d18:1/14:0) | Lipid Pos | 79.94 | C44 H83 N O13 | 6.045 | LMSP0501AB12 |
| L-Acetylcarnitine | Lipid Neg | 81.22 | C9 H17 N O4 | 3.616 | KEGG: C02571 |
| Lactaroviolin | Lipid Pos | 74.38 | C15 H14 O | 1.312 | KEGG: C09696 |
| Lepidine C | Lipid Pos | 86.74 | C21 H20 N4 O2 | 1.216 | HMDB32717 |
| Lepidine C | Lipid Pos | 85.32 | C21 H20 N4 O2 | 1.216 | HMDB32717 |
| Leu Val Ile | Lipid Pos | 73.25 | C17 H33 N3 O4 | 1.312 |  |
| Leucine* | Aqueous | 98.47 | C6 H13 N O2 | 1.476 | KEGG: C00123 |
| L-Histidinol | Lipid Pos | 82.34 | C6 H11 N3 O | 10.432 | KEGG: C00860 |
| Linoleyl alcohol* | Lipid Pos | 89.22 | C18 H34 O | 2.921 | CAS: 1577-52-2 |
| Lipid A -disaccharide-1-P | Lipid Pos | 78.08 | C68 H129 N2 O20 P | 6.156 | LMSL01040002 |
| Lisuride | Lipid Pos | 72.64 | C20 H26 N4 O | 2.468 | HMDB14727 |
| L-Pimaric acid | Lipid Pos | 73.59 | C20 H30 O2 | 2.242 | KEGG: C11888 |
| Luteone (isoflavone) | Lipid Pos | 73.63 | C20 H18 O6 | 1.058 | LMPK12050287 |
| Lys Ala Lys | Lipid Pos | 83.14 | C15 H31 N5 O4 | 6.160 |  |
| Lys Gln Leu | Lipid Pos | 86.51 | C17 H33 N5 O5 | 1.000 |  |
| LysoPC(16:0)* | Lipid Pos | 97.02 | C24 H50 N O7 P | 1.598 | KEGG: C04230 |
| LysoPC(16:1(9Z)) | Lipid Pos | 84.37 | C24 H48 N O7 P | 1.356 | KEGG: C04230 |
| LysoPC(18:0)* | Lipid Pos | 98.22 | C26 H54 N O7 P | 2.052 | LMGP02050026 |
| LysoPC(18:1)* | Lipid Pos | 85.73 | C26 H52 N O7 P | 1.676 | KEGG: C04230 |
| LysoPC(18:2(9Z,12Z)) | Lipid Pos | 96.19 | C26 H50 N O7 P | 1.443 | KEGG: C04230 |
| LysoPC(18:3(6Z,9Z,12Z)) | Lipid Pos | 84.82 | C26 H49 N O7 P | 1.600 | KEGG: C04230 |
| LysoPC(20:4(5Z,8Z,11Z,14Z)) | Lipid Pos | 84.37 | C28 H50 N O7 P | 1.397 | KEGG: C04230 |
| LysoPC(20:4(8Z,11Z,14Z,17Z)) | Lipid Pos | 86.24 | C28 H50 N O7 P | 1.416 | KEGG: C04230 |
| LysoPC(20:5(5Z,8Z,11Z,14Z,17Z)) | Lipid Pos | 89.57 | C28 H48 N O7 P | 1.438 | KEGG: C04230 |
| LysoPE(0:0/16:0) | Lipid Pos | 82.77 | C21 H44 N O7 P | 1.624 | KEGG: C06254 |
| LysoPE(18:0)* | Lipid Pos | 94.05 | C23 H48 N O7 P | 2.076 | PubChem: 9547069 |
| LysoPE(18:0/0:0) | Lipid Neg | 98.96 | C23 H48 N O7 P | 1.959 | HMDB11130 |
| LysoPE(20:4(5Z,8Z,11Z,14Z)/0:0) | Lipid Pos | 73.45 | C25 H44 N O7 P | 1.435 | KEGG: C06254 |
| L-α-Acetyl-N,N-dinormethadol | Lipid Pos | 80.06 | C21 H27 N O2 | 1.312 | KEGG: C16662 |
| L-α-Amino-1H-pyrrole-1-hexanoic acid | Lipid Neg | 99.77 | C10 H16 N2 O2 | 1.266 | HMDB40551 |
| Mahuannin D | Lipid Pos | 74.07 | C30 H24 O9 | 7.816 | KEGG: C10234 |
| Malaoxon | Lipid Neg | 70.90 | C10 H19 O7 P S | 1.675 | HMDB60627 |
| m-Cresol | Lipid Pos | 83.58 | C7 H8 O | 1.277 | KEGG: C01467 |
| Menthone lactone | Lipid Pos | 90.47 | C10 H18 O2 | 1.142 | KEGG: C18066 |
| Methyl (7Z,9Z,9'Z)-6'-apo-y-caroten-6'-oate | Lipid Pos | 79.89 | C33 H44 O2 | 1.094 | HMDB31381 |
| methyl 15-hydroperoxy-9Z,12Z,16E-octadecatrienoate | Lipid Pos | 82.97 | C19 H32 O4 | 2.060 | LMFA01040039 |
| Methyl 3b-hydroxy-13(18)-oleanen-28-oate | Lipid Pos | 79.61 | C33 H52 O4 | 1.644 | HMDB35267 |
| Methyleugenol | Lipid Pos | 86.21 | C11 H14 O2 | 1.242 | KEGG: C10454 |
| MG(0:0/18:0/0:0) | Lipid Pos | 83.87 | C21 H42 O4 | 2.921 | KEGG: C01885 |
| MG(0:0/18:2(9Z,12Z)/0:0) | Lipid Pos | 72.69 | C21 H38 O4 | 1.984 | KEGG: C01885 |
| MG(0:0/18:3(9Z,12Z,15Z)/0:0) | Lipid Pos | 75.74 | C21 H36 O4 | 1.605 | KEGG: C01885 |
| MG(16:0/0:0/0:0) | Lipid Neg | 83.90 | C19 H38 O4 | 2.673 | HMDB11564 |
| MG(16:0/0:0/0:0) | Lipid Pos | 93.56 | C19 H38 O4 | 2.263 | KEGG: C01885 |
| MG(18:0/0:0/0:0) | Lipid Neg | 99.27 | C21 H42 O4 | 3.358 | HMDB11131 |
| MG(18:0/0:0/0:0) | Lipid Pos | 98.72 | C21 H42 O4 | 2.921 | KEGG: C01885 |
| MG(18:0e/0:0/0:0) | Lipid Pos | 71.28 | C21 H44 O3 | 3.574 | KEGG: C01885 |
| MG(24:0/0:0/0:0) | Lipid Pos | 77.05 | C27 H54 O4 | 4.546 | HMDB11588 |
| Monoisobutyl phthalic acid | Lipid Neg | 84.53 | C12 H14 O4 | 1.098 | HMDB02056 |
| Morpholine | Lipid Pos | 75.93 | C4 H9 N O | 10.466 | KEGG: C14452 |
| Myosmine | Lipid Pos | 70.22 | C9 H10 N2 | 1.354 | KEGG: C10160 |
| Myrtenol | Lipid Pos | 77.78 | C10 H16 O | 1.144 | KEGG: C11938 |
| N-(11Z-eicosaenoyl)-ethanolamine | Lipid Pos | 98.75 | C22 H43 N O2 | 3.153 | LMFA08040010 |
| N-(2R-hydroxy-15-methyl-3E-octadecenoyl)-4E,10E,12E-sphingatrienine | Lipid Neg | 85.99 | C37 H67 N O4 | 6.511 | LMSP02010095 |
| N-(3-(hexadecanoyloxy)-heptadecanoyl)-L-ornithine | Lipid Pos | 81.80 | C38 H74 N2 O5 | 6.807 | LMFA00000003 |
| N-(6-aminohexanoyl)-6-aminohexanoic acid | Lipid Pos | 80.89 | C12 H24 N2 O3 | 0.513 | KEGG: C01255 |
| N-(8Z,11Z,14Z-eicosatrienoyl)-ethanolamine | Lipid Pos | 92.62 | C22 H39 N O2 | 7.178 | LMFA08040011 |
| N-(tetradecanoyl)-deoxysphing-4-enine-1-sulfonate | Lipid Pos | 79.52 | C32 H63 N O5 S | 1.613 | LMSP00000001 |
| N,2,3-Trimethyl-2-(1-methylethyl)butanamide | Lipid Pos | 85.65 | C10 H21 N O | 1.736 | HMDB36195 |
| N,N-Diethylglycine | Lipid Pos | 98.62 | C6 H13 N O2 | 0.514 | KEGG: C16647 |
| N,N-dimethyl-Safingol | Lipid Pos | 82.39 | C20 H43 N O2 | 3.648 | LMSP01080056 |
| N1-(2-Methoxy-4-methylbenzyl)-n2-(2-(5-methylpyridin-2-yl)ethyl)oxalamide | Lipid Pos | 84.13 | C19 H23 N3 O3 | 0.818 | HMDB32382 |
| N3-Metyladenine | Lipid Pos | 80.92 | C6 H9 N5 | 0.790 | KEGG: C05026 |
| N6,N6,N6-Trimethyl-L-lysine | Lipid Pos | 80.95 | C9 H20 N2 O2 | 0.789 | KEGG: C03793 |
| N-Acetyl-b-glucosaminylamine | Lipid Neg | 78.58 | C8 H16 N2 O5 | 3.760 | KEGG: C01239 |
| N-cis-tetradec-9Z-enoyl-L-Homoserine lactone | Lipid Pos | 73.29 | C18 H31 N O3 | 1.312 | PubChem: 35028743 |
| N-Desthienylethyl-rotigotine | Lipid Neg | 99.56 | C13 H19 N O | 1.815 | HMDB60843 |
| N-eicosanoyl-ethanolamine | Lipid Pos | 98.99 | C22 H45 N O2 | 3.723 | LMFA08040038 |
| Nervonoyl-ethanolamine | Lipid Neg | 99.61 | C26 H51 N O2 | 3.728 | LMFA08040055 |
| Neurine | Aqueous | 86.80 | C5 H13 N O | 1.135 | HMDB31259 |
| Neuromedin B (1-3) | Lipid Neg | 74.94 | C12 H22 N4 O5 | 1.355 | HMDB13016 |
| N-Heptanoylglycine | Lipid Neg | 81.51 | C9 H17 N O3 | 4.019 | HMDB13010 |
| N-Lactoyl ethanolamine | Lipid Pos | 72.78 | C5 H11 N O3 | 0.667 | HMDB32356 |
| N-Nitrososarcosine | Lipid Pos | 93.59 | C3 H6 N2 O3 | 0.515 | KEGG: C19286 |
| N-octadecanoyl-valine | Lipid Neg | 99.87 | C23 H45 N O3 | 4.166 | LMFA08020122 |
| N-octanoyl-L-Homoserine lactone | Lipid Pos | 70.75 | C12 H21 N O3 | 4.044 | CAS: 147852-84-4 |
| Nonane | Lipid Pos | 77.09 | C9 H20 | 1.142 | KEGG: C02445 |
| Norajmaline | Lipid Pos | 74.86 | C20 H25 N O2 | 1.122 | KEGG: C11810 |
| N-palmitoyl leucine | Lipid Pos | 85.58 | C22 H43 N O3 | 1.900 | LMFA08020115 |
| N-Undecanoylglycine | Lipid Neg | 99.84 | C13 H25 N O3 | 1.047 | HMDB13286 |
| N-Undecylbenzenesulfonic acid | Lipid Neg | 97.78 | C17 H28 O3 S | 2.080 | HMDB32549 |
| O-acetyl-ADP-ribose | Lipid Pos | 72.45 | C17 H25 N5 O15 P2 | 1.029 | HMDB59656 |
| Obacunone | Lipid Pos | 72.52 | C26 H30 O7 | 1.053 | KEGG: C08775 |
| Octadecyl fumarate | Lipid Pos | 77.16 | C22 H40 O4 | 2.329 | HMDB38073 |
| Octanal | Lipid Pos | 79.27 | C8 H16 O | 0.790 | KEGG: C01545 |
| Octyl 2-methyl-propionyl | Lipid Neg | 86.00 | C12 H24 O2 | 2.018 | LMFA07010504 |
| Octylamine | Lipid Pos | 92.84 | C8 H19 N | 0.439 | KEGG: C01740 |
| Oleoyl ethylamide* | Lipid Pos | 93.49 | C20 H39 N O | 2.966 | CAS: 85075-82-7 |
| O-octadecanoyl-R-carnitine | Lipid Neg | 98.58 | C25 H49 N O4 | 3.614 | LMFA07070008 |
| O-octanoyl-R-carnitine | Lipid Neg | 98.50 | C15 H29 N O4 | 2.878 | KEGG: C02838 |
| Oseltamivir | Lipid Neg | 91.84 | C16 H28 N2 O4 | 2.216 | KEGG: C08092 |
| Oxidized glutathione* | Aqueous | 97.99 | C20 H32 N6 O12 S2 | 1.751 | KEGG: C00127 |
| Oxprenolol | Lipid Neg | 85.89 | C15 H23 N O3 | 2.023 | HMDB15520 |
| PA(14:0/14:1(9Z)) | Lipid Pos | 76.34 | C31 H59 O8 P | 1.470 | KEGG: C00416 |
| PA(14:1(9Z)/16:1(9Z)) | Lipid Pos | 73.78 | C33 H61 O8 P | 1.447 | KEGG: C00416 |
| PA(14:1(9Z)/20:5(5Z,8Z,11Z,14Z,17Z)) | Lipid Pos | 85.94 | C37 H61 O8 P | 1.337 | KEGG: C00416 |
| PA(16:1(9Z)/18:0) | Lipid Neg | 97.48 | C37 H71 O8 P | 1.242 | LMGP10010209 |
| PA(17:1(9Z)/22:6(4Z,7Z,10Z,13Z,16Z,19Z)) | Lipid Pos | 78.51 | C42 H69 O8 P | 6.159 | KEGG: C00416 |
| PA(20:3(8Z,11Z,14Z)/22:2(13Z,16Z)) | Lipid Pos | 73.72 | C45 H79 O8 P | 2.932 | LMGP10010615 |
| PA(22:0/18:0) | Lipid Pos | 74.41 | C43 H85 O8 P | 3.721 | LMGP10010846 |
| PA(22:2(13Z,16Z)/22:0) | Lipid Pos | 87.89 | C47 H89 O8 P | 7.835 | LMGP10010781 |
| PA(22:6/22:4) | Lipid Pos | 78.76 | C47 H73 O8 P | 1.000 | KEGG: C00416 |
| Palmitoylglycine | Lipid Pos | 70.10 | C18 H35 N O3 | 1.531 | HMDB13034 |
| Palmityl Trifluoromethyl Ketone | Lipid Pos | 97.49 | C17 H31 F3 O | 1.029 | CAS: 141022-99-3 |
| Panaxatriol | Lipid Pos | 81.83 | C30 H52 O4 | 4.559 | HMDB38244 |
| Panaxydol linoleate | Lipid Pos | 77.11 | C35 H54 O3 | 2.098 | HMDB41205 |
| Pangamic acid | Lipid Pos | 84.73 | C20 H40 N2 O8 | 1.575 | HMDB29949 |
| Pantetheine | Lipid Pos | 91.42 | C11 H22 N2 O4 S | 0.382 | KEGG: C00831 |
| PC(14:0/18:1(11Z)) | Lipid Neg | 98.66 | C40 H78 N O8 P | 5.733 | HMDB07872 |
| PC(14:1(9Z)/20:1(11Z)) | Lipid Neg | 99.36 | C42 H80 N O8 P | 5.876 | LMGP01011398 |
| PC(15:1(9Z)/18:2(9Z,12Z)) | Lipid Neg | 88.49 | C41 H76 N O8 P | 5.892 | LMGP01011444 |
| PC(16:1(9Z)/17:1(9Z)) | Lipid Neg | 99.00 | C41 H78 N O8 P | 6.398 | LMGP01011481 |
| PC(16:1(9Z)/20:1(11Z)) | Lipid Pos | 77.29 | C44 H84 N O8 P | 5.570 | LMGP01011489 |
| PC(17:0/17:1(9Z)) | Lipid Neg | 99.04 | C42 H82 N O8 P | 6.429 | LMGP01011503 |
| PC(18:0/14:0) 6.342995 | Lipid Neg | 99.18 | C40 H80 N O8 P | 6.343 | KEGG: C00157 |
| PC(18:1(11Z)/18:1(11Z)) | Lipid Neg | 98.00 | C44 H84 N O8 P | 6.635 | HMDB08070 |
| PC(18:3(6Z,9Z,12Z)/18:1(9Z)) | Lipid Neg | 99.28 | C44 H80 N O8 P | 5.741 | LMGP01011652 |
| PC(18:4(6Z,9Z,12Z,15Z)/17:0) | Lipid Neg | 94.51 | C43 H78 N O8 P | 6.163 | LMGP01011708 |
| PC(18:4/22:6) | Lipid Pos | 72.78 | C48 H76 N O8 P | 4.682 | KEGG: C00157 |
| PC(20:5(5Z,8Z,11Z,14Z,17Z)/15:1(9Z)) | Lipid Neg | 99.48 | C43 H74 N O8 P | 5.370 | LMGP01011931 |
| PC(20:5(5Z,8Z,11Z,14Z,17Z)/15:1(9Z)) | Lipid Neg | 99.09 | C43 H74 N O8 P | 5.581 | LMGP01011931 |
| PC(O-12:0/O-12:0) | Lipid Pos | 82.33 | C32 H68 N O6 P | 5.754 | LMGP01040015 |
| PC(O-12:0/O-2:0) | Lipid Pos | 79.86 | C22 H48 N O6 P | 1.000 | LMGP01040017 |
| PC(P-18:1(9Z)/22:2(13Z,16Z)) | Lipid Pos | 70.30 | C48 H90 N O7 P | 2.467 | HMDB11324 |
| PE(12:0/18:0) | Lipid Neg | 98.99 | C35 H70 N O8 P | 5.540 | LMGP02011261 |
| PE(14:0/16:0) | Lipid Pos | 98.09 | C35 H70 N O8 P | 5.077 | KEGG: C06254 |
| PE(14:0/17:0) | Lipid Neg | 97.71 | C36 H72 N O8 P | 4.817 | LMGP02010409 |
| PE(16:1(9Z)/18:0) | Lipid Neg | 98.80 | C39 H76 N O8 P | 6.253 | LMGP02010524 |
| PE(16:1(9Z)/20:4(5Z,8Z,11Z,14Z)) | Lipid Pos | 78.77 | C41 H72 N O8 P | 5.355 | LMGP02011217 |
| PE(17:0/17:2(9Z,12Z)) | Lipid Neg | 96.08 | C39 H74 N O8 P | 5.791 | LMGP02010545 |
| PE(18:0/20:4(5Z,8Z,11Z,13E)(15Ke)) | Lipid Pos | 98.71 | C46 H82 N O9 P | 5.296 | LMGP20010004 |
| PE(18:0/20:4(5Z,8Z,11Z,13E)(15OH[S])) | Lipid Pos | 96.94 | C43 H78 N O9 P | 5.352 | LMGP20020002 |
| PE(18:3(6Z,9Z,12Z)/14:0) | Lipid Pos | 88.36 | C37 H68 N O8 P | 5.080 | KEGG: C06254 |
| PE(18:3(6Z,9Z,12Z)/14:1(9Z)) | Lipid Pos | 75.29 | C37 H66 N O8 P | 2.829 | KEGG: C00350 |
| PE(20:3(5Z,8Z,11Z)/18:2(9Z,12Z)) | Lipid Pos | 90.19 | C43 H76 N O8 P | 5.380 | KEGG: C00350 |
| PE(20:3(8Z,11Z,14Z)/P-16:0) | Lipid Neg | 98.49 | C41 H76 N O7 P | 6.165 | KEGG: C00350 |
| PE(20:3(8Z,11Z,14Z)/P-18:1(9Z)) | Lipid Neg | 94.92 | C43 H78 N O7 P | 6.593 | KEGG: C00350 |
| PE(20:4(8Z,11Z,14Z,17Z)/16:0) | Lipid Neg | 98.53 | C41 H74 N O8 P | 5.719 | KEGG: C00350 |
| PE(20:4(8Z,11Z,14Z,17Z)/18:1(11Z)) | Lipid Neg | 99.02 | C43 H76 N O8 P | 5.802 | KEGG: C00350 |
| PE(20:4/22:4) | Lipid Pos | 82.57 | C47 H78 N O8 P | 5.975 | KEGG: C00350 |
| PE(20:5/20:2) | Lipid Neg | 96.51 | C45 H76 N O8 P | 5.661 | KEGG: C00350 |
| PE(22:2(13Z,16Z)/14:1(9Z)) | Lipid Pos | 99.38 | C41 H76 N O8 P | 5.561 | KEGG: C00350 |
| PE(22:4(7Z,10Z,13Z,16Z)/18:1(11Z)) | Lipid Pos | 81.74 | C45 H80 N O8 P | 5.958 | KEGG: C00350 |
| PE(22:4(7Z,10Z,13Z,16Z)/P-16:0) | Lipid Neg | 98.79 | C43 H78 N O7 P | 6.588 | KEGG: C00350 |
| PE(22:5(7Z,10Z,13Z,16Z,19Z)/dm18:1(9Z)) | Lipid Neg | 97.08 | C45 H78 N O7 P | 6.446 | KEGG: C00350 |
| PE(O-16:0/15:1(9Z)) | Lipid Neg | 98.65 | C36 H72 N O7 P | 5.534 | LMGP02020026 |
| PE(P-16:0/20:4(8Z,11Z,14Z,17Z)) | Lipid Neg | 98.25 | C41 H74 N O7 P | 6.016 | HMDB11353 |
| PE(P-18:1(11Z)/16:1(9Z)) | Lipid Neg | 98.50 | C39 H74 N O7 P | 6.109 | HMDB11405 |
| PE(P-18:1(11Z)/20:5(5Z,8Z,11Z,14Z,17Z)) | Lipid Neg | 98.82 | C43 H74 N O7 P | 5.866 | HMDB11420 |
| PE(P-18:1(9Z)/20:4(8Z,11Z,14Z,17Z)) | Lipid Neg | 99.69 | C43 H76 N O7 P | 6.080 | HMDB11452 |
| PE(P-18:1(9Z)/20:4(8Z,11Z,14Z,17Z)) | Lipid Neg | 99.68 | C43 H76 N O7 P | 6.230 | HMDB11452 |
| PE-Ceramide (d14:1(4E)/24:0(2OH)) | Lipid Pos | 70.03 | C40 H81 N2 O7 P | 4.950 | LMSP03020064 |
| PE-Ceramide (d14:2(4E,6E)/20:1(2OH)) | Lipid Neg | 70.94 | C36 H69 N2 O7 P | 3.726 | LMSP03020069 |
| PE-Ceramide (d14:2(4E,6E)/20:1(11Z)) | Lipid Pos | 91.14 | C36 H69 N2 O6 P | 1.032 | LMSP03020033 |
| PE-Ceramide (d15:1(4E)/22:0(2OH)) | Lipid Pos | 81.58 | C39 H79 N2 O7 P | 4.366 | LMSP03020075 |
| PE-Ceramide (d16:1(4E)/21:0) | Lipid Neg | 99.36 | C39 H79 N2 O6 P | 5.276 | LMSP03020023 |
| Pelargonic acid | Lipid Pos | 73.73 | C9 H18 O2 | 1.190 | KEGG: C01601 |
| Peltatol A | Lipid Pos | 73.96 | C42 H58 O4 | 1.029 | HMDB38701 |
| Pentadecylic acid | Lipid Pos | 85.36 | C15 H30 O2 | 2.459 | KEGG: C16537 |
| Pentosidine | Lipid Neg | 84.03 | C17 H26 N6 O4 | 2.882 | HMDB03933 |
| Perflutren | Lipid Pos | 81.88 | C3 F8 | 10.455 | HMDB14696 |
| PG(12:0/22:2(13Z,16Z)) | Lipid Neg | 98.45 | C40 H75 O10 P | 6.404 | LMGP04010066 |
| PG(14:0/15:1(9Z)) | Lipid Pos | 73.71 | C35 H67 O10 P | 1.427 | KEGG: C00344 |
| PG(14:0/18:0) | Lipid Pos | 88.27 | C38 H75 O10 P | 6.154 | LMGP04010929 |
| PG(16:0/20:2(11Z,14Z)) | Lipid Neg | 97.99 | C42 H79 O10 P | 7.212 | LMGP04010197 |
| PG(17:1(9Z)/17:0) | Lipid Pos | 75.52 | C40 H77 O10 P | 7.280 | LMGP04010256 |
| PG(18:1(9Z)/20:4(5Z,8Z,11Z,14Z)) | Lipid Neg | 97.48 | C44 H77 O10 P | 7.018 | HMDB10640 |
| PG(18:3(6Z,9Z,12Z)/18:0) | Lipid Pos | 73.40 | C42 H77 O10 P | 6.163 | HMDB10662 |
| PG(O-18:0/0:0) | Lipid Pos | 72.76 | C24 H51 O8 P | 1.128 | LMGP04060002 |
| PG(O-18:0/17:1(9Z)) | Lipid Pos | 74.45 | C41 H81 O9 P | 3.165 | LMGP04020025 |
| PG(O-20:0/17:0) | Lipid Pos | 71.34 | C43 H87 O9 P | 3.718 | LMGP04020048 |
| Pheniramine | Lipid Neg | 85.30 | C16 H20 N2 | 2.870 | HMDB15557 |
| Phenylalanine* | Aqueous | 86.23 | C9 H11 N O2 | 2.262 | KEGG: C00079 |
| Phenylgalactoside | Lipid Pos | 70.40 | C12 H16 O6 | 1.140 | KEGG: C02578 |
| Phthalic acid* | Lipid | 76.67 | C8 H6 O4 | 3.177 | KEGG: C01606 |
| phyllohydroquinone | Lipid Pos | 86.06 | C31 H48 O2 | 2.206 | KEGG: C03313 |
| Phytanic acid | Lipid Pos | 84.43 | C20 H40 O2 | 6.321 | KEGG: C01607 |
| Phytanol | Lipid Pos | 98.01 | C20 H42 O | 2.008 | LMPR0104010006 |
| PI(14:0/20:2(11Z,14Z)) | Lipid Neg | 99.39 | C43 H79 O13 P | 5.325 | LMGP06010071 |
| PI(16:0/20:4(8Z,11Z,14Z,17Z)) | Lipid Pos | 88.59 | C45 H79 O13 P | 5.094 | KEGG: C00626 |
| PI(16:1(9Z)/22:4(7Z,10Z,13Z,16Z)) | Lipid Neg | 99.58 | C47 H81 O13 P | 5.278 | LMGP06010193 |
| PI(16:2(9Z,12Z)/22:3(10Z,13Z,16Z)) | Lipid Neg | 99.28 | C47 H81 O13 P | 5.278 | KEGG: C00626 |
| PI(18:1(9Z)/18:0) | Lipid Pos | 77.08 | C45 H85 O13 P | 6.073 | LMGP06010932 |
| PI(19:1(9Z)/13:0) | Lipid Pos | 73.74 | C41 H77 O13 P | 6.173 | LMGP06010449 |
| PI(20:0/20:3(8Z,11Z,14Z)) | Lipid Neg | 99.66 | C49 H89 O13 P | 4.536 | LMGP06010490 |
| PI(20:0/20:3(8Z,11Z,14Z)) | Lipid Pos | 94.14 | C49 H89 O13 P | 4.044 | KEGG: C01194 |
| PI(20:3(8Z,11Z,14Z)/18:3(6Z,9Z,12Z)) | Lipid Neg | 97.52 | C47 H79 O13 P | 5.040 | LMGP06010571 |
| PI(20:4(5Z,8Z,11Z,14Z)/18:2(9Z,12Z)) | Lipid Pos | 73.57 | C47 H79 O13 P | 4.876 | LMGP06010600 |
| PI(21:0/20:2(11Z,14Z)) | Lipid Pos | 71.62 | C50 H93 O13 P | 1.029 | KEGG: C01194 |
| PI(O-16:0/14:0) | Lipid Pos | 97.04 | C39 H77 O12 P | 1.000 | LMGP06020004 |
| PI(O-18:0/12:0) | Lipid Pos | 96.33 | C39 H77 O12 P | 1.005 | LMGP06020019 |
| PI(P-18:0/16:1(9Z)) | Lipid Pos | 96.67 | C43 H81 O12 P | 3.172 | LMGP06030036 |
| Pipercide | Lipid Neg | 99.33 | C22 H29 N O3 | 2.239 | HMDB33449 |
| Piperidine | Aqueous | 85.99 | C5 H11 N | 1.472 | KEGG: C01746 |
| Polyethylene glycol | Lipid Neg | 82.66 | C9 H17 N O3 | 4.016 | KEGG: C01092 |
| Pregna-4,16-diene-3,20-dione | Lipid Neg | 98.25 | C21 H28 O2 | 1.926 | KEGG: C03207 |
| Proline betaine | Lipid Pos | 90.36 | C7 H13 N O2 | 2.468 | KEGG: C10172 |
| Prolyl-2-naphthylamide | Lipid Pos | 78.20 | C15 H16 N2 O | 10.483 | KEGG: C03305 |
| Propafenone | Lipid Neg | 99.79 | C21 H27 N O3 | 2.108 | KEGG: C07381 |
| Propionylcarnitine | Aqueous | 94.14 | C10 H19 N O4 | 2.147 | KEGG: C03017 |
| Propylene glycol | Lipid Pos | 80.89 | C3 H8 O2 | 10.459 | KEGG: C00583 |
| PS(12:0/21:0) | Lipid Neg | 98.34 | C39 H76 N O10 P | 4.993 | LMGP03010064 |
| PS(13:0/22:2(13Z,16Z)) | Lipid Neg | 96.56 | C41 H76 N O10 P | 5.608 | LMGP03010090 |
| PS(16:1(9Z)/19:0) | Lipid Neg | 99.09 | C41 H78 N O10 P | 5.216 | LMGP03010214 |
| PS(17:0/19:0) | Lipid Neg | 92.25 | C42 H82 N O10 P | 6.152 | LMGP03010237 |
| PS(18:0/19:1(9Z)) | Lipid Neg | 99.93 | C43 H82 N O10 P | 5.863 | LMGP03010318 |
| PS(19:0/16:0) | Lipid Neg | 100.00 | C41 H80 N O10 P | 5.722 | LMGP03010953 |
| PS(19:0/18:3(9Z,12Z,15Z)) | Lipid Neg | 95.81 | C43 H78 N O10 P | 5.751 | LMGP03010465 |
| PS(19:0/20:2(11Z,14Z)) | Lipid Neg | 99.77 | C45 H84 N O10 P | 6.058 | LMGP03010470 |
| PS(19:0/20:2(11Z,14Z)) | Lipid Neg | 95.89 | C45 H84 N O10 P | 7.156 | LMGP03010470 |
| PS(19:0/20:3(8Z,11Z,14Z)) | Lipid Neg | 99.98 | C45 H82 N O10 P | 5.738 | LMGP03010471 |
| PS(19:0/20:3(8Z,11Z,14Z)) | Lipid Neg | 96.82 | C45 H82 N O10 P | 5.420 | LMGP03010471 |
| PS(19:0/20:3(8Z,11Z,14Z)) | Lipid Neg | 89.96 | C45 H82 N O10 P | 5.539 | LMGP03010471 |
| PS(20:1(11Z)/18:3(6Z,9Z,12Z)) | Lipid Pos | 80.09 | C44 H78 N O10 P | 0.998 | LMGP03010542 |
| PS(20:1(11Z)/18:3(6Z,9Z,12Z)) | Lipid Pos | 76.47 | C44 H78 N O10 P | 10.464 | LMGP03010542 |
| PS(20:2(11Z,14Z)/17:0) | Lipid Neg | 98.46 | C43 H80 N O10 P | 5.540 | LMGP03010566 |
| PS(20:2(11Z,14Z)/19:1(9Z)) | Lipid Neg | 98.61 | C45 H82 N O10 P | 5.420 | LMGP03010576 |
| PS(20:3(8Z,11Z,14Z)/21:0) | Lipid Neg | 99.48 | C47 H86 N O10 P | 6.438 | LMGP03010614 |
| PS(20:4(5Z,8Z,11Z,14Z)/21:0) | Lipid Neg | 99.07 | C47 H84 N O10 P | 5.804 | LMGP03010643 |
| PS(22:2(13Z,16Z)/17:2(9Z,12Z)) | Lipid Neg | 93.56 | C45 H80 N O10 P | 5.298 | LMGP03010766 |
| PS(22:2(13Z,16Z)/17:2(9Z,12Z)) | Lipid Neg | 94.73 | C45 H80 N O10 P | 5.885 | LMGP03010766 |
| PS(22:4(7Z,10Z,13Z,16Z)/19:0) | Lipid Neg | 87.79 | C47 H84 N O10 P | 6.015 | LMGP03010804 |
| PS(22:4(7Z,10Z,13Z,16Z)/19:0) | Lipid Neg | 96.52 | C47 H84 N O10 P | 6.654 | LMGP03010804 |
| PS(22:4(7Z,10Z,13Z,16Z)/19:1(9Z)) | Lipid Neg | 99.88 | C47 H82 N O10 P | 5.574 | LMGP03010805 |
| PS(O-16:0/18:3(6Z,9Z,12Z)) | Lipid Neg | 94.86 | C40 H74 N O9 P | 5.595 | LMGP03020078 |
| PS(O-18:0/0:0) | Lipid Pos | 81.14 | C24 H50 N O8 P | 1.357 | LMGP03060002 |
| PS(O-18:0/13:0) | Lipid Pos | 91.69 | C37 H74 N O9 P | 5.206 | LMGP03020019 |
| PS(O-18:0/16:1(9Z)) | Lipid Pos | 94.88 | C40 H78 N O9 P | 4.918 | LMGP03020072 |
| PS(O-20:0/0:0) | Lipid Pos | 92.78 | C26 H54 N O8 P | 1.676 | LMGP03060001 |
| PS(O-20:0/18:2(9Z,12Z)) | Lipid Pos | 79.60 | C44 H84 N O9 P | 5.310 | LMGP03020053 |
| PS(P-18:0/15:1(9Z)) | Lipid Pos | 80.24 | C39 H74 N O9 P | 4.827 | LMGP03030033 |
| PS(P-18:0/17:2(9Z,12Z)) | Lipid Pos | 96.95 | C41 H76 N O9 P | 5.263 | LMGP03030038 |
| PS(P-18:0/22:6(4Z,7Z,10Z,13Z,16Z,19Z)) | Lipid Pos | 91.26 | C46 H78 N O9 P | 5.009 | LMGP03030089 |
| PS(P-20:0/17:2(9Z,12Z)) | Lipid Pos | 83.40 | C43 H80 N O9 P | 5.870 | LMGP03030066 |
| PS(P-20:0/20:1(11Z)) | Lipid Pos | 75.48 | C46 H88 N O9 P | 6.037 | LMGP03030076 |
| Pyranocyanin A | Lipid Pos | 89.45 | C30 H33 O15 | 1.042 | HMDB35420 |
| Pyrroline hydroxycarboxylic acid | Aqueous | 87.53 | C5 H7 N O3 | 1.713 | KEGG: C04281 |
| Quinoxaline | Lipid Pos | 92.37 | C8 H6 N2 | 0.881 | KEGG: C18575 |
| Rec-β-Tocopherol | Lipid Pos | 80.34 | C28 H48 O2 | 2.654 | KEGG: C14152 |
| Retapamulin | Lipid Pos | 79.22 | C30 H47 N O4 S | 4.427 | HMDB15386 |
| Rishitin | Lipid Neg | 86.12 | C14 H22 O2 | 1.349 | KEGG: C09715 |
| Salannin | Lipid Pos | 98.18 | C34 H44 O9 | 1.353 | KEGG: C08780 |
| Sapelin A | Lipid Pos | 90.09 | C30 H50 O4 | 3.284 | KEGG: C08634 |
| Sintaxanthin | Lipid Pos | 84.47 | C31 H42 O | 2.262 | HMDB35640 |
| SM(d16:1/24:0) | Lipid Neg | 85.20 | C45 H91 N2 O6 P | 3.728 | LMSP03010073 |
| SM(d18:0/22:0) | Lipid Pos | 78.90 | C45 H93 N2 O6 P | 6.056 | LMSP03010022 |
| SM(d18:0/24:1(15Z)) | Lipid Pos | 80.64 | C47 H95 N2 O6 P | 7.945 | KEGG: C00550 |
| SM(d18:2/21:0) | Lipid Neg | 83.70 | C44 H87 N2 O6 P | 2.867 | LMSP03010064 |
| SM(d18:2/22:1) | Lipid Pos | 88.08 | C45 H87 N2 O6 P | 2.466 | LMSP03010070 |
| Sodium Tetradecyl Sulfate | Lipid Neg | 76.24 | C14 H30 O4 S | 2.027 | HMDB14607 |
| Sodium Tetradecyl Sulfate | Lipid Pos | 71.40 | C14 H30 O4 S | 1.169 | HMDB14607 |
| Sorgolactone | Lipid Pos | 81.98 | C18 H20 O5 | 0.982 | KEGG: C09186 |
| Sphinganine | Lipid Pos | 84.08 | C18 H39 N O2 | 2.860 | KEGG: C00836 |
| Sphinganine | Lipid Pos | 79.02 | C18 H39 N O2 | 3.863 | KEGG: C00836 |
| S-Ribosyl-L-homocysteine | Lipid Pos | 85.02 | C9 H17 N O6 S | 1.541 | KEGG: C03539 |
| Stearoyl-EA | Lipid Pos | 93.67 | C20 H41 N O2 | 2.966 | LMFA08040051 |
| Stearyl alcohol | Lipid Pos | 74.75 | C18 H38 O | 3.903 | LMFA05000085 |
| Stearyl citrate | Lipid Pos | 84.08 | C24 H44 O7 | 2.913 | HMDB32521 |
| Stigmast-5,22E-dien-3β-yl (13Z,16Z,19Z-docosatrienoate) | Lipid Neg | 82.55 | C51 H84 O2 | 4.019 | LMST01020088 |
| Styrene | Lipid Pos | 85.05 | C8 H8 | 1.580 | KEGG: C07083 |
| Succinylcholine | Lipid Pos | 86.79 | C14 H30 N2 O4 | 1.029 | KEGG: C07546 |
| Talinolol | Lipid Pos | 85.62 | C20 H33 N3 O3 | 2.030 | HMDB42020 |
| Tanacetol B | Lipid Neg | 84.95 | C17 H28 O4 | 2.542 | HMDB35075 |
| Tangeraxanthin | Lipid Pos | 90.43 | C34 H44 O2 | 1.518 | HMDB39015 |
| Tephrosone | Lipid Pos | 83.85 | C21 H20 O5 | 1.291 | LMPK12120208 |
| Terephthalic acid | Lipid Pos | 87.78 | C8 H6 O4 | 1.352 | KEGG: C06337 |
| Testosterone | Lipid Pos | 74.63 | C19 H28 O2 | 2.069 | KEGG: C00535 |
| Tetradecyl isobutyrate | Lipid Pos | 89.94 | C18 H36 O2 | 2.466 | LMFA07010679 |
| Tetratriacontan-1-ol | Lipid Pos | 76.73 | C34 H70 O | 5.582 | LMFA05000459 |
| TG(12:0/12:0/12:0) | Lipid Pos | 91.09 | C39 H74 O6 | 6.811 | KEGG: C00422 |
| TG(12:0/15:0/18:3(6Z,9Z,12Z)) | Lipid Pos | 93.20 | C48 H86 O6 | 8.244 | KEGG: C00422 |
| TG(14:0/o-18:0/18:0) | Lipid Pos | 94.28 | C53 H104 O5 | 3.175 | KEGG: C00422 |
| TG(14:1(9Z)/o-18:0/18:1(11Z)) | Lipid Pos | 90.17 | C53 H100 O5 | 8.286 | HMDB48393 |
| TG(16:0/20:1(11Z)/16:1(9Z)) | Lipid Pos | 76.84 | C55 H102 O6 | 9.574 | HMDB44143 |
| TG(18:1(11Z)/16:1(9Z)/20:3(5Z,8Z,11Z)) | Lipid Neg | 95.34 | C57 H100 O6 | 3.726 | HMDB49243 |
| TG(18:1/15:0/22:6) | Lipid Pos | 71.91 | C58 H98 O6 | 1.029 | KEGG: C00422 |
| TG(18:3/20:1/22:6) | Lipid Neg | 88.42 | C63 H102 O6 | 3.726 | HMDB53011 |
| TG(20:4/18:3/18:4) | Lipid Pos | 72.83 | C59 H92 O6 | 6.187 | HMDB54193 |
| TG(24:1/22:2/22:6) | Lipid Pos | 79.83 | C71 H120 O6 | 2.932 | HMDB52251 |
| TG(24:1/22:5/22:5) | Lipid Pos | 70.82 | C71 H116 O6 | 2.467 | HMDB52277 |
| TG(24:1/24:0/22:6) | Lipid Neg | 85.31 | C73 H128 O6 | 3.613 | HMDB52027 |
| Thromboxane | Lipid Pos | 84.11 | C20 H40 O | 1.354 | KEGG: C02198 |
| Trans-1,2-dimethylcyclohexane | Lipid Neg | 98.84 | C8 H16 | 3.615 | LMFA11000637 |
| Trans-2-Dodecenoylcarnitine | Lipid Pos | 91.14 | C19 H35 N O4 | 3.176 | HMDB13326 |
| Trans-Resveratrol 3,5-disulfate | Lipid Neg | 74.61 | C14 H12 O9 S2 | 1.674 | HMDB41781 |
| Triamcinolone | Lipid Neg | 97.05 | C21 H27 F O6 | 2.333 | HMDB14758 |
| Triamiphos | Lipid Pos | 81.11 | C12 H19 N6 O P | 0.770 | KEGG: C18927 |
| Tributyl phosphate | Lipid Pos | 92.67 | C12 H27 O4 P | 1.312 | KEGG: C14439 |
| Trichostatin | Lipid Neg | 78.31 | C17 H22 N2 O3 | 1.363 | LMPK01000055 |
| Tridihexethyl | Lipid Pos | 75.86 | C21 H36 N O | 1.791 | KEGG: C07861 |
| Tridodecylamine | Lipid Pos | 81.36 | C36 H75 N | 6.303 | HMDB37822 |
| Trifluoroacetic acid | Lipid Neg | 87.99 | C2 H F3 O2 | 0.344 | HMDB14118 |
| Triphenyl phosphate | Lipid Pos | 70.23 | C18 H15 O4 P | 1.178 | KEGG: C14235 |
| Triptolide | Lipid Pos | 97.29 | C20 H24 O6 | 1.220 | KEGG: C09204 |
| Tris(2-butoxyethyl) phosphate | Lipid Pos | 80.36 | C18 H39 O7 P | 1.349 |  |
| Trp Leu Lys | Lipid Pos | 81.48 | C23 H35 N5 O4 | 2.450 |  |
| Tryptophan* | Aqueous | 87.20 | C11 H12 N2 O2 | 4.602 | KEGG: C00078 |
| Tuberculostearic acid | Lipid Neg | 94.01 | C19 H38 O2 | 4.433 | KEGG: C16794 |
| TyrMe-Phe-OH | Lipid Pos | 70.17 | C25 H24 N2 O7 | 1.696 |  |
| Tyromycic acid | Lipid Pos | 82.95 | C30 H44 O3 | 3.721 | HMDB35888 |
| Tyrosine* | Aqueous | 99.43 | C9 H11 N O3 | 1.846 | KEGG: C00082 |
| Umbelliferone | Lipid Pos | 78.60 | C9 H6 O3 | 10.564 | KEGG: C09315 |
| Uric acid | Aqueous | 85.54 | C5 H4 N4 O3 | 2.302 | KEGG: C00366 |
| Uvaricin | Lipid Pos | 87.69 | C39 H68 O7 | 3.283 | KEGG: C08572 |
| Val Val Trp | Lipid Pos | 75.79 | C21 H30 N4 O4 | 1.491 |  |
| Valeroyl Salicylate | Lipid Pos | 94.73 | C12 H14 O4 | 1.352 | CAS: 64206-54-8 |
| Valtratum | Lipid Neg | 99.70 | C22 H30 O8 | 0.841 | KEGG: C09801 |
| Varanic acid | Lipid Pos | 87.53 | C26 H44 O5 | 3.181 | HMDB02195 |
| Varanic acid | Lipid Pos | 92.59 | C26 H44 O5 | 3.722 | HMDB02195 |
| Vitamin A2 aldehyde | Lipid Pos | 95.22 | C20 H26 O | 2.389 | KEGG: C05918 |
| Vitamin K1 | Lipid Pos | 70.14 | C31 H46 O2 | 2.651 | KEGG: C02059 |
| Vorinostat | Lipid Neg | 84.60 | C14 H20 N2 O3 | 2.002 | HMDB15568 |

# Supplemental Table S5: List of selected overlapping annotated metabolites in human and mouse plasma

Metabolites were annotated using exact mass, isotope ratios, and isotopic distribution. The listed metabolites have an error < 10ppm with database scores > 70 out of a possible 100.

| **Compound** | **Fraction** | **Score** | **Formula** | **RT (min)** | **ID** |
| --- | --- | --- | --- | --- | --- |
| α-Hydroxy myristic acid | Lipid Pos | 94.75 | C14 H28 O3 | 1.142 | LMFA01050001 |
| α-Tocopheronic acid | Lipid Pos | 87.90 | C16 H24 O5 | 3.171 | LMPR02020062 |
| β-Tocopherol | Lipid Pos | 80.34 | C28 H48 O2 | 2.654 | KEGG: C14152 |
| (-)-Aspidospermine | Lipid Neg | 83.57 | C22 H30 N2 O2 | 3.726 | HMDB30361 |
| (+)-12-Methyl myristic acid | Lipid Pos | 86.13 | C15 H30 O2 | 2.462 | KEGG: C16665 |
| (+)-Aspidospermidine | Lipid Neg | 83.63 | C19 H26 N2 | 3.614 | HMDB30360 |
| (+)-Tephrosone | Lipid Pos | 83.85 | C21 H20 O5 | 1.291 | LMPK12120208 |
| (+/-)-2-methyl-5,8,11,14-all-cis-tricosatetraenoyl-2'-fluoroethylamine | Lipid Pos | 83.96 | C26 H44 F N O | 3.266 | LMFA08020071 |
| (10Z)-19-fluoro-1α,25-dihydroxyvitamin D3 | Lipid Neg | 93.78 | C27 H43 F O3 | 4.679 | LMST03020212 |
| (17Z)-1α,25-dihydroxy-26,27-dimethyl-17,20,22,22,23,23-hexadehydrovitamin D3 | Lipid Neg | 70.13 | C29 H42 O3 | 2.886 | LMST03020391 |
| (20R)-Ginsenoside Rh2 | Lipid Neg | 98.55 | C36 H62 O8 | 4.299 | HMDB39544 |
| (24R)-24-fluoro-1 α,25-dihydroxyvitamin D2 | Lipid Pos | 84.64 | C28 H43 F O3 | 1.709 | LMST03010011 |
| (24R)-25-fluoro-1α,24-dihydroxy-24-methylvitamin D3 | Lipid Neg | 72.18 | C28 H45 F O3 | 4.984 | LMST03020328 |
| (25S)-3alpha,7alpha,12alpha,24R-tetrahydroxy-5beta-cholestan-26-oic acid | Lipid Pos | 83.76 | C27 H46 O6 | 3.177 | LMST04030194 |
| (2S)-2-hydroxyphytanic acid | Lipid Pos | 83.50 | C20 H40 O3 | 3.175 | KEGG: C02982 |
| (3a,5b)-24-oxo-24-[(2-sulfoethyl)amino]cholan-3-yl-b-D-Glucopyranosiduronic acid | Lipid Neg | 89.62 | C32 H53 N O11 S | 2.425 | HMDB02429 |
| (3beta,17alpha,23S)-17,23-Epoxy-3,29-dihydroxy-27-norlanosta-7,9(11)-diene-15,24-dione | Lipid Pos | 83.21 | C29 H42 O5 | 3.172 | HMDB35970 |
| (3R,7R)-1,3,7-Octanetriol | Lipid Pos | 72.59 | C8 H18 O3 | 1.082 | HMDB33625 |
| (3R,7R)-1,3,7-Octanetriol | Lipid Pos | 81.68 | C8 H18 O3 | 0.727 | HMDB33625 |
| (3'-sulfo)Galβ-Ceramide (d18:1/20:0) | Lipid Neg | 70.91 | C44 H85 N O8 | 6.533 | LMSP06020010 |
| (7R)-7-(5-Carboxy-5-oxopentanoyl)aminocephalosporinate | Lipid Neg | 96.28 | C16 H18 N2 O9 S | 0.869 | HMDB60316 |
| (all-E)-1,7,9-Heptadecatriene-11,13,15-triyne | Lipid Neg | 91.76 | C17 H18 | 2.934 | HMDB31260 |
| (E)-7-Pentadecene | Lipid Neg | 97.92 | C15 H30 | 3.225 | HMDB31083 |
| (R)-10-hydroxystearic acid | Lipid Pos | 72.25 | C18 H36 O3 | 2.927 | LMFA02000237 |
| (R*,S*)-4-[1-Ethyl-2-(4-fluorophenyl)butyl]phenol | Lipid Pos | 76.16 | C18 H21 F O | 0.611 | KEGG: C15463 |
| (S)-10,16-Dihydroxyhexadecanoic acid | Lipid Pos | 92.68 | C16 H32 O4 | 1.155 | HMDB37798 |
| (S)-Neolyratyl acetate | Lipid Neg | 86.55 | C12 H18 O2 | 1.323 | HMDB41497 |
| (Z)-13-Docosenamide* | Lipid Pos | 99.18 | C22 H43 N O | 3.708 | PubChem: 5365371 |
| (Z,Z)-2-Methyl-5-(8,11,14-pentadecatrienyl)-1,3-benzenediol | Lipid Neg | 98.65 | C22 H32 O2 | 2.686 | HMDB38908 |
| [2-(4-Methylphenyl)-1,3-dioxolan-4-yl]methanol | Lipid Neg | 86.52 | C11 H14 O3 | 1.270 | HMDB59942 |
| 1,2-Cyclohexanediol | Lipid Pos | 73.86 | C6 H12 O2 | 10.453 | CAS: 931-17-9 |
| 1,2-tetracosanediol | Lipid Pos | 94.41 | C24 H50 O2 | 6.963 | LMFA05000083 |
| 1,3-Propanediol | Lipid Pos | 81.19 | C3 H8 O2 | 10.459 | CAS: 504-63-2 |
| 1,4-Dihydroxy-2-naphthoyl-CoA | Aqueous | 94.21 | C32 H42 N7 O19 P3 S | 6.665 | KEGG: C15547 |
| 1-[1,4-Dihydro-4-nonyl-5-(1-oxodecyl)-3-pyridinyl]-1-dodecanone | Lipid Neg | 83.72 | C36 H65 N O2 | 6.800 | HMDB35518 |
| 10,12-Tetradecadienal | Lipid Pos | 91.81 | C14 H24 O | 1.143 | LMFA06000187 |
| 11R-HETE | Lipid Pos | 83.32 | C20 H32 O3 | 2.447 | LMFA03060028 |
| 11R-HpOME | Aqueous | 80.42 | C18 H34 O4 | 0.395 | LMFA02000067 |
| 12'-Apo-b-carotene-3,12'-diol | Lipid Neg | 83.51 | C25 H36 O2 | 2.945 | HMDB36054 |
| 13,14-dihydroxy-docosanoic acid | Lipid Pos | 82.42 | C22 H44 O4 | 3.286 | LMFA01050211 |
| 13-Hydroxy-9E,11E-octadecadienoic acid | Lipid Neg | 84.16 | C18 H32 O3 | 1.730 | LMFA02000155 |
| 13S-hydroxy11E-octadecenoic acid | Lipid Neg | 85.91 | C18 H34 O3 | 1.794 | LMFA02000094 |
| 13Z,16Z-docosadienoic acid | Lipid Pos | 93.85 | C22 H40 O2 | 3.150 | KEGG: C16533 |
| 15-epi-15-iso-LGE2 | Lipid Neg | 78.99 | C20 H32 O5 | 3.234 | LMFA03100005 |
| 15-methyl-hexadecasphingosine | Lipid Pos | 77.16 | C17 H35 N O2 | 3.716 | LMSP01080005 |
| 15-oxo-octadecanoic acid | Lipid Pos | 95.38 | C18 H34 O3 | 2.443 | LMFA02000259 |
| 15β-Hydroxydesogestrel | Lipid Neg | 79.06 | C22 H30 O2 | 2.793 | HMDB60707 |
| 16,17-Didehydroprogesterone | Lipid Neg | 98.25 | C21 H28 O2 | 1.926 | LMST02030163 |
| 16:0 Campesteryl ester | Lipid Neg | 90.33 | C44 H78 O2 | 7.553 | LMST01020043 |
| 16-methyl-9Z,12Z-heptadecadienoic acid | Lipid Neg | 99.08 | C18 H32 O2 | 2.864 | LMFA01020208 |
| 17-Methylandrosta-2,4-dieno[2,3-d]isoxazol-17beta-ol | Lipid Pos | 80.41 | C21 H29 N O2 | 1.314 | KEGG: C15177 |
| 17-phenyl trinor PGF2α isopropyl ester | Lipid Pos | 93.62 | C26 H38 O5 | 1.990 | LMFA03010124 |
| 18-Nor-4(19),8,11,13-abietatetraene | Lipid Neg | 85.32 | C19 H26 | 2.236 | HMDB41371 |
| 18-Oxocortisol | Lipid Pos | 92.13 | C21 H28 O6 | 0.759 | LMST02030194 |
| 19-hydroxy-nonadecanoic acid | Lipid Pos | 99.44 | C19 H38 O3 | 3.283 | METLIN: 35450 |
| 1alpha,25-dihydroxy-3-deoxy-3-thiavitamin D3 | Lipid Pos | 79.95 | C26 H42 O2 S | 1.032 | LMST03020041 |
| 1-Deoxy-D-glucitol | Aqueous | 76.67 | C6 H14 O5 | 0.632 | HMDB41500 |
| 1-Hexadecyl-2-O-methyl-glycerol | Lipid Pos | 87.80 | C20 H42 O3 | 3.180 | CAS: 111188-59-1 |
| 1-Methoxy-1-pentyloxyethane | Lipid Pos | 87.20 | C8 H18 O2 | 0.791 | HMDB38679 |
| 1-Methyluric acid | Lipid Pos | 83.22 | C6 H6 N4 O3 | 0.451 | KEGG: C16359 |
| 1-Nonadecene | Aqueous | 78.64 | C19 H38 | 1.414 | LMFA11000322 |
| 1-O-alpha-D-glucopyranosyl-1,2-eicosandiol | Lipid Neg | 99.81 | C26 H52 O7 | 5.484 | LMFA13010005 |
| 1-Octacosene | Aqueous | 77.09 | C28 H56 | 0.765 | LMFA11000324 |
| 1-Octacosene | Lipid Neg | 99.03 | C28 H56 | 2.874 | LMFA11000324 |
| 1α-hydroxy-22-(3-methylphenyl)-23,24,25,26,27-pentanorvitamin D3 | Lipid Neg | 76.71 | C29 H40 O2 | 4.397 | LMST03020390 |
| 2-(8-[3]-ladderane-octanyl)-sn-glycero-3-phosphoethanolamine | Lipid Neg | 96.27 | C25 H46 N O6 P | 1.821 | LMGP02060001 |
| 2-(9R-(tricosanoyloxy)-3-methyl-2Z-decenoyloxy)-ethanesulfonic acid | Lipid Pos | 83.53 | C36 H68 O7 S | 1.563 | LMFA07020004 |
| 2(R)-HPOT | Lipid Pos | 77.00 | C18 H30 O4 | 1.154 | KEGG: C16341 |
| 2,6-dimethyl-pentadecanoic acid | Lipid Pos | 96.44 | C17 H34 O2 | 3.176 | LMFA01020040 |
| 2,8-dimethyl-tetradecanoic acid | Lipid Neg | 98.65 | C16 H32 O2 | 3.229 | LMFA01020039 |
| 2-[4,6-Bis(2,4-dimethylphenyl)-1,3,5-triazin-2-yl]-5-(octyloxy)phenol | Lipid Neg | 72.08 | C33 H39 N3 O2 | 1.750 | HMDB37802 |
| 2-[4,6-Bis(2,4-dimethylphenyl)-1,3,5-triazin-2-yl]-5-(octyloxy)phenol | Lipid Neg | 73.33 | C33 H39 N3 O2 | 1.806 | HMDB37802 |
| 20, 22-Dihydrodigoxigenin | Lipid Neg | 79.13 | C23 H36 O5 | 3.727 | HMDB60730 |
| 20:0 Campesteryl ester | Lipid Neg | 89.99 | C48 H86 O2 | 2.873 | LMST01020045 |
| 21-Deoxycortisol | Lipid Pos | 98.43 | C21 H30 O4 | 1.525 | KEGG: C05497 |
| 22,23-Methylene-24-methyl-cholest-5-en-3beta-ol | Lipid Neg | 76.86 | C29 H48 O | 5.023 | LMST01030138 |
| 22-epi-Hippurin-1 | Lipid Neg | 93.49 | C30 H48 O7 | 1.943 | LMST01090004 |
| 24,24-Difluoro-25-hydroxy-26,27-dimethylvitamin D3 | Aqueous | 76.37 | C29 H46 F2 O2 | 3.179 | LMST03020676 |
| 24-ethyl-5alpha-cholest-25-en-3alpha,12alpha,16alpha-triol | Lipid Pos | 95.95 | C29 H50 O3 | 4.369 | LMST01040188 |
| 25-Acetoxy-ergosta-3beta,5alpha,6beta-triol | Lipid Neg | 70.85 | C30 H52 O5 | 5.488 | LMST01031059 |
| 2-Aminobenzimidazole | Lipid Pos | 75.36 | C7 H7 N3 | 1.792 | CAS: 934-32-7 |
| 2-Butoxyethanol | Lipid Pos | 86.54 | C6 H14 O2 | 10.537 | CAS: 111-76-2 |
| 2-Deoxystreptidine | Lipid Pos | 73.18 | C8 H18 N6 O3 | 0.660 | KEGG: C02628 |
| 2-ethyl-1,5-dimethyl-3,3-diphenylpyrrolinium (EDDP) | Lipid Neg | 84.09 | C20 H24 N | 2.870 | HMDB60931 |
| 2-Furoylglycine | Aqueous | 85.82 | C7 H7 N O4 | 10.211 | HMDB00439 |
| 2-Furoylglycine | Aqueous | 89.44 | C7 H7 N O4 | 6.053 | HMDB00439 |
| 2-Heptenal | Lipid Pos | 71.44 | C7 H12 O | 1.145 | LMFA06000019 |
| 2-Hexadecanoyl-sn-glycero-3-phosphocholine | Lipid Neg | 79.95 | C24 H50 N O7 P | 1.884 | LMGP01050074 |
| 2-Hydroxyenterodiol | Lipid Pos | 83.51 | C18 H22 O5 | 1.352 | HMDB41649 |
| 2-Hydroxy-heneicosanoic acid | Lipid Pos | 74.60 | C21 H42 O3 | 2.880 | METLIN: 35575 |
| 2-Oxo-4-methylthio-butanoic acid | Lipid Pos | 92.89 | C5 H8 O3 S | 10.484 | KEGG: C01180 |
| 2-Oxo-8-methylthiooctanoic acid | Lipid Pos | 77.63 | C9 H16 O3 S | 10.485 | KEGG: C17224 |
| 2S-hydroxylauric acid | Lipid Pos | 95.00 | C12 H24 O3 | 1.115 | LMFA01050362 |
| 2β,3α,12α-Trihydroxy-5β-cholan-24-oic acid | Lipid Pos | 95.02 | C24 H40 O5 | 1.992 | LMST04010076 |
| 3-(2,4-Cyclopentadien-1-ylidene)-5alpha-androstan-17beta-ol | Lipid Pos | 77.87 | C24 H34 O | 3.568 | KEGG: C14915 |
| 3,4-Dihydroxyrottlerin | Aqueous | 85.07 | C30 H28 O10 | 0.865 | LMPK12120269 |
| 3,5-Dimethylpentadecanoic acid | Lipid Neg | 99.68 | C17 H34 O2 | 3.614 | LMFA01020390 |
| 3,7-Bisaboladiene-2,8-dione | Lipid Neg | 86.35 | C15 H22 O2 | 1.625 | HMDB38198 |
| 3,7R,11R,15-tetramethylhexadecan-1-ol | Lipid Neg | 70.46 | C20 H42 O | 3.364 | LMPR0104010006 |
| 31-hydroxy-32,35-anhydrobacteriohopanetetrol | Lipid Neg | 96.20 | C35 H60 O4 | 2.867 | LMPR04000027 |
| 35-aminobacteriohopane-30,31,32,33,34-pentol | Lipid Pos | 86.76 | C35 H63 N O5 | 1.584 | LMPR04000009 |
| 3alpha,12alpha-Dihydroxy-5beta-chol-8(14)-en-24-oic Acid | Aqueous | 79.19 | C24 H38 O4 | 0.570 | LMST04010224 |
| 3b-Allotetrahydrocortisol | Lipid Neg | 78.55 | C21 H34 O5 | 3.613 | HMDB00314 |
| 3-Decaprenyl-4,5-dihydroxybenzoate | Aqueous | 89.21 | C57 H86 O4 | 3.701 | HMDB60249 |
| 3-Dehydroxycarnitine | Aqueous | 74.39 | C7 H15 N O2 | 8.716 | HMDB06831 |
| 3-Ethyl-7,11-dimethyltrideca-1,3Z,6E,10-tetraene | Lipid Neg | 98.28 | C17 H30 | 2.864 | LMFA11000123 |
| 3-Hexadecanoyloleanolic acid | Lipid Pos | 84.77 | C46 H78 O4 | 7.517 | HMDB36967 |
| 3-Hexanone | Lipid Neg | 99.38 | C6 H12 O | 0.855 | HMDB00753 |
| 3-Hydroxy-2-methyl-3-phytyl-2,3-dihydro-1,4-naphthoquinone | Lipid Pos | 81.16 | C31 H48 O3 | 1.676 | LMPR02030029 |
| 3-Hydroxyadipic acid | Aqueous | 88.73 | C6 H10 O5 | 0.866 | HMDB00345 |
| 3-Methyl sulfolene | Aqueous | 99.68 | C5 H8 O2 S | 2.349 | HMDB59667 |
| 3-Methyldioxyindole | Lipid Pos | 72.23 | C9 H9 N O2 | 1.223 | HMDB04186 |
| 3-Methylhistamine | Lipid Neg | 95.35 | C6 H11 N3 | 2.878 | HMDB01861 |
| 3-O-(beta-D-glucopyranosyl)-campest-5-en-3beta-ol | Lipid Neg | 71.55 | C34 H58 O6 | 4.033 | LMST01031126 |
| 3-Octaprenyl-4-hydroxybenzoate | Lipid Pos | 95.00 | C47 H70 O3 | 7.150 | KEGG: C05809 |
| 3-Oxocholic acid | Lipid Pos | 92.98 | C24 H38 O5 | 1.664 | HMDB00502 |
| 3-Oxododecanoic acid | Lipid Pos | 83.45 | C12 H22 O3 | 0.969 | KEGG: C02367 |
| 3-Oxosteroid | Lipid Pos | 83.51 | C19 H30 O | 1.992 | KEGG: C01876 |
| 3-Succinoylpyridine | Aqueous | 86.45 | C9 H9 N O3 | 0.695 | HMDB00992 |
| 3Z-Octadecenoic acid | Lipid Neg | 99.92 | C18 H34 O2 | 3.360 | LMFA01030294 |
| 3α,4β,12α-Trihydroxy-5β-cholan-24-oic acid | Lipid Pos | 92.93 | C24 H40 O5 | 1.990 | LMST04010077 |
| 3β,6α-diacetoxy-5α-pregn-20-ene | Lipid Pos | 89.75 | C26 H40 O3 | 2.265 | LMST02030215 |
| 4,4'-(Diphenylethenylidene)bis[N,N-dimethylbenzenamine] | Lipid Pos | 78.94 | C30 H30 N2 | 0.984 | KEGG: C15021 |
| 4,4-Difluoro-1α-hydroxyvitamin D3 | Lipid Pos | 87.31 | C27 H42 F2 O2 | 3.736 | LMST03020138 |
| 4,6-Nonadecanedione | Lipid Neg | 99.29 | C19 H36 O2 | 3.728 | HMDB35575 |
| 4,8 Dimethylnonanoyl carnitine | Lipid Neg | 97.12 | C18 H35 N O4 | 4.025 | HMDB06202 |
| 4-Amino-4-deoxy-alpha-L-arabinopyranosyl undecaprenyl phosphate | Lipid Pos | 90.42 | C60 H100 N O7 P | 3.284 | LMPR03020005 |
| 4-Dodecylbenzenesulfonic Acid | Lipid Neg | 98.19 | C18 H30 O3 S | 2.337 | HMDB59915 |
| 4-Ethyl-5-pentyloxazole | Lipid Neg | 80.44 | C10 H17 N O | 3.355 | HMDB37864 |
| 4-Keto lauric acid | Lipid Pos | 90.49 | C12 H22 O3 | 0.970 | LMFA01060039 |
| 4-Tetradecanamidobenzylphosphonic acid | Lipid Pos | 74.74 | C21 H36 N O4 P | 1.012 | CAS: 1096770-84-1 |
| 4Z,8Z,11Z,14Z-eicosatetraenoic acid | Lipid Neg | 98.06 | C20 H32 O2 | 2.788 | LMFA01030391 |
| 5,7-Dimethoxy-6-C-methylflavone | Lipid Pos | 82.49 | C18 H16 O4 | 1.083 | LMPK12110173 |
| 5,7-Heptadecadiene | Lipid Neg | 99.39 | C17 H32 | 3.360 | LMFA11000443 |
| 5,7-Pentadecadiene | Lipid Neg | 97.43 | C15 H28 | 2.679 | LMFA11000444 |
| 5,8,11,14-Docosatetraynoic acid | Lipid Neg | 80.42 | C22 H28 O2 | 2.647 | LMFA01030681 |
| 5,8,11-Eicosatrienoic acid | Lipid Neg | 99.30 | C20 H34 O2 | 3.082 | HMDB10378 |
| 5,9S,11R-trihydroxy-6E,14Z-prostadienoic acid-cyclo[8S,12R]-(d11) | Lipid Neg | 82.20 | C20 H23 D11 O5 | 2.099 | LMFA03110012 |
| 5-Acetyl-2,4-dimethylthiazole | Lipid Neg | 77.63 | C7 H9 N O S | 0.344 | HMDB41484 |
| 5E,8Z,11Z,14Z,16Z-eicosapentaenoic acid | Lipid Neg | 93.29 | C20 H30 O2 | 2.648 | LMFA01030397 |
| 5-Ethyl-3-methyl-2E,4E,6E-nonatriene | Lipid Pos | 80.44 | C12 H20 | 2.520 | LMFA11000051 |
| 5-Hydroxylysine | Lipid Pos | 70.12 | C6 H14 N2 O3 | 1.017 | HMDB00450 |
| 5-Hydroxy-p-mentha-6,8-dien-2-one | Lipid Neg | 99.49 | C10 H14 O2 | 1.280 | HMDB37011 |
| 5-Methyl-2-pentylthiazole | Lipid Pos | 71.40 | C9 H15 N S | 0.847 | HMDB40099 |
| 5Z,8Z,11Z,14Z-eicosatetraenoic acid | Lipid Neg | 82.13 | C20 H32 O2 | 3.366 | LMFA01030001 |
| 5Z-hexadecenoic acid | Lipid Neg | 99.65 | C16 H30 O2 | 2.680 | LMFA01030854 |
| 6-Hydroxydelphinidin 3-glucoside | Lipid Pos | 80.89 | C21 H21 O13 | 1.353 | LMPK12010431 |
| 6-Hydroxyluteolin 6,3'-dimethyl ether 7,4'-disulfate | Lipid Neg | 75.63 | C17 H14 O13 S2 | 1.674 | LMPK12111259 |
| 6-Keto-decanoylcarnitine | Lipid Pos | 70.27 | C17 H31 N O5 | 3.561 | HMDB13202 |
| 6-Methyltetrahydropterin | Lipid Pos | 86.31 | C7 H11 N5 O | 0.695 | HMDB02249 |
| 6-O-(Glcb)-(25R)-5alpha-spirostan-3beta,6alpha-diol | Lipid Neg | 78.90 | C34 H58 O9 | 5.471 | LMST01080087 |
| 6Z,9Z,11E-Heptadecatriene | Lipid Neg | 99.32 | C17 H30 | 2.860 | LMFA11000157 |
| 6α-Fluoropregn-4-ene-3,11,20-trione | Lipid Pos | 78.63 | C21 H27 F O3 | 1.031 | KEGG: C15327 |
| 6α-Hydroxycampestanol | Lipid Pos | 86.79 | C28 H50 O2 | 2.206 | KEGG: C15788 |
| 7,10,13,16,19-docosapentaenoic acid | Lipid Neg | 98.66 | C22 H34 O2 | 2.902 | LMFA01030184 |
| 7',8'-Dihydro-8'-hydroxyreticulataxanthin | Lipid Pos | 74.92 | C33 H46 O3 | 1.674 | HMDB39090 |
| 7-Ethyl-3,11-dimethyl-1,3Z,6E,10E-tridecatetraene | Lipid Neg | 85.03 | C17 H28 | 2.485 | LMFA11000124 |
| 7-Heptadecene | Lipid Neg | 99.36 | C17 H34 | 4.016 | LMFA11000505 |
| 7-Ketodeoxycholic acid | Lipid Pos | 89.25 | C24 H38 O5 | 1.663 | HMDB00391 |
| 7-O-Acetylaustroinulin | Lipid Neg | 80.71 | C22 H36 O4 | 3.854 | HMDB36804 |
| 7S,8S-DiHOME | Lipid Pos | 99.04 | C18 H34 O4 | 2.041 | KEGG: C07355 |
| 7Z,9Z,12Z-octadecatrienoic acid | Lipid Neg | 97.60 | C18 H30 O2 | 2.487 | LMFA01030346 |
| 8,24-Tritricontadiene | Aqueous | 70.16 | C33 H64 | 0.956 | LMFA11000527 |
| 8,24-Tritricontadiene | Aqueous | 70.60 | C33 H64 | 1.908 | LMFA11000527 |
| 8-[5]-ladderane-octanoic acid | Lipid Neg | 83.96 | C20 H30 O2 | 2.438 | LMFA01140004 |
| 8E-Dodecenyl acetate | Lipid Pos | 92.91 | C14 H26 O2 | 1.142 | LMFA07010227 |
| 8-Hydroxyalanylclavam | Aqueous | 98.92 | C8 H12 N2 O5 | 5.829 | KEGG: C17359 |
| 8-Methyl-3-hentriacontene | Lipid Neg | 98.83 | C32 H64 | 3.613 | HMDB35279 |
| 8Z,11Z,14Z,17Z,20Z-hexacosapentaenoic acid | Lipid Neg | 81.48 | C26 H42 O2 | 3.362 | LMFA01030845 |
| 8Z,11Z,14Z-heptadecatrienoic acid | Lipid Pos | 71.52 | C17 H28 O2 | 2.474 | KEGG: C16344 |
| 9,10,16-trihydroxy palmitic acid | Lipid Pos | 79.74 | C16 H32 O5 | 1.579 | LMFA01050101 |
| 9,12,14-octadecatrienoic acid | Lipid Neg | 81.75 | C18 H30 O2 | 3.228 | LMFA01030151 |
| 9,12-Hexadecadienylcarnitine | Lipid Pos | 72.74 | C25 H45 N O4 | 3.721 | LMFA07070009 |
| 9'-Carboxy-gamma-chromanol | Aqueous | 79.83 | C23 H36 O4 | 0.373 | HMDB12868 |
| 9'-Carboxy-gamma-tocotrienol | Lipid Neg | 97.29 | C23 H32 O4 | 2.791 | HMDB12869 |
| 9-cis-Retinoic acid | Lipid Neg | 94.12 | C20 H28 O2 | 2.237 | HMDB02369 |
| 9-Docosene | Aqueous | 80.45 | C22 H44 | 0.728 | LMFA11000546 |
| 9-Hydroxy-3-methoxypterocarpan | Lipid Neg | 84.77 | C16 H14 O4 | 0.414 | LMPK12070030 |
| 9-nitro-9E-octadecenoic acid | Lipid Neg | 96.94 | C18 H33 N O4 | 3.362 | LMFA01120004 |
| 9Z-octadecenyl 5Z,8Z,11Z,14Z-eicosatetraenoate | Lipid Neg | 91.31 | C38 H66 O2 | 6.215 | LMFA07010161 |
| Acetamide | Aqueous | 78.28 | C2 H5 N O | 0.537 | HMDB31645 |
| Acetylcarnitine | Lipid Neg | 81.22 | C9 H17 N O4 | 3.616 | HMDB00201 |
| Acetylcarnitine* | Aqueous | 99.32 | C9 H17 N O4 | 6.383 | HMDB00201 |
| Adrenic acid | Lipid Neg | 99.83 | C22 H36 O2 | 3.326 | HMDB02226 |
| Alanyl-Serine | Aqueous | 85.46 | C6 H12 N2 O4 | 7.476 | HMDB28696 |
| Aliskiren | Lipid Neg | 76.18 | C30 H53 N3 O6 | 3.037 | HMDB15387 |
| all-trans-8'-Apo-beta-carotenal | Lipid Pos | 74.40 | C30 H40 O | 1.968 | CAS: 1107-26-2 |
| Alpha-CEHC | Lipid Pos | 72.56 | C16 H22 O4 | 1.352 | HMDB01518 |
| Alpha-Linoleoylcholine | Lipid Neg | 91.45 | C23 H44 N O2 | 2.868 | HMDB13213 |
| Altretamine | Lipid Pos | 84.04 | C9 H18 N6 | 0.945 | HMDB14631 |
| Anandamide (20:1, n-9) | Lipid Pos | 99.09 | C22 H43 N O2 | 3.148 | LMFA08040010 |
| Anandamide (20:2, n-6) | Lipid Pos | 79.99 | C22 H41 N O2 | 2.716 | LMFA08040002 |
| Armillaric acid | Lipid Pos | 73.07 | C23 H28 O7 | 0.758 | HMDB37040 |
| Armillatin | Lipid Neg | 98.38 | C38 H58 O6 | 3.957 | HMDB38743 |
| Asp Asp Asp | Lipid Pos | 78.19 | C12 H17 N3 O10 | 1.354 | METLIN: 22750 |
| Aspartyl-threonine | Aqueous | 99.76 | C8 H14 N2 O6 | 6.589 | HMDB11169 |
| Aspartyl-threonine | Aqueous | 96.83 | C8 H14 N2 O6 | 10.336 | HMDB11169 |
| Azaspiracid | Lipid Neg | 91.57 | C47 H71 N O12 | 5.617 | HMDB33805 |
| Behenoyl-EA | Lipid Pos | 84.30 | C24 H49 N O2 | 3.716 | LMFA08040052 |
| Behenoyl-EA | Lipid Pos | 91.86 | C24 H49 N O2 | 4.499 | LMFA08040052 |
| Betaine* | Aqueous | 99.60 | C5 H11 N O2 | 4.102 | HMDB00043 |
| Bisnorcholic acid | Lipid Neg | 79.06 | C22 H36 O5 | 4.025 | HMDB02082 |
| Bisphenol A | Lipid Pos | 80.93 | C15 H16 O2 | 1.312 | HMDB32133 |
| Bradykinin | Lipid Neg | 94.23 | C50 H73 N15 O11 | 7.205 | HMDB04246 |
| C16 Sphinganine | Lipid Pos | 81.44 | C16 H35 N O2 | 1.310 | LMSP01040001 |
| C19 Sphingosine-1-phosphate | Lipid Pos | 94.97 | C19 H40 N O5 P | 0.999 | LMSP01050004 |
| C30 Monocyclic highly branched isoprenoid | Lipid Neg | 79.16 | C30 H60 | 3.231 | LMPR0106020003 |
| Caftaric acid | Aqueous | 83.80 | C13 H12 O9 | 6.630 | HMDB13680 |
| Calicoferol D | Lipid Neg | 81.31 | C28 H42 O2 | 4.725 | LMST03020313 |
| Candesartan | Lipid Neg | 80.64 | C24 H20 N6 O3 | 1.149 | HMDB14934 |
| Carnitine* | Aqueous | 96.94 | C7 H15 N O3 | 6.982 | HMDB00062 |
| CE(12:0) | Lipid Neg | 95.50 | C39 H68 O2 | 3.728 | HMDB02262 |
| CE(15:0) | Lipid Neg | 90.43 | C42 H74 O2 | 7.080 | HMDB60057 |
| CE(20:4(8Z,11Z,14Z,17Z)) | Lipid Neg | 87.44 | C47 H76 O2 | 2.874 | HMDB10371 |
| CE(22:0) | Lipid Pos | 86.54 | C49 H88 O2 | 3.283 | HMDB06727 |
| CE(22:4(7Z,10Z,13Z,16Z) | Lipid Neg | 84.62 | C49 H80 O2 | 3.615 | HMDB06729 |
| CE(22:4(7Z,10Z,13Z,16Z) | Lipid Neg | 85.46 | C49 H80 O2 | 3.229 | HMDB06729 |
| Ceramide (d18:0/18:1(11Z)) | Lipid Neg | 99.73 | C36 H71 N O3 | 6.801 | HMDB11762 |
| Ceramide (d18:1/16:0)* | Lipid Pos | 99.51 | C34 H67 N O3 | 5.732 | HMDB04949 |
| Ceramide (d18:1/18:0)* | Lipid Pos | 85.40 | C36 H71 N O3 | 6.296 | HMDB04950 |
| Ceramide (d18:1/24:0) | Lipid Pos | 90.86 | C42 H83 N O3 | 7.607 | HMDB04956 |
| Ceramide (d18:1/24:1) | Lipid Pos | 88.21 | C42 H81 N O3 | 7.228 | HMDB04953 |
| Ceramide(d14:1(4E)/22:0(2OH)) | Lipid Neg | 96.39 | C36 H71 N O4 | 6.225 | LMSP02010070 |
| Ceramide(d14:1(4E)/22:0(2OH)) | Lipid Neg | 97.36 | C36 H71 N O4 | 6.515 | LMSP02010070 |
| Ceramide(d14:1/26:0) | Lipid Neg | 98.86 | C40 H79 N O3 | 7.745 | LMSP02010042 |
| Ceramide(d16:1(4E)/22:0(2OH)) | Lipid Neg | 96.85 | C38 H75 N O4 | 6.794 | LMSP02010085 |
| Ceramide(d18:0/16:0) | Lipid Neg | 96.89 | C34 H69 N O3 | 6.449 | LMSP02020001 |
| Ceramide(d18:0/24:1(15Z)) | Lipid Neg | 98.60 | C42 H83 N O3 | 8.130 | LMSP02020011 |
| Ceramide(d18:1/16:0) | Lipid Pos | 99.51 | C34 H67 N O3 | 5.732 | LMSP02010004 |
| Ceramide(d18:1/18:0) | Lipid Pos | 96.67 | C36 H71 N O3 | 6.295 | LMSP02010006 |
| Ceramide(d18:1/23:0) | Lipid Neg | 93.91 | C41 H81 N O3 | 7.943 | LMSP02010021 |
| Ceramide(d18:1/24:1(15Z)) | Lipid Neg | 99.45 | C42 H81 N O3 | 7.730 | LMSP02010009 |
| Ceramide(d18:1/24:1(15Z)) | Lipid Pos | 87.08 | C42 H81 N O3 | 7.228 | LMSP02010009 |
| CerP(d18:1/16:0) | Lipid Neg | 99.17 | C34 H68 N O6 P | 5.519 | LMSP02050002 |
| Cetyl alcohol | Lipid Pos | 72.03 | C16 H34 O | 3.085 | LMFA05000061 |
| Cetyl alcohol | Lipid Pos | 96.45 | C16 H34 O | 1.597 | LMFA05000061 |
| Chenodeoxyglycocholate | Lipid Pos | 90.89 | C26 H43 N O5 | 3.172 | KEGG: C05466 |
| Cholest-5,24-dien-3beta-yl beta-D-glucopyranoside | Lipid Neg | 99.55 | C33 H54 O6 | 4.287 | LMST01010348 |
| Cholest-5-en-3beta-yl hydrogen sulfate | Lipid Neg | 98.54 | C27 H46 O4 S | 4.385 | LMST05020016 |
| Cholesterol* | Lipid Pos | 98.38 | C27 H46 O | 5.007 | KEGG: C00187 |
| Choline* | Aqueous | 87.11 | C5 H14 N O | 5.876 | KEGG: C00114 |
| cis-Jasmone | Lipid Pos | 89.40 | C11 H16 O | 10.484 | LMFA02020009 |
| CL(18:0/18:0/18:2/22:5) | Lipid Neg | 94.36 | C86 H154 O17 P2 | 5.728 | HMDB57006 |
| CL(18:1/18:1/18:1/18:2) | Lipid Neg | 96.26 | C82 H150 O17 P2 | 5.564 | HMDB57981 |
| CL(68:0) | Lipid Pos | 86.84 | C77 H150 O17 P2 | 3.404 | HMDB56487 |
| CL(70:2) | Lipid Pos | 74.73 | C80 H152 O17 P2 | 5.414 | HMDB56699 |
| Clemastine | Lipid Pos | 80.44 | C21 H26 Cl N O | 0.922 | CAS: 14976-57-9 |
| Coniferyl alcohol | Lipid Pos | 91.41 | C10 H12 O3 | 1.216 | KEGG: C00590 |
| Creatine* | Aqueous | 86.19 | C4 H9 N3 O2 | 4.491 | KEGG: C00300 |
| Creatinine* | Aqueous | 99.03 | C4 H7 N3 O | 0.836 | HMDB00562 |
| Darifenacin | Lipid Neg | 78.03 | C28 H30 N2 O2 | 3.725 | HMDB14639 |
| Deacetyl-O-demethyldiltiazem | Lipid Pos | 73.71 | C19 H22 N2 O3 S | 1.354 | CAS: 84903-82-2 |
| Debrisoquine | Lipid Neg | 83.57 | C10 H13 N3 | 2.689 | HMDB06543 |
| Decanamide | Lipid Pos | 87.93 | C10 H21 N O | 1.068 | LMFA08010005 |
| Decanoylcholine | Lipid Neg | 97.91 | C15 H32 N O2 | 3.614 | HMDB13228 |
| Decaprenoxanthin | Lipid Pos | 84.76 | C50 H72 | 2.952 | LMPR01080008 |
| Decylubiquinol | Lipid Pos | 74.33 | C19 H32 O4 | 1.529 | KEGG: C15495 |
| Dehydroascorbic acid | Lipid Neg | 83.86 | C6 H6 O6 | 0.569 | HMDB01264 |
| Dehydrocarpaine II | Lipid Neg | 94.86 | C28 H46 N2 O4 | 2.038 | HMDB30273 |
| Dehydrophytosphingosine | Lipid Pos | 84.24 | C18 H37 N O3 | 2.444 | LMSP01030002 |
| Dehydrotomatine | Lipid Neg | 94.61 | C50 H81 N O21 | 5.749 | HMDB32002 |
| Deoxodeoxydihydrogedunin | Lipid Pos | 93.66 | C28 H38 O5 | 3.172 | PubChem: 6708592 |
| Deoxymiroestrol | Lipid Pos | 99.05 | C20 H22 O5 | 1.220 | KEGG: C18164 |
| Desoxycorticosterone acetate | Lipid Pos | 73.13 | C23 H32 O4 | 1.032 | CAS: 56-47-3 |
| Deterrol stearate | Lipid Pos | 80.55 | C33 H50 O2 | 2.151 | HMDB34578 |
| DG(16:0/0:0/20:3n6) | Lipid Neg | 96.31 | C39 H70 O5 | 6.941 | HMDB56025 |
| DG(16:0/18:0/0:0) | Lipid Pos | 81.03 | C37 H72 O5 | 7.142 | HMDB07100 |
| DG(16:0/18:1(11Z)/0:0) | Lipid Pos | 77.07 | C37 H70 O5 | 6.740 | LMGL02010307 |
| DG(17:0/18:0/0:0) | Lipid Neg | 85.80 | C38 H74 O5 | 7.075 | LMGL02010025 |
| DG(18:0/0:0/18:0) | Lipid Pos | 85.16 | C39 H76 O5 | 7.516 | HMDB56036 |
| DG(18:0/15:0/0:0) | Lipid Pos | 78.14 | C36 H70 O5 | 6.951 | HMDB07155 |
| DG(18:0/16:0/0:0) | Lipid Pos | 95.23 | C37 H72 O5 | 7.141 | HMDB07156 |
| DG(18:3/18:4/0:0) | Lipid Pos | 85.28 | C39 H62 O5 | 2.013 | HMDB07309 |
| DG(20:0/15:0/0:0) | Lipid Pos | 91.83 | C38 H74 O5 | 7.342 | HMDB07358 |
| DG(20:2(11Z,14Z)/16:0/0:0) | Lipid Pos | 77.88 | C39 H72 O5 | 2.466 | HMDB07417 |
| DG(24:0/0:0/22:5n3) | Lipid Pos | 91.91 | C49 H86 O5 | 3.722 | HMDB56132 |
| DG(24:1(15Z)/16:1(9Z)/0:0) | Lipid Pos | 86.47 | C43 H80 O5 | 3.182 | HMDB07824 |
| DG(32:0) | Lipid Pos | 96.23 | C35 H68 O5 | 6.711 | LMGL02010009 |
| DG(33:2) | Lipid Pos | 90.92 | C36 H66 O5 | 1.353 | HMDB07074 |
| DG(34:0) | Lipid Pos | 96.13 | C37 H72 O5 | 7.141 | HMDB07020 |
| DG(34:1)* | Lipid Pos | 96.05 | C37 H70 O5 | 6.742 | HMDB07102 |
| DG(35:0) | Lipid Pos | 98.12 | C38 H74 O5 | 3.283 | HMDB55984 |
| DG(36:0) | Lipid Pos | 99.31 | C39 H76 O5 | 7.516 | HMDB07359 |
| DG(36:4) | Lipid Pos | 90.15 | C39 H68 O5 | 6.742 | HMDB07113 |
| DG(46:5) | Lipid Pos | 92.59 | C49 H86 O5 | 3.722 | HMDB07815 |
| DG(48:2) | Lipid Pos | 92.80 | C51 H96 O5 | 8.245 | HMDB07848 |
| Dibutyl adipate | Lipid Pos | 91.40 | C14 H26 O4 | 2.041 | CAS: 105-99-7 |
| Diethylphosphate | Lipid Pos | 93.85 | C4 H11 O4 P | 1.312 | HMDB12209 |
| Dihydro-2,4-dimethyl-6-(1-methylpropyl)-4H-1,3,5-dithiazine | Lipid Pos | 80.59 | C9 H19 N S2 | 1.031 | HMDB40336 |
| Dihydrothymine | Aqueous | 86.28 | C5 H8 N2 O2 | 5.833 | HMDB00079 |
| Dihydrozeatin | Lipid Pos | 92.40 | C10 H15 N5 O | 1.113 | HMDB12215 |
| Diisooctyl phthalate* | Lipid Pos | 77.19 | C24 H38 O4 | 3.173 | KEGG: C14577 |
| Dimethyl succinate | Aqueous | 98.47 | C6 H10 O4 | 1.031 | HMDB33837 |
| D-NMAPPD | Lipid Pos | 85.46 | C23 H38 N2 O5 | 0.598 | CAS: 35922-06-6 |
| Docosanamide | Lipid Pos | 92.30 | C22 H45 N O | 4.467 | HMDB00583 |
| Dodecyl propionate | Lipid Neg | 99.97 | C15 H30 O2 | 2.873 | HMDB32250 |
| Dolichotheline | Lipid Pos | 89.11 | C10 H17 N3 O | 2.519 | CAS: 23100-08-5 |
| Dryopteric acid | Aqueous | 82.13 | C17 H16 O8 | 6.718 | LMPK12020078 |
| Ecgonine | Lipid Neg | 79.53 | C9 H15 N O3 | 2.856 | HMDB06548 |
| Edetic Acid | Aqueous | 77.01 | C10 H16 N2 O8 | 6.713 | HMDB15109 |
| Edetic Acid | Aqueous | 98.34 | C10 H16 N2 O8 | 10.298 | HMDB15109 |
| Epiafzelechin Trimethyl Ether | Lipid Pos | 80.19 | C18 H20 O5 | 0.982 | PubChem: 6708630 |
| Epipregnanolone | Lipid Neg | 80.85 | C21 H34 O2 | 3.736 | HMDB01471 |
| Ergosta-5,7,22,24(28)-tetraen-3β-ol | Lipid Pos | 79.01 | C28 H42 O | 1.960 | LMST01031015 |
| Erythro-6,8-heptacosanediol | Lipid Pos | 93.39 | C27 H56 O2 | 7.623 | HMDB41071 |
| Estradiol-17α | Lipid Pos | 90.69 | C18 H24 O2 | 1.407 | LMST02010029 |
| Ethohexadiol | Lipid Pos | 84.47 | C8 H18 O2 | 0.790 | CAS: 94-96-2 |
| Ethyl 3-hydroxydodecanoate | Lipid Pos | 93.93 | C14 H28 O3 | 1.142 | HMDB59850 |
| Ethyl hexadecanoate | Lipid Neg | 98.55 | C18 H36 O2 | 4.019 | HMDB29811 |
| Ethylene glycol distearate | Lipid Neg | 96.08 | C38 H74 O4 | 3.362 | HMDB32260 |
| Falcarindione | Lipid Neg | 85.77 | C17 H20 O2 | 1.499 | HMDB33680 |
| Fasciculic acid A | Lipid Neg | 71.45 | C36 H60 O8 | 3.798 | HMDB36439 |
| Filfiline | Lipid Pos | 81.40 | C26 H47 N O | 4.833 | HMDB30953 |
| Flaccidin B | Lipid Pos | 80.91 | C41 H64 O12 | 6.159 | CAS: 117585-05-4 |
| Galactosylceramide (d18:1/16:0) | Lipid Neg | 99.42 | C40 H77 N O8 | 5.511 | HMDB10708 |
| Ganoderol A | Lipid Pos | 78.80 | C30 H46 O2 | 1.873 | HMDB35728 |
| Gibberellin A8 | Lipid Pos | 77.44 | C19 H24 O7 | 1.356 | KEGG: C03579 |
| Gingerol | Lipid Neg | 99.11 | C17 H26 O4 | 1.350 | HMDB05783 |
| GlcCer (d15:2(4E,6E)/18:0) | Lipid Neg | 87.05 | C39 H73 N O8 | 7.116 | LMSP0501AA58 |
| Glucose* | Aqueous | 99.77 | C6 H12 O6 | 0.861 | HMDB03345 |
| Glutarylcarnitine | Aqueous | 74.97 | C12 H21 N O6 | 8.420 | HMDB13130 |
| Glycerol 1-stearate* | Lipid Pos | 94.12 | C21 H42 O4 | 2.920 | PubChem: 24699 |
| Glycerophosphocholine | Aqueous | 98.39 | C8 H20 N O6 P | 7.678 | HMDB00086 |
| Glycyl-Threonine | Aqueous | 84.87 | C6 H12 N2 O4 | 10.217 | HMDB28851 |
| Gossypetin 3-O-sulfate | Lipid Neg | 79.03 | C15 H10 O11 S | 1.675 | LMPK12113267 |
| Guanadrel Sulfate | Aqueous | 98.33 | C10 H19 N3 O2 | 2.172 | HMDB14371 |
| Gymnodimine | Lipid Neg | 75.56 | C32 H45 N O4 | 1.864 | HMDB41430 |
| Heptadecan-2-ol | Lipid Pos | 95.47 | C17 H36 O | 1.580 | LMFA05000531 |
| Hericene A | Lipid Pos | 78.09 | C35 H56 O5 | 1.613 | HMDB41179 |
| Hericene B | Lipid Pos | 90.98 | C37 H58 O5 | 1.586 | HMDB41180 |
| Histidine | Aqueous | 82.03 | C6 H9 N3 O2 | 6.026 | HMDB00177 |
| Homodihydrocapsaicin | Lipid Neg | 80.29 | C19 H31 N O3 | 3.725 | HMDB36330 |
| Hordatine B | Lipid Neg | 91.76 | C29 H40 N8 O5 | 1.811 | HMDB30459 |
| Hydroxypropionic acid | Lipid Neg | 87.73 | C3 H6 O3 | 0.414 | HMDB00700 |
| Hypoxanthine* | Aqueous | 98.45 | C5 H4 N4 O | 0.564 | KEGG: C00262 |
| Iditol | Aqueous | 96.37 | C6 H14 O6 | 1.158 | HMDB11632 |
| Idoxuridine | Lipid Neg | 92.60 | C9 H11 I N2 O5 | 1.675 | HMDB14394 |
| Indoleacrylic acid | Aqueous | 83.51 | C11 H9 N O2 | 1.543 | HMDB00734 |
| Inosine 2',3'-cyclic phosphate | Aqueous | 90.84 | C10 H11 N4 O7 P | 6.631 | HMDB11680 |
| Isoamoritin | Lipid Pos | 73.30 | C31 H38 O6 | 1.783 | LMPK12140384 |
| Isoderricin A | Lipid Pos | 75.37 | C21 H22 O3 | 0.375 | LMPK12140014 |
| Isoleucine | Lipid Pos | 97.96 | C6 H13 N O2 | 0.514 | CAS: 443-79-8 |
| Isoliquiritigenin 4-methyl ether | Lipid Neg | 82.82 | C16 H14 O4 | 0.360 | LMPK12120097 |
| Isopentanol | Lipid Pos | 76.04 | C5 H12 O | 1.029 | HMDB06007 |
| Isopropyl 2-methylbutanoate | Lipid Neg | 87.50 | C8 H16 O2 | 1.279 | HMDB39217 |
| Kalkitoxin thioamide alcohol | Lipid Pos | 82.73 | C21 H40 N2 O2 S | 2.535 | METLIN: 65428 |
| k-Strophanthoside | Lipid Pos | 77.90 | C42 H64 O19 | 1.354 | CAS: 33279-57-1 |
| Kurilensoside F | Lipid Neg | 83.36 | C33 H58 O11 | 5.492 | LMST05050015 |
| LacCer(d14:0/18:0) | Aqueous | 89.87 | C44 H85 N O13 | 2.626 | LMSP0501AB23 |
| Lactaroviolin | Lipid Pos | 74.38 | C15 H14 O | 1.312 | HMDB35896 |
| L-alpha-Acetyl-N,N-dinormethadol | Lipid Pos | 80.06 | C21 H27 N O2 | 1.312 | CAS: 54276-34-5 |
| L-alpha-Amino-1H-pyrrole-1-hexanoic acid | Lipid Neg | 99.77 | C10 H16 N2 O2 | 1.266 | HMDB40551 |
| Lepidine C | Lipid Pos | 86.74 | C21 H20 N4 O2 | 1.216 | HMDB32717 |
| Leu Val Ile | Lipid Pos | 73.25 | C17 H33 N3 O4 | 1.312 | METLIN: 23602 |
| Leucine* | Aqueous | 87.27 | C6 H13 N O2 | 2.098 | HMDB00687 |
| Leupeptin | Aqueous | 98.97 | C20 H38 N6 O4 | 2.172 | CAS: 24125-16-4 |
| Linoleyl alcohol* | Lipid Pos | 89.22 | C18 H34 O | 2.921 | PubChem: 68168 |
| Lipid A -disaccharide-1-P | Lipid Pos | 78.08 | C68 H129 N2 O20 P | 6.156 | LMSL01040002 |
| Luteone (isoflavone) | Lipid Pos | 73.63 | C20 H18 O6 | 1.058 | LMPK12050287 |
| Lys Ala Lys | Lipid Pos | 83.14 | C15 H31 N5 O4 | 6.160 | METLIN: 20074 |
| Lys Gln Leu | Lipid Pos | 86.51 | C17 H33 N5 O5 | 1.000 | METLIN: 19534 |
| LysoPC(16:0)* | Lipid Pos | 97.25 | C24 H50 N O7 P | 1.598 | HMDB10382 |
| LysoPC(16:1) | Lipid Pos | 84.37 | C24 H48 N O7 P | 1.356 | HMDB10383 |
| LysoPC(17:0) | Lipid Neg | 99.43 | C25 H52 N O7 P | 2.418 | HMDB12108 |
| LysoPC(17:0) | Lipid Neg | 99.09 | C25 H52 N O7 P | 2.333 | HMDB12108 |
| LysoPC(18:0)* | Lipid Pos | 98.38 | C26 H54 N O7 P | 2.052 | LMGP01050026 |
| LysoPC(18:1)* | Lipid Pos | 85.73 | C26 H52 N O7 P | 1.676 | HMDB10385 |
| LysoPC(18:2) | Lipid Neg | 98.69 | C26 H50 N O7 P | 1.811 | HMDB10386 |
| LysoPC(18:2) | Lipid Neg | 99.03 | C26 H50 N O7 P | 1.748 | HMDB10386 |
| LysoPC(18:2) | Lipid Pos | 96.19 | C26 H50 N O7 P | 1.443 | HMDB10386 |
| LysoPC(18:3) | Lipid Pos | 84.82 | C26 H49 N O7 P | 1.600 | HMDB10387 |
| LysoPC(20:3(5Z,8Z,11Z)) | Aqueous | 81.30 | C28 H52 N O7 P | 3.776 | HMDB10393 |
| LysoPC(20:3(8Z,11Z,14Z)) | Lipid Neg | 98.62 | C28 H52 N O7 P | 1.923 | HMDB10394 |
| LysoPC(20:3(8Z,11Z,14Z)) | Lipid Neg | 98.63 | C28 H52 N O7 P | 1.869 | HMDB10394 |
| LysoPC(20:4(5Z,8Z,11Z,14Z)) | Lipid Pos | 84.37 | C28 H50 N O7 P | 1.397 | HMDB10395 |
| LysoPC(20:4(8Z,11Z,14Z,17Z)) | Lipid Neg | 98.92 | C28 H50 N O7 P | 1.781 | HMDB10396 |
| LysoPC(20:4(8Z,11Z,14Z,17Z)) | Lipid Pos | 86.24 | C28 H50 N O7 P | 1.416 | HMDB10396 |
| LysoPC(20:4) | Lipid Pos | 86.24 | C28 H50 N O7 P | 1.416 | HMDB10396 |
| LysoPC(20:5) | Lipid Pos | 89.57 | C28 H48 N O7 P | 1.438 | HMDB10397 |
| LysoPC(22:5) | Lipid Neg | 97.54 | C30 H52 N O7 P | 1.850 | HMDB10403 |
| LysoPC(22:6) | Aqueous | 83.23 | C30 H50 N O7 P | 3.700 | HMDB10404 |
| LysoPC(22:6) | Lipid Neg | 97.54 | C30 H50 N O7 P | 1.753 | HMDB10404 |
| LysoPE(0:0/18:1(11Z)) | Lipid Neg | 96.50 | C23 H46 N O7 P | 2.099 | HMDB11475 |
| LysoPE(0:0/20:3) | Lipid Neg | 77.45 | C25 H46 N O7 P | 1.657 | HMDB11486 |
| LysoPE(0:0/20:4) | Lipid Neg | 99.89 | C25 H44 N O7 P | 1.827 | HMDB11487 |
| LysoPE(0:0/22:4) | Lipid Neg | 98.95 | C27 H48 N O7 P | 1.780 | HMDB11493 |
| LysoPE(0:0/22:5) | Lipid Neg | 99.54 | C27 H46 N O7 P | 1.624 | HMDB11495 |
| LysoPE(0:0/22:6) | Lipid Neg | 79.49 | C27 H44 N O7 P | 1.786 | HMDB11496 |
| LysoPE(0:0/24:6) | Lipid Neg | 99.10 | C29 H48 N O7 P | 1.752 | HMDB11499 |
| LysoPE(16:0) | Lipid Pos | 82.77 | C21 H44 N O7 P | 1.624 | HMDB11473 |
| LysoPE(18:0)* | Lipid Pos | 94.05 | C23 H48 N O7 P | 2.076 | LMGP02050001 |
| LysoPE(18:0/0:0) | Lipid Neg | 98.70 | C23 H48 N O7 P | 1.884 | HMDB11130 |
| LysoPE(18:0/0:0) | Lipid Neg | 98.96 | C23 H48 N O7 P | 1.959 | HMDB11130 |
| LysoPE(18:0/0:0) | Lipid Neg | 99.69 | C23 H48 N O7 P | 2.483 | HMDB11130 |
| LysoPE(18:1(11Z)/0:0) | Lipid Neg | 99.49 | C23 H46 N O7 P | 1.714 | HMDB11505 |
| LysoPE(18:2(9Z,12Z)/0:0) | Lipid Neg | 82.19 | C23 H44 N O7 P | 1.789 | HMDB11507 |
| LysoPE(18:2(9Z,12Z)/0:0) | Lipid Neg | 98.91 | C23 H44 N O7 P | 1.844 | HMDB11507 |
| LysoPE(20:2(11Z,14Z)/0:0) | Lipid Neg | 99.61 | C25 H48 N O7 P | 1.803 | HMDB11513 |
| LysoPE(20:4(5Z,8Z,11Z,14Z)/0:0) | Lipid Pos | 73.45 | C25 H44 N O7 P | 1.435 | HMDB11517 |
| Mahuannin D | Lipid Pos | 74.07 | C30 H24 O9 | 7.816 | LMPK12030014 |
| Malaoxon | Lipid Neg | 70.90 | C10 H19 O7 P S | 1.675 | HMDB60627 |
| Melilotigenin | Lipid Neg | 98.02 | C30 H46 O5 | 2.216 | HMDB38737 |
| Menthone lactone | Lipid Pos | 90.47 | C10 H18 O2 | 1.142 | HMDB40330 |
| Methyl (7Z,9Z,9'Z)-6'-apo-y-caroten-6'-oate | Lipid Pos | 79.89 | C33 H44 O2 | 1.094 | HMDB31381 |
| Methyl 3b,24-dihydroxy-11,13(18)-oleanadien-30-oate | Lipid Neg | 76.95 | C31 H48 O4 | 2.423 | HMDB35257 |
| Methyl 3b-hydroxy-13(18)-oleanen-28-oate | Lipid Pos | 79.61 | C33 H52 O4 | 1.644 | HMDB35267 |
| Methyl hexadecanoic acid | Lipid Pos | 93.60 | C17 H34 O2 | 3.178 | HMDB61859 |
| Methyleugenol | Lipid Pos | 86.21 | C11 H14 O2 | 1.242 | CAS: 93-15-2 |
| MG(0:0/18:0/0:0) | Lipid Pos | 83.87 | C21 H42 O4 | 2.921 | HMDB11535 |
| MG(0:0/18:2(9Z,12Z)/0:0) | Lipid Pos | 72.69 | C21 H38 O4 | 1.984 | HMDB11538 |
| MG(0:0/18:3(9Z,12Z,15Z)/0:0) | Lipid Pos | 75.74 | C21 H36 O4 | 1.605 | HMDB11540 |
| MG(16:0) | Lipid Pos | 93.56 | C19 H38 O4 | 2.263 | LMGL01010009 |
| MG(18:0) | Lipid Pos | 98.72 | C21 H42 O4 | 2.921 | HMDB11131 |
| MG(18:0/0:0/0:0) | Lipid Neg | 99.27 | C21 H42 O4 | 3.358 | HMDB11131 |
| MG(18:0e/0:0/0:0) | Lipid Pos | 71.28 | C21 H44 O3 | 3.574 | HMDB11143 |
| MG(22:1(13Z)/0:0/0:0) | Aqueous | 72.22 | C25 H48 O4 | 1.257 | HMDB11582 |
| Modafinil acid | Aqueous | 79.06 | C15 H14 O3 S | 6.368 | HMDB61039 |
| Monocyclic botryococcane | Lipid Neg | 95.45 | C34 H68 | 4.024 | LMPR0106030003 |
| Myosmine | Lipid Pos | 70.22 | C9 H10 N2 | 1.354 | CAS: 532-12-7 |
| Myrtenol | Lipid Pos | 77.78 | C10 H16 O | 1.144 | KEGG: C11938 |
| N-(2R-hydroxy-15-methyl-3E-octadecenoyl)-4E,10E,12E-sphingatrienine | Lipid Neg | 85.99 | C37 H67 N O4 | 6.511 | LMSP02010095 |
| N-(3-(hexadecanoyloxy)-heptadecanoyl)-L-ornithine | Lipid Pos | 81.80 | C38 H74 N2 O5 | 6.807 | LMFA00000003 |
| N-(6-aminohexanoyl)-6-aminohexanoic acid | Lipid Pos | 80.89 | C12 H24 N2 O3 | 0.513 | LMFA00000009 |
| N-(tetradecanoyl)-deoxysphing-4-enine-1-sulfonate | Lipid Pos | 79.52 | C32 H63 N O5 S | 1.613 | LMSP00000001 |
| N,N-Diethylglycine | Lipid Pos | 98.62 | C6 H13 N O2 | 0.514 | KEGG: C16647 |
| N,N-dimethyl-Safingol | Lipid Pos | 82.39 | C20 H43 N O2 | 3.648 | LMSP01080056 |
| N-[[3-Hydroxy-2-(2-pentenyl)cyclopentyl]acetyl]isoleucine | Lipid Neg | 96.19 | C18 H31 N O4 | 2.860 | HMDB41248 |
| N2-Succinyl-L-glutamic acid 5-semialdehyde | Aqueous | 86.37 | C9 H13 N O6 | 6.877 | HMDB01180 |
| N3-Metyladenine | Lipid Pos | 80.92 | C6 H9 N5 | 0.790 | KEGG: C05026 |
| N6,N6,N6-Trimethyl-L-lysine | Lipid Pos | 80.95 | C9 H20 N2 O2 | 0.789 | HMDB01325 |
| N-Acetyl-b-glucosaminylamine | Lipid Neg | 78.58 | C8 H16 N2 O5 | 3.760 | HMDB01104 |
| N-cis-tetradec-9Z-enoyl-L-Homoserine lactone | Lipid Pos | 73.29 | C18 H31 N O3 | 1.312 | METLIN: 64725 |
| N-Cyclohexanecarbonylpentadecylamine | Aqueous | 91.90 | C22 H43 N O | 0.537 | CAS: 702638-84-4 |
| N-Desthienylethyl-rotigotine | Lipid Neg | 99.56 | C13 H19 N O | 1.815 | HMDB60843 |
| Nervonoyl-EA | Lipid Neg | 99.61 | C26 H51 N O2 | 3.728 | LMFA08040055 |
| NeuAcalpha2-3Galbeta-Ceramide (d18:1/16:0) | Lipid Neg | 95.08 | C51 H94 N2 O16 | 1.886 | LMSP0601AA01 |
| Neurine | Aqueous | 87.23 | C5 H13 N O | 2.099 | HMDB31259 |
| N-Heptanoylglycine | Lipid Neg | 81.51 | C9 H17 N O3 | 4.019 | HMDB13010 |
| N-Lactoyl ethanolamine | Lipid Pos | 72.78 | C5 H11 N O3 | 0.667 | HMDB32356 |
| N-Nitrososarcosine | Lipid Pos | 93.59 | C3 H6 N2 O3 | 0.515 | CAS: 13256-22-9 |
| N-octadecanoyl-valine | Lipid Neg | 96.96 | C23 H45 N O3 | 3.230 | LMFA08020122 |
| N-octadecanoyl-valine | Lipid Neg | 99.87 | C23 H45 N O3 | 4.166 | LMFA08020122 |
| N-octanoyl-L-Homoserine lactone | Lipid Pos | 70.75 | C12 H21 N O3 | 4.044 | CAS: 147852-84-4 |
| Nonane | Lipid Pos | 77.09 | C9 H20 | 1.142 | HMDB29595 |
| Nonanoylcarnitine | Lipid Neg | 90.69 | C16 H31 N O4 | 3.229 | HMDB13288 |
| Norajmaline | Lipid Pos | 74.86 | C20 H25 N O2 | 1.122 | KEGG: C11810 |
| N-palmitoyl leucine | Lipid Pos | 85.58 | C22 H43 N O3 | 1.900 | LMFA08020115 |
| N-Undecanoylglycine | Lipid Neg | 99.84 | C13 H25 N O3 | 1.047 | HMDB13286 |
| N-Undecylbenzenesulfonic acid | Lipid Neg | 97.78 | C17 H28 O3 S | 2.080 | HMDB32549 |
| O-acetyl-ADP-ribose | Lipid Pos | 72.45 | C17 H25 N5 O15 P2 | 1.029 | HMDB59656 |
| Obacunone | Lipid Pos | 72.52 | C26 H30 O7 | 1.053 | HMDB35858 |
| Octadecyl fumarate | Aqueous | 73.65 | C22 H40 O4 | 6.626 | HMDB38073 |
| Octadecyl fumarate | Lipid Pos | 77.16 | C22 H40 O4 | 2.329 | HMDB38073 |
| Octanal | Lipid Pos | 79.27 | C8 H16 O | 0.790 | LMFA06000028 |
| Octylamine | Lipid Pos | 92.84 | C8 H19 N | 0.439 | CAS: 111-86-4 |
| Oleoyl ethylamide* | Lipid Pos | 93.49 | C20 H39 N O | 2.966 | PubChem: 44270319 |
| O-octadecanoyl-R-carnitine | Lipid Neg | 98.58 | C25 H49 N O4 | 3.614 | LMFA07070008 |
| O-Octanoyl-R-carnitine | Lipid Neg | 98.50 | C15 H29 N O4 | 2.878 | LMFA07070002 |
| Oseltamivir | Lipid Neg | 91.84 | C16 H28 N2 O4 | 2.216 | HMDB14343 |
| Oxonantenine | Lipid Neg | 82.85 | C19 H13 N O5 | 0.563 | HMDB33367 |
| PA(14:1(9Z)/16:1(9Z)) | Lipid Pos | 73.78 | C33 H61 O8 P | 1.447 | LMGP10010113 |
| PA(17:1(9Z)/22:6) | Lipid Pos | 78.51 | C42 H69 O8 P | 6.159 | LMGP10010275 |
| PA(18:3/21:0) | Lipid Neg | 94.85 | C42 H77 O8 P | 5.286 | LMGP10010389 |
| PA(22:0/18:0) | Lipid Pos | 74.41 | C43 H85 O8 P | 3.721 | LMGP10010846 |
| PA(22:6/22:4) | Lipid Pos | 78.76 | C47 H73 O8 P | 1.000 | LMGP10010844 |
| PA(28:1) | Lipid Pos | 76.34 | C31 H59 O8 P | 1.470 | LMGP10010092 |
| PA(34:6) | Lipid Pos | 85.94 | C37 H61 O8 P | 1.337 | LMGP10010130 |
| PA(O-20:0/22:4(7Z,10Z,13Z,16Z)) | Lipid Neg | 86.78 | C45 H83 O7 P | 4.943 | LMGP10020069 |
| Palmitoylcarnitine | Aqueous | 86.72 | C23 H45 N O4 | 3.326 | HMDB00222 |
| Palmityl Trifluoromethyl Ketone | Lipid Pos | 97.49 | C17 H31 F3 O | 1.029 | CAS: 141022-99-3 |
| Panaxydol linoleate | Lipid Pos | 77.11 | C35 H54 O3 | 2.098 | HMDB41205 |
| Pangamic acid | Lipid Pos | 84.73 | C20 H40 N2 O8 | 1.575 | HMDB29949 |
| PC(13:0/0:0) | Lipid Neg | 99.66 | C21 H44 N O7 P | 2.008 | LMGP01050001 |
| PC(14:0/18:1(11Z)) | Lipid Neg | 98.66 | C40 H78 N O8 P | 5.733 | LMGP01010490 |
| PC(14:1(9Z)/20:1(11Z)) | Lipid Neg | 99.36 | C42 H80 N O8 P | 5.876 | LMGP01011398 |
| PC(15:0/0:0) | Lipid Neg | 76.51 | C23 H48 N O7 P | 1.781 | LMGP01050016 |
| PC(15:0/0:0) | Lipid Neg | 77.52 | C23 H48 N O7 P | 2.378 | LMGP01050016 |
| PC(15:0/20:3(5Z,8Z,11Z)) | Lipid Neg | 98.84 | C43 H80 N O8 P | 5.802 | HMDB07947 |
| PC(15:0/P-18:1(9Z)) | Lipid Neg | 97.43 | C41 H80 N O7 P | 6.823 | HMDB07964 |
| PC(15:1(9Z)/18:2(9Z,12Z)) | Lipid Neg | 88.49 | C41 H76 N O8 P | 5.892 | LMGP01011444 |
| PC(16:0/0:0) | Aqueous | 97.91 | C24 H50 N O7 P | 3.795 | LMGP01050018 |
| PC(16:0/0:0)[rac] | Lipid Neg | 99.70 | C24 H50 N O7 P | 1.960 | LMGP01050113 |
| PC(16:0/18:1(9E)) | Lipid Neg | 98.66 | C42 H82 N O8 P | 6.411 | LMGP01010581 |
| PC(16:0/3:0) | Aqueous | 85.99 | C27 H54 N O8 P | 3.695 | LMGP01010666 |
| PC(16:0/5:0(CHO)) | Lipid Neg | 89.23 | C29 H56 N O9 P | 2.156 | LMGP20010005 |
| PC(16:0/5:0(COOH)) | Aqueous | 80.99 | C29 H56 N O10 P | 3.732 | LMGP20010006 |
| PC(16:1(9E)/0:0) | Lipid Neg | 77.38 | C24 H48 N O7 P | 1.713 | LMGP01050021 |
| PC(16:1(9Z)/17:1(9Z)) | Lipid Neg | 99.00 | C41 H78 N O8 P | 6.398 | LMGP01011481 |
| PC(17:0/0:0) | Lipid Neg | 76.33 | C25 H52 N O7 P | 2.173 | LMGP01050024 |
| PC(17:0/16:1(9Z)) | Lipid Neg | 98.11 | C41 H80 N O8 P | 6.831 | LMGP01011502 |
| PC(17:0/17:1(9Z)) | Lipid Neg | 99.04 | C42 H82 N O8 P | 6.429 | LMGP01011503 |
| PC(17:1(10Z)/0:0) | Lipid Neg | 98.80 | C25 H50 N O7 P | 1.991 | LMGP01050002 |
| PC(17:2(9Z,12Z)/19:0) | Aqueous | 88.51 | C44 H84 N O8 P | 6.649 | LMGP01011567 |
| PC(17:2(9Z,12Z)/19:1(9Z)) | Lipid Neg | 97.48 | C44 H82 N O8 P | 6.066 | LMGP01011568 |
| PC(18:0/0:0) | Lipid Neg | 99.29 | C26 H54 N O7 P | 2.313 | LMGP01050026 |
| PC(18:0/0:0) | Lipid Neg | 99.41 | C26 H54 N O7 P | 2.424 | LMGP01050026 |
| PC(18:0/14:0) | Lipid Neg | 92.53 | C40 H80 N O8 P | 5.061 | HMDB08031 |
| PC(18:0/14:0) | Lipid Neg | 99.27 | C40 H80 N O8 P | 6.341 | HMDB08031 |
| PC(18:0/16:0) | Lipid Neg | 99.59 | C42 H84 N O8 P | 7.148 | HMDB08034 |
| PC(18:1(11Z)/16:1(9Z)) | Aqueous | 76.21 | C42 H80 N O8 P | 5.212 | LMGP01012147 |
| PC(18:1(11Z)/16:1(9Z)) | Aqueous | 85.65 | C42 H80 N O8 P | 2.678 | LMGP01012147 |
| PC(18:1(11Z)/18:1(11Z)) | Lipid Neg | 98.00 | C44 H84 N O8 P | 6.635 | LMGP01010841 |
| PC(18:1(11Z)/18:4(6Z,9Z,12Z,15Z)) | Lipid Neg | 97.07 | C44 H78 N O8 P | 5.302 | LMGP01012152 |
| PC(18:1(9Z)/22:0) | Lipid Neg | 88.77 | C48 H94 N O8 P | 7.416 | LMGP01010909 |
| PC(18:2(2E,4E)/0:0) | Lipid Neg | 98.28 | C26 H50 N O7 P | 1.750 | LMGP01050034 |
| PC(18:3(6Z,9Z,12Z)/18:1(9Z)) | Lipid Neg | 99.13 | C44 H80 N O8 P | 5.425 | LMGP01011652 |
| PC(18:3(6Z,9Z,12Z)/18:1(9Z)) | Lipid Neg | 99.28 | C44 H80 N O8 P | 5.741 | LMGP01011652 |
| PC(18:3(6Z,9Z,12Z)/22:5) | Lipid Neg | 95.20 | C48 H80 N O8 P | 5.181 | HMDB08187 |
| PC(18:4(6Z,9Z,12Z,15Z)/17:0) | Lipid Neg | 94.51 | C43 H78 N O8 P | 6.163 | LMGP01011708 |
| PC(19:0/0:0) | Lipid Neg | 77.77 | C27 H56 N O7 P | 2.712 | LMGP01050041 |
| PC(19:1(9Z)/14:1(9Z)) | Lipid Neg | 95.66 | C41 H78 N O8 P | 5.704 | LMGP01011759 |
| PC(19:3(10Z,13Z,16Z)/0:0) | Lipid Neg | 99.28 | C27 H50 N O7 P | 1.794 | LMGP01050003 |
| PC(2:0/O-18:1(9Z)) | Lipid Neg | 98.65 | C28 H56 N O7 P | 2.501 | LMGP01080006 |
| PC(20:1(11Z)/18:3(6Z,9Z,12Z)) | Aqueous | 81.18 | C46 H84 N O8 P | 2.751 | HMDB08304 |
| PC(20:1(11Z)/18:3(6Z,9Z,12Z)) | Aqueous | 90.29 | C46 H84 N O8 P | 2.527 | HMDB08304 |
| PC(20:1(11Z)/18:3(6Z,9Z,12Z)) | Lipid Neg | 99.01 | C46 H84 N O8 P | 6.454 | LMGP01011819 |
| PC(20:2(11Z,14Z)/16:1(9Z)) | Aqueous | 75.18 | C44 H82 N O8 P | 2.494 | HMDB08332 |
| PC(20:2(11Z,14Z)/18:1(11Z)) | Aqueous | 94.15 | C46 H86 N O8 P | 2.715 | HMDB08334 |
| PC(20:2(11Z,14Z)/18:3(6Z,9Z,12Z)) | Aqueous | 71.58 | C46 H82 N O8 P | 2.399 | HMDB08337 |
| PC(20:2(11Z,14Z)/18:4(6Z,9Z,12Z,15Z)) | Lipid Neg | 98.31 | C46 H80 N O8 P | 5.424 | LMGP01011851 |
| PC(20:3(5Z,8Z,11Z)/20:3(8Z,11Z,14Z)) | Aqueous | 73.19 | C48 H84 N O8 P | 2.580 | HMDB08377 |
| PC(20:3(5Z,8Z,11Z)/20:4(8Z,11Z,14Z,17Z)) | Lipid Neg | 95.82 | C48 H82 N O8 P | 5.665 | HMDB08379 |
| PC(20:3(5Z,8Z,11Z)/P-18:1(9Z)) | Lipid Neg | 95.95 | C46 H84 N O7 P | 6.311 | HMDB08393 |
| PC(20:3(8Z,11Z,14Z)/14:0) | Lipid Neg | 98.00 | C42 H78 N O8 P | 5.581 | LMGP01011868 |
| PC(20:3(8Z,11Z,14Z)/P-18:1(9Z)) | Lipid Neg | 98.86 | C46 H84 N O7 P | 6.306 | HMDB08425 |
| PC(20:4(5Z,8Z,11Z,14Z)/15:0) | Lipid Neg | 99.42 | C43 H78 N O8 P | 6.325 | LMGP01011901 |
| PC(20:4(5Z,8Z,11Z,14Z)/20:0) | Lipid Neg | 96.78 | C48 H88 N O8 P | 6.978 | LMGP01011915 |
| PC(20:5(5Z,8Z,11Z,14Z,17Z)/15:1(9Z)) | Lipid Neg | 99.09 | C43 H74 N O8 P | 5.581 | LMGP01011931 |
| PC(20:5(5Z,8Z,11Z,14Z,17Z)/15:1(9Z)) | Lipid Neg | 99.48 | C43 H74 N O8 P | 5.370 | LMGP01011931 |
| PC(20:5(5Z,8Z,11Z,14Z,17Z)/20:1(11Z)) | Lipid Neg | 95.62 | C48 H84 N O8 P | 6.254 | HMDB08505 |
| PC(20:5/20:3) | Lipid Neg | 99.09 | C48 H80 N O8 P | 5.172 | LMGP01011947 |
| PC(22:1(11Z)/18:3(6Z,9Z,12Z)) | Lipid Neg | 92.35 | C48 H88 N O8 P | 6.979 | LMGP01012018 |
| PC(22:1(13Z)/14:1(9Z)) | Aqueous | 88.33 | C44 H84 N O8 P | 6.642 | LMGP01012219 |
| PC(22:4(7Z,10Z,13Z,16Z)/0:0) | Lipid Neg | 75.11 | C30 H54 N O7 P | 2.065 | LMGP01050124 |
| PC(22:5/20:5) | Lipid Neg | 96.49 | C50 H80 N O8 P | 5.040 | HMDB08675 |
| PC(22:5(7Z,10Z,13Z,16Z,19Z)/16:0) | Lipid Neg | 96.37 | C46 H82 N O8 P | 5.787 | HMDB08692 |
| PC(22:5(7Z,10Z,13Z,16Z,19Z)/18:0) | Lipid Neg | 95.79 | C48 H86 N O8 P | 6.493 | HMDB08694 |
| PC(22:5(7Z,10Z,13Z,16Z,19Z)/18:1(11Z)) | Lipid Neg | 98.92 | C48 H84 N O8 P | 6.254 | HMDB08695 |
| PC(6:2(3E,5E)/14:2(11E,13E)) | Lipid Neg | 72.12 | C28 H48 N O8 P | 1.807 | LMGP01011236 |
| PC(O-12:0/O-2:0) | Lipid Pos | 79.86 | C22 H48 N O6 P | 1.000 | LMGP01040017 |
| PC(O-14:0/2:0) | Aqueous | 80.06 | C24 H50 N O7 P | 3.764 | LMGP01020019 |
| PC(O-14:0/2:0) | Lipid Neg | 99.11 | C24 H50 N O7 P | 1.894 | LMGP01020019 |
| PC(O-14:0/2:0) | Lipid Neg | 99.32 | C24 H50 N O7 P | 1.958 | LMGP01020019 |
| PC(O-14:1(1E)/0:0) | Lipid Neg | 95.36 | C22 H46 N O6 P | 2.000 | LMGP01070001 |
| PC(O-15:0/0:0) | Lipid Neg | 98.17 | C23 H50 N O6 P | 2.169 | LMGP01060009 |
| PC(O-16:0/2:0) | Lipid Neg | 99.24 | C26 H54 N O7 P | 2.422 | LMGP01020046 |
| PC(O-18:0/1:0) | Lipid Neg | 98.98 | C27 H56 N O7 P | 2.712 | LMGP01020084 |
| PC(O-18:1(11Z)/0:0) | Lipid Neg | 77.70 | C26 H54 N O6 P | 2.259 | LMGP01060034 |
| PC(P-16:0/2:0) | Lipid Neg | 99.43 | C26 H52 N O7 P | 2.049 | LMGP01030009 |
| PC(P-18:0/18:3(9Z,12Z,15Z)) | Lipid Neg | 98.78 | C44 H82 N O7 P | 6.271 | LMGP01030060 |
| PC(P-18:0/20:5(5Z,8Z,11Z,14Z,17Z)) | Lipid Neg | 94.49 | C46 H82 N O7 P | 6.061 | HMDB11255 |
| PC(P-18:1(11Z)/15:0) | Lipid Neg | 93.64 | C41 H80 N O7 P | 6.745 | HMDB11271 |
| PE(12:0/18:0) | Lipid Neg | 98.99 | C35 H70 N O8 P | 5.540 | LMGP02011261 |
| PE(14:0/16:0) | Lipid Pos | 98.07 | C35 H70 N O8 P | 5.077 | LMGP02010302 |
| PE(16:0/0:0) | Lipid Neg | 82.76 | C21 H44 N O7 P | 1.934 | LMGP02050002 |
| PE(16:1(9Z)/18:0) | Lipid Neg | 98.80 | C39 H76 N O8 P | 6.253 | LMGP02010524 |
| PE(17:0/0:0) | Lipid Neg | 80.87 | C22 H46 N O7 P | 1.638 | LMGP02050030 |
| PE(17:0/17:2(9Z,12Z)) | Lipid Neg | 96.08 | C39 H74 N O8 P | 5.791 | LMGP02010545 |
| PE(17:2(9Z,12Z)/18:0) | Lipid Neg | 98.01 | C40 H76 N O8 P | 5.227 | LMGP02010601 |
| PE(18:0/19:0) | Lipid Neg | 98.85 | C42 H84 N O8 P | 7.149 | LMGP02010630 |
| PE(18:3(6Z,9Z,12Z)/14:1(9Z)) | Lipid Pos | 75.29 | C37 H66 N O8 P | 2.829 | HMDB09119 |
| PE(20:3(8Z,11Z,14Z)/P-16:0) | Lipid Neg | 98.49 | C41 H76 N O7 P | 6.172 | HMDB09378 |
| PE(20:3(8Z,11Z,14Z)/P-18:1(9Z)) | Lipid Neg | 94.92 | C43 H78 N O7 P | 6.593 | HMDB09381 |
| PE(20:4/22:6) | Lipid Neg | 97.81 | C47 H74 N O8 P | 5.105 | HMDB09408 |
| PE(20:4(8Z,11Z,14Z,17Z)/16:0) | Lipid Neg | 98.53 | C41 H74 N O8 P | 5.719 | HMDB09418 |
| PE(20:4(8Z,11Z,14Z,17Z)/18:1(11Z)) | Lipid Neg | 99.02 | C43 H76 N O8 P | 5.802 | HMDB09421 |
| PE(20:5(5Z,8Z,11Z,14Z,17Z)/20:2(11Z,14Z)) | Lipid Neg | 96.51 | C45 H76 N O8 P | 5.661 | HMDB09462 |
| PE(21:0/0:0) | Aqueous | 97.37 | C26 H54 N O7 P | 3.778 | LMGP02050026 |
| PE(22:2(13Z,16Z)/20:5(5Z,8Z,11Z,14Z,17Z)) | Aqueous | 91.08 | C47 H80 N O8 P | 2.427 | HMDB09566 |
| PE(22:4(7Z,10Z,13Z,16Z)/P-16:0) | Lipid Neg | 98.79 | C43 H78 N O7 P | 6.588 | HMDB09609 |
| PE(22:5(7Z,10Z,13Z,16Z,19Z)/dm18:1(9Z)) | Lipid Neg | 97.08 | C45 H78 N O7 P | 6.446 | HMDB09678 |
| PE(30:0) | Lipid Pos | 98.09 | C35 H70 N O8 P | 5.077 | LMGP02010302 |
| PE(32:3) | Lipid Pos | 88.36 | C37 H68 N O8 P | 5.080 | LMGP02010681 |
| PE(38:5) | Lipid Pos | 90.19 | C43 H76 N O8 P | 5.380 | HMDB09324 |
| PE(40:5) | Lipid Pos | 81.74 | C45 H80 N O8 P | 5.958 | HMDB09586 |
| PE(O-18:0/17:0) | Lipid Neg | 96.50 | C40 H82 N O7 P | 7.051 | LMGP02020044 |
| PE(P-16:0/20:4(8Z,11Z,14Z,17Z)) | Lipid Neg | 98.25 | C41 H74 N O7 P | 6.016 | HMDB11353 |
| PE(P-18:0/18:2(9Z,12Z)) | Lipid Neg | 98.60 | C41 H78 N O7 P | 6.747 | HMDB11376 |
| PE(P-18:0/20:3(5Z,8Z,11Z)) | Lipid Neg | 85.48 | C43 H80 N O7 P | 6.416 | HMDB11383 |
| PE(P-18:0/20:3(5Z,8Z,11Z)) | Lipid Neg | 98.25 | C43 H80 N O7 P | 6.716 | HMDB11383 |
| PE(P-18:0/22:4(7Z,10Z,13Z,16Z)) | Lipid Neg | 90.18 | C45 H82 N O7 P | 6.974 | HMDB11391 |
| PE(P-18:1(11Z)/16:1(9Z)) | Lipid Neg | 98.50 | C39 H74 N O7 P | 6.109 | HMDB11405 |
| PE(P-18:1(11Z)/20:0) | Lipid Neg | 95.96 | C43 H84 N O7 P | 7.351 | HMDB11413 |
| PE(P-18:1(11Z)/20:5(5Z,8Z,11Z,14Z,17Z)) | Lipid Neg | 98.82 | C43 H74 N O7 P | 5.866 | HMDB11420 |
| PE(P-18:1(11Z)/22:6(4Z,7Z,10Z,13Z,16Z,19Z)) | Lipid Neg | 90.98 | C45 H76 N O7 P | 5.946 | HMDB11427 |
| PE(P-18:1(9Z)/20:4(8Z,11Z,14Z,17Z)) | Lipid Neg | 99.68 | C43 H76 N O7 P | 6.230 | HMDB11452 |
| PE(P-18:1(9Z)/20:4(8Z,11Z,14Z,17Z)) | Lipid Neg | 99.76 | C43 H76 N O7 P | 5.991 | HMDB11452 |
| PE-Cer(d14:1(4E)/22:0) | Lipid Neg | 98.88 | C38 H77 N2 O6 P | 4.929 | LMSP03020008 |
| PE-Cer(d14:1(4E)/23:0) | Lipid Neg | 94.18 | C39 H79 N2 O6 P | 5.279 | LMSP03020010 |
| PE-Cer(d15:1(4E)/22:0) | Aqueous | 89.15 | C39 H79 N2 O6 P | 3.580 | LMSP03020017 |
| PE-Cer(d16:1(4E)/20:0) | Lipid Neg | 99.04 | C38 H77 N2 O6 P | 4.929 | LMSP03020022 |
| PE-Cer(d16:1(4E)/21:0) | Aqueous | 91.56 | C39 H79 N2 O6 P | 3.591 | LMSP03020023 |
| PE-Cer(d16:1(4E)/21:0) | Lipid Neg | 99.36 | C39 H79 N2 O6 P | 5.276 | LMSP03020023 |
| PE-Cer(d16:2(4E,6E)/21:0) | Lipid Neg | 98.84 | C39 H77 N2 O6 P | 4.715 | LMSP03020057 |
| Pelargonic acid | Lipid Pos | 73.73 | C9 H18 O2 | 1.190 | HMDB00847 |
| Peltatol A | Lipid Pos | 73.96 | C42 H58 O4 | 1.029 | HMDB38701 |
| Pentadecylic acid | Lipid Pos | 85.36 | C15 H30 O2 | 2.459 | LMFA01010015 |
| Perfluorooctanesulfonic acid | Lipid Neg | 78.36 | C8 H F17 O3 S | 1.409 | HMDB59586 |
| Perflutren | Lipid Pos | 81.88 | C3 F8 | 10.455 | HMDB14696 |
| PG(14:0/15:1(9Z)) | Lipid Pos | 73.71 | C35 H67 O10 P | 1.427 | LMGP04010095 |
| PG(22:6(4Z,7Z,10Z,13Z,16Z,19Z)/0:0) | Lipid Neg | 76.88 | C28 H45 O9 P | 4.400 | LMGP04050016 |
| PG(32:0) | Lipid Pos | 88.27 | C38 H75 O10 P | 6.154 | LMGP04010929 |
| PG(O-20:0/17:0) | Lipid Pos | 71.34 | C43 H87 O9 P | 3.718 | LMGP04020048 |
| PG(O-20:0/18:1(9Z)) | Lipid Neg | 95.16 | C44 H87 O9 P | 6.376 | LMGP04020052 |
| PG(P-18:0/17:1(9Z)) | Lipid Neg | 94.74 | C41 H79 O9 P | 5.392 | LMGP04030037 |
| PG(P-18:0/17:2(9Z,12Z)) | Lipid Neg | 99.13 | C41 H77 O9 P | 5.192 | LMGP04030038 |
| PG(P-18:0/19:0) | Lipid Neg | 83.95 | C43 H85 O9 P | 6.393 | LMGP04030044 |
| Phenylalanine* | Aqueous | 99.36 | C9 H11 N O2 | 1.786 | HMDB00159 |
| Phenylgalactoside | Lipid Pos | 70.40 | C12 H16 O6 | 1.140 | KEGG: C02578 |
| Phosphoguanidinoacetate | Aqueous | 71.44 | C3 H8 N3 O5 P | 3.776 | HMDB03705 |
| Phthalic acid* | Lipid Pos | 76.67 | C8 H6 O4 | 3.177 | HMDB02107 |
| Phyllohydroquinone | Lipid Pos | 86.06 | C31 H48 O2 | 2.206 | LMPR02030030 |
| PI(14:0/20:2(11Z,14Z)) | Lipid Neg | 99.39 | C43 H79 O13 P | 5.325 | LMGP06010071 |
| PI(16:1(9Z)/20:4(5Z,8Z,11Z,14Z)) | Lipid Neg | 95.92 | C45 H77 O13 P | 5.327 | LMGP06010870 |
| PI(16:1(9Z)/22:4(7Z,10Z,13Z,16Z)) | Lipid Neg | 99.58 | C47 H81 O13 P | 5.278 | LMGP06010193 |
| PI(16:1(9Z)/22:6(4Z,7Z,10Z,13Z,16Z,19Z)) | Lipid Neg | 97.83 | C47 H77 O13 P | 5.195 | LMGP06010869 |
| PI(16:2(9Z,12Z)/22:3(10Z,13Z,16Z)) | Lipid Neg | 99.28 | C47 H81 O13 P | 5.278 | HMDB09804 |
| PI(18:2(9Z,12Z)/0:0) | Lipid Neg | 99.29 | C27 H49 O12 P | 1.697 | LMGP06050010 |
| PI(20:0/20:3(8Z,11Z,14Z)) | Lipid Neg | 99.66 | C49 H89 O13 P | 4.536 | LMGP06010490 |
| PI(20:2(11Z,14Z)/18:2(9Z,12Z)) | Aqueous | 90.87 | C47 H83 O13 P | 0.803 | HMDB09877 |
| PI(20:2(11Z,14Z)/18:2(9Z,12Z)) | Lipid Neg | 99.41 | C47 H83 O13 P | 5.921 | HMDB09877 |
| PI(20:3(8Z,11Z,14Z)/18:3(6Z,9Z,12Z)) | Lipid Neg | 97.52 | C47 H79 O13 P | 5.040 | LMGP06010571 |
| PI(20:3(8Z,11Z,14Z)/21:0) | Lipid Neg | 70.67 | C50 H91 O13 P | 1.795 | LMGP06010582 |
| PI(20:4(5Z,8Z,11Z,14Z)/0:0) | Lipid Neg | 99.13 | C29 H49 O12 P | 1.682 | LMGP06050006 |
| PI(20:4(5Z,8Z,11Z,14Z)/20:4(5Z,8Z,11Z,14Z)) | Lipid Neg | 93.64 | C49 H79 O13 P | 5.271 | LMGP06010827 |
| PI(20:5(5Z,8Z,11Z,14Z,17Z)/20:2(11Z,14Z)) | Lipid Neg | 80.47 | C49 H81 O13 P | 5.946 | LMGP06010637 |
| PI(21:0/20:2(11Z,14Z)) | Lipid Pos | 71.62 | C50 H93 O13 P | 1.029 | LMGP06010663 |
| PI(40:3) | Lipid Pos | 94.14 | C49 H89 O13 P | 4.044 | LMGP06010490 |
| PI(O-16:0/14:0) | Lipid Pos | 77.61 | C39 H77 O12 P | 1.014 | LMGP06020004 |
| PI(O-18:0/12:0) | Lipid Pos | 96.33 | C39 H77 O12 P | 1.005 | LMGP06020019 |
| PI(O-30:0) | Lipid Pos | 97.04 | C39 H77 O12 P | 1.000 | LMGP06020004 |
| PI(P-34:1) | Lipid Pos | 96.67 | C43 H81 O12 P | 3.172 | LMGP06030036 |
| Pimaric acid | Lipid Pos | 73.59 | C20 H30 O2 | 2.242 | CAS: 79-54-9 |
| Pipercide | Lipid Neg | 99.33 | C22 H29 N O3 | 2.239 | HMDB33449 |
| Pirimicarb | Aqueous | 82.78 | C11 H18 N4 O2 | 0.594 | CAS: 23103-98-2 |
| Polyethylene glycol | Lipid Neg | 82.66 | C9 H17 N O3 | 4.016 | HMDB37790 |
| Pregabalin | Aqueous | 80.41 | C8 H17 N O2 | 9.440 | HMDB14375 |
| Presqualene diphosphate | Lipid Neg | 88.05 | C30 H52 O7 P2 | 1.960 | HMDB01278 |
| Proline betaine | Aqueous | 93.71 | C7 H13 N O2 | 4.262 | KEGG: C10172 |
| Proline betaine | Lipid Pos | 90.36 | C7 H13 N O2 | 2.468 | HMDB04827 |
| Prolyl-2-naphthylamide | Lipid Pos | 78.20 | C15 H16 N2 O | 10.483 | KEGG: C03305 |
| Propane-1,2-diol 1-phosphate | Aqueous | 76.48 | C3 H9 O5 P | 3.772 | KEGG: C03894 |
| Prostanoic acid skeleton | Lipid Neg | 99.66 | C20 H38 O2 | 4.115 | LMFA03010000 |
| PS(13:0/22:2(13Z,16Z)) | Lipid Neg | 96.56 | C41 H76 N O10 P | 5.608 | LMGP03010090 |
| PS(16:1(9Z)/19:0) | Lipid Neg | 99.09 | C41 H78 N O10 P | 5.216 | LMGP03010214 |
| PS(17:0/19:0) | Lipid Neg | 92.25 | C42 H82 N O10 P | 6.152 | LMGP03010237 |
| PS(17:2(9Z,12Z)/22:2(13Z,16Z)) | Lipid Neg | 98.22 | C45 H80 N O10 P | 4.967 | LMGP03010305 |
| PS(18:0/19:1(9Z)) | Lipid Neg | 99.93 | C43 H82 N O10 P | 5.863 | LMGP03010318 |
| PS(18:0/20:3(8Z,11Z,14Z)) | Lipid Neg | 78.59 | C44 H80 N O10 P | 5.394 | HMDB12382 |
| PS(19:0/0:0) | Lipid Neg | 79.87 | C25 H50 N O9 P | 1.714 | LMGP03050028 |
| PS(19:0/16:0) | Lipid Neg | 100.00 | C41 H80 N O10 P | 5.722 | LMGP03010953 |
| PS(19:0/20:2(11Z,14Z)) | Lipid Neg | 95.89 | C45 H84 N O10 P | 7.156 | LMGP03010470 |
| PS(19:0/20:2(11Z,14Z)) | Lipid Neg | 99.77 | C45 H84 N O10 P | 6.058 | LMGP03010470 |
| PS(19:0/20:3(8Z,11Z,14Z)) | Lipid Neg | 95.42 | C45 H82 N O10 P | 6.431 | LMGP03010471 |
| PS(19:0/20:3(8Z,11Z,14Z)) | Lipid Neg | 96.82 | C45 H82 N O10 P | 5.420 | LMGP03010471 |
| PS(19:0/20:3(8Z,11Z,14Z)) | Lipid Neg | 99.98 | C45 H82 N O10 P | 5.738 | LMGP03010471 |
| PS(20:2(11Z,14Z)/0:0) | Lipid Neg | 76.19 | C26 H48 N O9 P | 2.485 | LMGP03050021 |
| PS(20:2(11Z,14Z)/17:0) | Lipid Neg | 98.46 | C43 H80 N O10 P | 5.540 | LMGP03010566 |
| PS(20:2(11Z,14Z)/17:0) | Lipid Neg | 99.31 | C43 H80 N O10 P | 5.339 | LMGP03010566 |
| PS(20:2(11Z,14Z)/17:1(9Z)) | Lipid Neg | 98.51 | C43 H78 N O10 P | 5.041 | LMGP03010567 |
| PS(20:2(11Z,14Z)/19:1(9Z)) | Lipid Neg | 98.61 | C45 H82 N O10 P | 5.420 | LMGP03010576 |
| PS(20:2(11Z,14Z)/20:3(8Z,11Z,14Z)) | Lipid Neg | 97.41 | C46 H80 N O10 P | 5.241 | LMGP03010580 |
| PS(20:2(11Z,14Z)/22:6(4Z,7Z,10Z,13Z,16Z,19Z)) | Lipid Neg | 77.52 | C48 H78 N O10 P | 6.178 | LMGP03010588 |
| PS(20:3(8Z,11Z,14Z)/21:0) | Lipid Neg | 99.48 | C47 H86 N O10 P | 6.438 | LMGP03010614 |
| PS(20:4(5Z,8Z,11Z,14Z)/0:0) | Lipid Neg | 78.26 | C26 H44 N O9 P | 1.847 | LMGP03050007 |
| PS(20:4(5Z,8Z,11Z,14Z)/21:0) | Lipid Neg | 99.07 | C47 H84 N O10 P | 5.804 | LMGP03010643 |
| PS(20:5(5Z,8Z,11Z,14Z,17Z)/19:0) | Lipid Neg | 88.63 | C45 H78 N O10 P | 5.376 | LMGP03010664 |
| PS(20:5(5Z,8Z,11Z,14Z,17Z)/21:0) | Lipid Neg | 97.78 | C47 H82 N O10 P | 5.573 | LMGP03010672 |
| PS(20:5(5Z,8Z,11Z,14Z,17Z)/21:0) | Lipid Neg | 98.89 | C47 H82 N O10 P | 5.318 | LMGP03010672 |
| PS(22:0/13:0) | Lipid Neg | 99.21 | C41 H80 N O10 P | 5.721 | LMGP03010849 |
| PS(22:0/15:0) | Lipid Neg | 99.16 | C43 H84 N O10 P | 6.415 | LMGP03010703 |
| PS(22:0/16:0) | Lipid Neg | 98.26 | C44 H86 N O10 P | 6.794 | LMGP03010705 |
| PS(22:0/17:0) | Lipid Neg | 97.40 | C45 H88 N O10 P | 7.192 | LMGP03010707 |
| PS(22:0/17:1(9Z)) | Lipid Neg | 99.87 | C45 H86 N O10 P | 6.607 | LMGP03010708 |
| PS(22:1(11Z)/16:0) | Lipid Neg | 80.55 | C44 H84 N O10 P | 6.250 | LMGP03010731 |
| PS(22:2(13Z,16Z)/0:0) | Aqueous | 81.87 | C28 H52 N O9 P | 3.826 | LMGP03050024 |
| PS(22:2(13Z,16Z)/17:2(9Z,12Z)) | Lipid Neg | 93.56 | C45 H80 N O10 P | 5.298 | LMGP03010766 |
| PS(22:2(13Z,16Z)/17:2(9Z,12Z)) | Lipid Neg | 94.73 | C45 H80 N O10 P | 5.885 | LMGP03010766 |
| PS(22:2(13Z,16Z)/17:2(9Z,12Z)) | Lipid Neg | 98.50 | C45 H80 N O10 P | 5.222 | LMGP03010766 |
| PS(22:2(13Z,16Z)/19:1(9Z)) | Lipid Neg | 99.16 | C47 H86 N O10 P | 6.151 | LMGP03010774 |
| PS(22:2(13Z,16Z)/20:2(11Z,14Z)) | Lipid Neg | 97.36 | C48 H86 N O10 P | 7.011 | LMGP03010777 |
| PS(22:4(7Z,10Z,13Z,16Z)/18:4(6Z,9Z,12Z,15Z)) | Lipid Neg | 92.56 | C46 H74 N O10 P | 5.587 | LMGP03010803 |
| PS(22:4(7Z,10Z,13Z,16Z)/19:0) | Lipid Neg | 87.79 | C47 H84 N O10 P | 6.015 | LMGP03010804 |
| PS(22:4(7Z,10Z,13Z,16Z)/19:0) | Lipid Neg | 96.52 | C47 H84 N O10 P | 6.654 | LMGP03010804 |
| PS(22:4(7Z,10Z,13Z,16Z)/19:1(9Z)) | Lipid Neg | 99.88 | C47 H82 N O10 P | 5.574 | LMGP03010805 |
| PS(22:4(7Z,10Z,13Z,16Z)/21:0) | Lipid Neg | 98.31 | C49 H88 N O10 P | 6.488 | LMGP03010812 |
| PS(22:6(4Z,7Z,10Z,13Z,16Z,19Z)/0:0) | Lipid Neg | 76.72 | C28 H44 N O9 P | 1.828 | LMGP03050013 |
| PS(22:6(4Z,7Z,10Z,13Z,16Z,19Z)/19:0) | Lipid Neg | 94.91 | C47 H80 N O10 P | 5.750 | LMGP03010833 |
| PS(22:6(4Z,7Z,10Z,13Z,16Z,19Z)/19:0) | Lipid Neg | 97.12 | C47 H80 N O10 P | 4.933 | LMGP03010833 |
| PS(22:6(4Z,7Z,10Z,13Z,16Z,19Z)/19:0) | Lipid Neg | 97.58 | C47 H80 N O10 P | 5.011 | LMGP03010833 |
| PS(22:6(4Z,7Z,10Z,13Z,16Z,19Z)/21:0) | Lipid Neg | 96.95 | C49 H84 N O10 P | 6.476 | LMGP03010841 |
| PS(22:6(4Z,7Z,10Z,13Z,16Z,19Z)/21:0) | Lipid Neg | 98.72 | C49 H84 N O10 P | 5.663 | LMGP03010841 |
| PS(O-16:0/18:3(6Z,9Z,12Z)) | Lipid Neg | 94.86 | C40 H74 N O9 P | 5.595 | LMGP03020078 |
| PS(O-18:0) | Lipid Pos | 81.14 | C24 H50 N O8 P | 1.357 | LMGP03060002 |
| PS(O-18:0/0:0) | Lipid Neg | 98.24 | C24 H50 N O8 P | 1.706 | LMGP03060002 |
| PS(O-20:0) | Lipid Pos | 92.78 | C26 H54 N O8 P | 1.676 | LMGP03060001 |
| PS(O-20:0/17:0) | Lipid Neg | 96.24 | C43 H86 N O9 P | 7.060 | LMGP03020048 |
| PS(O-20:0/18:2(9Z,12Z)) | Lipid Pos | 79.60 | C44 H84 N O9 P | 5.310 | LMGP03020053 |
| PS(O-20:0/20:5(5Z,8Z,11Z,14Z,17Z)) | Lipid Neg | 87.05 | C46 H82 N O9 P | 6.595 | LMGP03020064 |
| PS(O-20:0/22:6(4Z,7Z,10Z,13Z,16Z,19Z)) | Lipid Neg | 96.55 | C48 H84 N O9 P | 7.009 | LMGP03020093 |
| PS(P-16:0/22:4(7Z,10Z,13Z,16Z)) | Lipid Neg | 90.93 | C44 H78 N O9 P | 5.872 | LMGP03030027 |
| PS(P-20:0/17:1(9Z)) | Lipid Neg | 98.60 | C43 H82 N O9 P | 6.222 | LMGP03030065 |
| PS(P-20:0/20:1(11Z)) | Lipid Pos | 75.48 | C46 H88 N O9 P | 6.037 | LMGP03030076 |
| Purine | Aqueous | 80.95 | C5 H4 N4 | 0.533 | HMDB01366 |
| Purine | Aqueous | 86.62 | C5 H4 N4 | 6.606 | HMDB01366 |
| Purine | Lipid Neg | 87.61 | C5 H4 N4 | 0.359 | HMDB01366 |
| Pyropheophytin b | Lipid Neg | 91.69 | C53 H70 N4 O4 | 5.582 | HMDB34248 |
| Pyrroline hydroxycarboxylic acid | Aqueous | 86.93 | C5 H7 N O3 | 4.498 | HMDB01369 |
| Quinoxaline | Lipid Pos | 92.37 | C8 H6 N2 | 0.881 | CAS: 91-19-0 |
| Rhamnulose | Aqueous | 90.13 | C6 H12 O5 | 0.607 | HMDB10207 |
| Roxithromycin | Lipid Neg | 94.98 | C41 H76 N2 O15 | 4.744 | HMDB14916 |
| Salannin | Lipid Pos | 98.18 | C34 H44 O9 | 1.353 | CAS: 992-20-1 |
| Sapelin A | Lipid Pos | 90.09 | C30 H50 O4 | 3.284 | CAS: 26790-93-2 |
| Sintaxanthin | Lipid Pos | 84.47 | C31 H42 O | 2.262 | HMDB35640 |
| SM(d16:1/24:0) | Lipid Neg | 85.20 | C45 H91 N2 O6 P | 3.728 | LMSP03010073 |
| SM(d18:0/18:2) | Lipid Neg | 71.27 | C41 H81 N2 O6 P | 5.290 | LMSP03010049 |
| SM(d18:1/16:0) | Aqueous | 89.21 | C39 H79 N2 O6 P | 3.715 | LMSP03010003 |
| SM(d18:1/18:1(9Z)) | Lipid Neg | 98.35 | C41 H81 N2 O6 P | 5.425 | LMSP03010029 |
| SM(d18:1/24:1(15Z)) | Aqueous | 86.21 | C47 H93 N2 O6 P | 3.700 | HMDB12107 |
| SM(d18:1/24:1(15Z)) | Lipid Neg | 92.06 | C47 H93 N2 O6 P | 7.622 | HMDB12107 |
| SM(d18:1/24:1(15Z)) | Lipid Neg | 93.15 | C47 H93 N2 O6 P | 7.326 | HMDB12107 |
| SM(d18:2/21:0) | Lipid Neg | 83.70 | C44 H87 N2 O6 P | 2.867 | LMSP03010064 |
| SM(d18:2/23:0) | Lipid Neg | 94.59 | C46 H91 N2 O6 P | 7.413 | LMSP03010075 |
| SM(d18:2/24:0) | Lipid Neg | 97.82 | C47 H93 N2 O6 P | 7.327 | LMSP03010081 |
| SM(d19:1/20:0) | Lipid Neg | 82.64 | C44 H89 N2 O6 P | 3.363 | LMSP03010068 |
| Sodium Tetradecyl Sulfate | Lipid Neg | 76.24 | C14 H30 O4 S | 2.027 | HMDB14607 |
| Sodium Tetradecyl Sulfate | Lipid Pos | 71.40 | C14 H30 O4 S | 1.169 | HMDB14607 |
| Sorbitol | Aqueous | 99.86 | C6 H14 O6 | 1.037 | KEGG: C00794 |
| Sorbose | Aqueous | 99.22 | C6 H12 O6 | 0.914 | HMDB01266 |
| Sorbose | Aqueous | 99.43 | C6 H12 O6 | 0.862 | HMDB01266 |
| Sorgolactone | Lipid Pos | 81.98 | C18 H20 O5 | 0.982 | CAS: 141262-39-7 |
| Sphinganine | Lipid Pos | 79.02 | C18 H39 N O2 | 3.863 | LMSP01020001 |
| Sphinganine | Lipid Pos | 84.08 | C18 H39 N O2 | 2.860 | LMSP01020001 |
| Sphingosine 1-phosphate | Lipid Neg | 82.10 | C18 H38 N O5 P | 1.760 | HMDB00277 |
| S-Ribosyl-L-homocysteine | Lipid Pos | 85.02 | C9 H17 N O6 S | 1.541 | KEGG: C03539 |
| Stearoyl-EA | Lipid Pos | 93.67 | C20 H41 N O2 | 2.966 | LMFA08040051 |
| Stearyl alcohol | Lipid Pos | 74.75 | C18 H38 O | 3.903 | LMFA05000085 |
| Stearyl citrate | Aqueous | 71.14 | C24 H44 O7 | 2.267 | HMDB32521 |
| Stigmast-5,22E-dien-3beta-yl (13Z,16Z,19Z-docosatrienoate) | Lipid Neg | 82.55 | C51 H84 O2 | 4.019 | LMST01020088 |
| Styrene | Lipid Pos | 85.05 | C8 H8 | 1.580 | CAS: 100-42-5 |
| Succinylcholine | Lipid Pos | 86.79 | C14 H30 N2 O4 | 1.029 | HMDB14347 |
| Tangeraxanthin | Lipid Pos | 90.43 | C34 H44 O2 | 1.518 | HMDB39015 |
| Taurine* | Aqueous | 98.80 | C2 H7 N O3 S | 1.569 | HMDB00251 |
| Terephthalic acid | Lipid Pos | 87.78 | C8 H6 O4 | 1.352 | HMDB02428 |
| Termitomycesphin A | Aqueous | 84.47 | C41 H77 N O10 | 2.448 | LMSP01080015 |
| Testosterone | Lipid Pos | 74.63 | C19 H28 O2 | 2.069 | LMST02020002 |
| Tetradecanyl 9Z,12Z-octadecadienoate | Lipid Neg | 99.33 | C32 H60 O2 | 2.868 | LMFA07010111 |
| TG(12:0/12:0/12:0) | Lipid Pos | 91.01 | C39 H74 O6 | 6.811 | HMDB11188 |
| TG(14:0/o-36:0) | Lipid Pos | 94.28 | C53 H104 O5 | 3.175 | HMDB42934 |
| TG(18:1(9Z)/15:0/22:6(4Z,7Z,10Z,13Z,16Z,19Z)) | Lipid Pos | 71.91 | C58 H98 O6 | 1.029 | HMDB49739 |
| TG(18:3/20:1/22:6) | Lipid Neg | 88.42 | C63 H102 O6 | 3.726 | HMDB53011 |
| TG(18:4/18:3/20:5) | Aqueous | 91.06 | C59 H90 O6 | 3.705 | HMDB55455 |
| TG(24:1/24:0/22:6) | Lipid Neg | 85.31 | C73 H128 O6 | 3.613 | HMDB52027 |
| TG(36:0) | Lipid Pos | 91.09 | C39 H74 O6 | 6.811 | HMDB11188 |
| TG(45:3) | Lipid Pos | 93.20 | C48 H86 O6 | 8.244 | LMGL03013276 |
| Threoninyl-Threonine | Lipid Neg | 75.11 | C8 H16 N2 O5 | 3.369 | HMDB29071 |
| Thromboxane | Lipid Pos | 84.11 | C20 H40 O | 1.354 | HMDB03208 |
| Trans-1,2-dimethylcyclohexane | Lipid Neg | 98.84 | C8 H16 | 3.615 | LMFA11000637 |
| Trans-2-Dodecenoylcarnitine | Lipid Pos | 91.14 | C19 H35 N O4 | 3.176 | HMDB13326 |
| Trans-Resveratrol 3,5-disulfate | Lipid Neg | 74.61 | C14 H12 O9 S2 | 1.674 | HMDB41781 |
| Triamiphos | Lipid Pos | 81.11 | C12 H19 N6 O P | 0.770 | CAS: 1031-47-6 |
| Tributyl phosphate* | Lipid Pos | 92.67 | C12 H27 O4 P | 1.312 | KEGG: C14439 |
| Trichostatin | Lipid Neg | 78.31 | C17 H22 N2 O3 | 1.363 | LMPK01000055 |
| Tricosanedioic acid | Aqueous | 75.55 | C23 H44 O4 | 1.267 | LMFA01170038 |
| Tridihexethyl | Lipid Pos | 75.86 | C21 H36 N O | 1.791 | HMDB14648 |
| Trifluoroacetic acid | Lipid Neg | 87.99 | C2 H F3 O2 | 0.344 | HMDB14118 |
| Triptolide | Lipid Pos | 97.29 | C20 H24 O6 | 1.220 | CAS: 38748-32-2 |
| Tris(2-butoxyethyl) phosphate* | Lipid Pos | 80.36 | C18 H39 O7 P | 1.349 | KEGG: C14446 |
| Tuberculostearic acid | Lipid Neg | 94.01 | C19 H38 O2 | 4.433 | HMDB04085 |
| TyrMe-Phe-OH | Lipid Pos | 70.17 | C25 H24 N2 O7 | 1.696 | METLIN: 65019 |
| Tyrosine* | Aqueous | 82.81 | C9 H11 N O3 | 2.005 | HMDB00158 |
| Umbelliferone | Lipid Pos | 78.60 | C9 H6 O3 | 10.564 | HMDB29865 |
| Ureidoisobutyric acid | Aqueous | 80.05 | C5 H10 N2 O3 | 4.480 | HMDB02031 |
| Ureidoisobutyric acid | Aqueous | 86.57 | C5 H10 N2 O3 | 4.655 | HMDB02031 |
| Uvaricin | Lipid Pos | 87.69 | C39 H68 O7 | 3.283 | CAS: 82064-83-3 |
| Val Val Trp | Lipid Pos | 75.79 | C21 H30 N4 O4 | 1.491 | METLIN: 19855 |
| Valeroyl Salicylate | Lipid Pos | 94.73 | C12 H14 O4 | 1.352 | CAS: 64206-54-8 |
| Valine | Aqueous | 99.64 | C5 H11 N O2 | 4.092 | HMDB00883 |
| Valtratum | Lipid Neg | 99.70 | C22 H30 O8 | 0.841 | KEGG: C09801 |
| Varanic acid | Lipid Pos | 87.53 | C26 H44 O5 | 3.181 | HMDB02195 |
| Varanic acid | Lipid Pos | 92.59 | C26 H44 O5 | 3.722 | HMDB02195 |
| Vitamin A2 aldehyde | Lipid Pos | 95.22 | C20 H26 O | 2.389 | HMDB35695 |
| Vitamin K1 | Lipid Pos | 70.14 | C31 H46 O2 | 2.651 | HMDB03555 |
